# Juglone-Bearing Thiopyrano[2,3-d]thiazoles Induce Apoptosis in Colorectal Adenocarcinoma Cells

**DOI:** 10.3390/cells14060465

**Published:** 2025-03-20

**Authors:** Yuliia Kozak, Nataliya Finiuk, Robert Czarnomysy, Agnieszka Gornowicz, Roman Pinyazhko, Andrii Lozynskyi, Serhii Holota, Olga Klyuchivska, Andriy Karkhut, Svyatoslav Polovkovych, Mykola Klishch, Rostyslav Stoika, Roman Lesyk, Krzysztof Bielawski, Anna Bielawska

**Affiliations:** 1 Department of Regulation of Cell Proliferation and Apoptosis, Institute of Cell Biology of National Academy of Sciences of Ukraine, Drahomanov 14/16, 79005 Lviv, Ukraine; nataliyafiniuk@gmail.com (N.F.); zorepad1775@gmail.com (O.K.); mykola.klishch1707@gmail.com (M.K.); stoika.rostyslav@gmail.com (R.S.); 2 Department of Synthesis and Technology of Drugs, Faculty of Pharmacy, Medical University of Bialystok, Kilinskiego 1, 15-089 Białystok, Poland; robert.czarnomysy@umb.edu.pl (R.C.); krzysztof.bielawski@umb.edu.pl (K.B.); 3 Department of Biotechnology, Faculty of Pharmacy, Medical University of Bialystok, Kilinskiego 1, 15-089 Białystok, Poland; anna.bielawska@umb.edu.pl; 4 Department of Normal Physiology, Organic and Bioorganic Chemistry, Danylo Halytsky Lviv National Medical University, Pekarska 69, 79010 Lviv, Ukraine; pinyazhkoroman@gmail.com; 5 Department of Pharmaceutical, Organic and Bioorganic Chemistry, Danylo Halytsky Lviv National Medical University, Pekarska 69, 79010 Lviv, Ukraine; lozynskyiandrii@gmail.com (A.L.); golota_serg@yahoo.com (S.H.); dr_r_lesyk@org.lviv.net (R.L.); 6 Department of Technology of Biologically Active Substances, Pharmacy and Biotechnology, Lviv Polytecnic National University, Bandera 12, 79013 Lviv, Ukraine; andrew.karkhut@gmail.com (A.K.); spolovkovych@ukr.net (S.P.); 7 Department of Biotechnology and Cell Biology, Medical College, University of Information Technology and Management in Rzeszów, Sucharskiego 2, 35-225 Rzeszów, Poland

**Keywords:** colorectal cancer, thiopyrano[2,3-d]thiazoles, apoptosis, proliferation, reactive oxygen species

## Abstract

Colorectal cancer is a major global health challenge, with current treatments limited by toxicity and resistance. Thiazole derivatives, known for their bioactivity, are emerging as promising alternatives. Juglone (5-hydroxy-1,4-naphthoquinone) is a naturally occurring compound with known anticancer properties, and its incorporation into thiopyrano[2,3-d]thiazole scaffolds may enhance their therapeutic potential. This study examined the cytotoxicity of thiopyrano[2,3-d]thiazoles and their effects on apoptosis in colorectal cancer cells. Les-6547 and Les-6557 increased the population of ROS-positive HT-29 cancer cells approximately 10-fold compared with control cells (36.3% and 38.5% vs. 3.8%, respectively), potentially contributing to various downstream effects. Elevated ROS levels were associated with cell cycle arrest, inhibition of DNA biosynthesis, and reduced cell proliferation. A significant shift in the cell cycle distribution was observed, with an increase in S-phase (from 17.3% in the control to 34.7% to 51.3% for Les-6547 and Les-6557, respectively) and G2/M phase (from 24.3% to 39.9% and 28.8%). Additionally, Les-6547 and Les-6557 inhibited DNA biosynthesis in HT-29 cells, with IC_50_ values of 2.21 µM and 2.91 µM, respectively. Additionally, ROS generation may initiate the intrinsic apoptotic pathway. **Les-6547** and **Les-6557** activated both intrinsic and extrinsic apoptotic pathways, demonstrated by notable increases in the activity of caspase 3/7, 8, 9, and 10. This study provides a robust basis for investigating the detailed molecular mechanisms of action and therapeutic potential of Les-6547 and Les-6557.

## 1. Introduction

According to the International Agency for Research on Cancer (IARC), colorectal cancer is the third most common malignancy in both men and women, after lung cancer, female breast cancer, and male prostate cancer [1]. As of 2022, colorectal cancer accounted for 9.3% of cancer-related deaths globally, ranking it second by mortality after lung cancer [2]. Colonoscopy screening has contributed to a gradual decrease in colorectal cancer incidence in developed countries. Nevertheless, colorectal cancer remains a severe public health issue, and its occurrence in adults below the age of 50 is increasing [3,4,5].

The therapeutic approaches towards colorectal cancer include surgery, chemotherapy, radiotherapy, targeted therapy, and immunotherapy. Radiation therapy is commonly used for treating rectal cancer, but this approach is seldom employed in colon cancer treatment. Surgical resection remains the standard approach, primarily for localized early-stage colorectal cancer (stages 0, I, II). However, chemotherapy is often necessary in addition to surgery, especially at the later stages of cancer progression (stage III, IV, and high-risk stage II) [2].

Chemotherapy is most commonly used after surgical resection of tumors (adjuvant chemotherapy) for treating stage III, IV, and some stage II colon cancers [6]. Therapeutic agents typically used for adjuvant chemotherapy include 5-fluorouracil (5-FU), leucovorin (LV), oxaliplatin, capecitabine, and irinotecan. In most cases, these are used in combinations of two to four drugs as part of a treatment regimen. For example, curative resection of stage III colorectal cancer is often followed by a FOLFOX (5-FU, leucovorin, and oxaliplatin) or CapeOx (capecitabine and oxaliplatin) regimen [7]. Oxaliplatin alone has a very limited efficacy for colorectal cancer. However, when it is combined with 5-FU and LV, it increases the therapeutic effect of the regimen, which has been demonstrated in phase III clinical trials [2,8].

In addition to conventional treatments, focused ultrasound therapy (FUS) has emerged as a promising non-invasive technique for colorectal cancer treatment [9]. The safety and effectiveness of FUS in treating colorectal cancer have been established by preclinical and clinical research [10]. FUS enhances the targeted delivery of chemotherapeutics, including 5-fluorouracil, oxaliplatin, and doxorubicin, resulting in more effective tumor reduction and improved survival outcomes in animal models [11]. FUS may also modulate immune response and disrupt the tumor microenvironment [9].

Neoadjuvant chemotherapy and neoadjuvant immunotherapy are among the new therapeutic approaches that are prescribed before the surgery. The FOxTROT phase III trial is the most notable recent example of neoadjuvant chemotherapy for locally advanced colon cancer [12,13]. Neoadjuvant immunotherapy utilizes the monoclonal antibody drugs nivolumab, pembrolizumab, and ipilimumab to target programmed cell death 1 receptor (PD-1) and cytotoxic T-cell-associated protein 4 (CTLA-4) expressed by CD8+ T-lymphocytes, to enhance antitumor immunity [14]. The epidermal growth factor receptor (EGFR) on cancer cells is the target of another therapeutic approach. The efficacy of EGFR-specific monoclonal antibody drugs panitumumab and cetuximab as single agents and in combination with 5-FU, LV, and irinotecan has been evaluated in several phase III clinical trials [15,16,17].

As well as the aforementioned therapies, several novel therapeutic approaches for colorectal cancer are being studied and developed. Colorectal cancer cells are often characterized by mutations in the *KRAS* oncogene *TP53* tumor suppressor, which has been investigated as a potential target for gene therapy [18]. The potential of applying CRISPR/Cas9 in gene therapy for colorectal cancer is also being investigated [19]. The development of novel colon cancer therapies can greatly benefit from novel methods for in vitro screening, such as microphysiological systems [20].

Among the problems relating to the chemotherapeutic agents currently used for colorectal cancer treatment are their off-target effects and toxic side effects, such as neurotoxicity, gastrointestinal toxicity, hand–foot syndrome, diarrhea, nausea, vomiting, neutropenia, thrombocytopenia, and other hematologic disorders [21,22,23]. This emphasizes the importance of drug discovery and developing new, more selective chemotherapeutics with lower general toxicity and an improved therapeutic index.

Thiazole derivatives and their structure-related analogues are of special interest in relation to the discovery of novel anticancer drugs. The thiazole ring has been proven to be a suitable scaffold for bioactive compounds. As of 2021, there were 18 FDA-approved therapeutic agents and numerous experimental drugs containing the thiazole cycle [24]. Fused thiazole derivatives also deserve attention in the search for potential biologically active compounds. These compounds possess a wide range of biological activities, with many demonstrating anticancer properties; for some of these compounds, potential bio-targets have also been established [25]. A study by Sabry et al. evaluated imidazo[2,1-b]thiazole derivatives as inhibitors of the tyrosine kinase receptor EGFR, a crucial therapeutic target in colorectal cancer therapy [26]. Interest in studying thiopyrano[2,3-d]thiazole derivatives arises from their potential as antineoplastic agents, incorporating various pharmacophore moieties, including fragments of natural molecules such as naphthoquinone derivatives. In certain thiopyrano[2,3-d]thiazole derivatives, various antineoplastic mechanisms have been identified; notably, these include the inhibition of transforming growth factor beta (TGF-β) [27], human carbonic anhydrase IX and XII [28], and tubulin polymerization [29], and the activation of PPARγ receptors [30].

In recent work [27], we synthesized and described the anticancer activity of thiopyrano[2,3-d]thiazoles based on 5-hydroxy-1,4-naphthoquinone (juglone) towards a panel of tumor cell lines from different tissues, pseudonormal cells, and isolated peripheral blood lymphocytes from a healthy human donor. It is important to note that juglone itself has antiproliferative properties. It induces apoptosis and autophagy by modulating mitogen-activated protein kinase pathways in human hepatocellular carcinoma cells [31]. Additionally, juglone exhibits antiproliferative effects against human cervical carcinoma HeLa cells through the activation of caspase 9, 8, and 3, and cleavage of PARP [32].

The synthesis of two juglone derivatives, Les-6547 (compound **3.1**) and Les-6557 (compound **3.3**), as well as their impacts on cell viability in colorectal, breast, and cervical cancers, chronic myelogenous leukemia, and pseudonormal cells, have previously been evaluated [27]. HCT-116 colorectal cancer cells showed the highest sensitivity to Les-6547 and Les-6557, while pseudonormal cells displayed much lower sensitivity, indicating the selectivity of their action.

Therefore, the objective of the present study was to evaluate in silico the drug-likeness and pharmacokinetic properties of juglone-bearing thiopyrano[2,3-d]thiazoles Les-6547 and Les-6557, to study in vitro their cytotoxic activity effects on cell proliferation, apoptosis, cell cycle regulation, and reactive oxygen species (ROS) production. The present work is a part of our exploration of the pharmacological potential of thiopyrano[2,3-d]thiazoles; herein, we emphasize the prospects for their molecular hybridization with natural frameworks and scaffolds for the design of drug-like molecules.

## 2. Materials and Methods

### 2.1. Materials

All chemicals and solvents used were of analytical grade and obtained from commercial suppliers.

The materials used in this study included 3-(4,5-dimethylthiazol-2-yl)-2,5-diphenyltetrazolium bromide (MTT), dimethyl sulfoxide (DMSO), methanol, phytohemagglutinin-L (PHA-L), Hoechst-33342, DHE, crystal violet, and doxorubicin, all purchased from Sigma-Aldrich (St. Louis, MO, USA). McCoy’s 5A medium was provided by PAN-Biotech (Aidenbach, Germany), sodium heparin solution by B. Braun Medical, S.A. (Rubí, Spain), and Gradisol G by Polfa (Warsaw, Poland).

HT-29 and DLD-1 colorectal adenocarcinoma cells, normal human keratinocytes of the HaCaT line, and murine fibroblasts of the Balb/c 3T3 line were obtained from the American Type Culture Collection (ATCC, Manassas, VA, USA). For cell culture, Dulbecco’s Minimal Eagle Medium (DMEM), RPMI-1640 medium, fetal bovine serum (FBS), phosphate-buffered saline (PBS), 0.05% trypsin with 0.02% EDTA, glutamine, and penicillin/streptomycin cocktail were sourced from Gibco (San Diego, CA, USA).

To assess biological activity, [^3^H]-thymidine (7 Ci/mmol) was acquired from Moravek Biochemicals (Brea, CA, USA), and the Ultima Gold XR scintillation cocktail was supplied by PerkinElmer (Waltham, MA, USA). Apoptosis was evaluated using an FITC Annexin V Apoptosis Detection Kit II, JC-1 MitoScreen Kit, and Stain Buffer from BD Pharmingen (San Diego, CA, USA). The FAM-FLICA^®^ Caspase 3/7, Caspase 8, Caspase 9, and Caspase 10 Assay kits, Intracellular Total ROS Activity Assay Kit, and propidium iodide (PI) were obtained from ImmunoChemistry Technologies (Bloomington, MN, USA). The DNase-free RNase A Solution was acquired from Promega (Madison, WI, USA).

### 2.2. Tested Compounds

The synthesis and characterization of the compounds Les-6547 and Les-6557, referred to as compounds **3.1** and **3.3**, respectively, were reported in a previous study [21]. Synthesis of compounds Les-6547 and Les-6557 was performed via regioselective hetero-Diels–Alder reaction using (Z)-5-(4-chlorobenzylidene)- and (Z)-5-(5-chloro-2-hydroxybenzylidene)-4-thioxothiazolidin-2-ones as dienes and juglone as a dienophile. Target molecules were obtained by reflux of starting reagents in the glacial acetic acid medium with the presence of hydroquinone [27].

Solutions of the compounds were prepared in DMSO at 20 mM concentration. Interim dilutions of the compounds were made in a medium and immediately introduced to the cells.

### 2.3. Cell Cultures

DLD-1, HaCaT, and Balb/c 3T3 cells were maintained in DMEM, while HT-29 cells were grown in McCoy’s 5A medium with 10% FBS and 1% penicillin/streptomycin. All cell lines were incubated at 37 °C in a humidified atmosphere with 5% CO_2_. After detaching the cells with trypsin, they were quantified using a Scepter 3.0 automated cell counter (Millipore, Burlington, MA, USA).

### 2.4. Lymphocytes from Human Peripheral Blood: Isolation and Culture

Lymphocytes from human peripheral blood were isolated with sodium heparin (10 U/mL) as an anticoagulant. Isolation was performed through a density gradient method with the Gradisol G, following a modified procedure. Peripheral blood was mixed with Gradisol G in equal proportions (1:1) and centrifuged at 400× *g* for 30 min at room temperature. The cells were collected, washed in phosphate-buffered saline (PBS), and treated with a hypotonic solution to remove residual erythrocytes. Lymphocytes were activated by phytohemagglutinin-L (1 µg/mL) and cultured in RPMI-1640 medium supplemented with 20% fetal bovine serum under controlled conditions of 95% air and 5% CO_2_ [27].

The research involving lymphocytes isolated from the peripheral blood of adult healthy donors was conducted in accordance with the principles outlined in the Declaration of Helsinki and approved by the Ethics Committee of the Institute of Cell Biology of the National Academy of Sciences of Ukraine (protocol No. 2023-1, dated 14 July 2023), after obtaining written informed consent from the donors.

The inclusion criteria for donor selection included male sex, an age range of 18–50 years, a body weight of 50–100 kg, and a body mass index (BMI) between 18.5 and 27. The exclusion criteria comprised a history of chronic diseases, amputations, organ transplants, or malignancies; the presence of acute illness within 14 days prior to sample collection; recent blood donation within the last 60 days or more than six donations within a year; body temperature exceeding 37 °C; or any visible signs of illness at the time of sample collection.

### 2.5. MTT Assay and Colony Forming Assay

The metabolic activity of cells exposed to the tested derivatives, doxorubicin, and DMSO, was assessed using an MTT assay according to the manufacturer’s protocol (Sigma-Aldrich, St. Louis, MO, USA). Adherent cells were seeded into 96-well plates (5000–10,000 cells in 100 µL of growth medium per well) and allowed to adhere overnight before treatment. For lymphocytes, activation with phytohemagglutinin-L (1 µg/mL) was performed for 24 h [33], after which they were seeded in 96-well plates at a density of 100,000 cells/well in RPMI-1640 medium supplemented with 20% FBS. The test substances, including Les-6547, Les-6557, and doxorubicin, were added to 100 µL of culture medium to achieve final concentrations of 0 µM, 0.1 µM, 1 µM, 2.5 µM, 10 µM, and 100 µM for all compounds, and the cells were further incubated for 24, 48, or 72 h. DMSO-treated cells served as the control. Then, 20 µL of MTT solution (5 mg/mL) was added and incubated for 2–4 h to allow formazan crystal formation. The formazan crystals were then solubilized by adding DMSO. The absorbance of formazan was measured at 570 nm using a ThermoScientific Evolution 201 UV-VIS spectrophotometer (ThermoFisher Scientific, Waltham, MA, USA). The control condition with 0.5% DMSO was considered 100% viability. The IC_50_ values for the compounds were determined using GraphPad Prism 8 and 9 software (GraphPad Software, Boston, MA, USA).

For colony forming assay, HT-29 and DLD-1 cells were seeded in 12-well plates at a density of 500 cells in 2 mL of growth medium per well. After overnight incubation, the cells were exposed to the compounds Les-6547, Les-6557, and doxorubicin at concentrations ranging from 0.1 to 100 µM for 72 h. Following treatment, the medium was replaced with fresh medium without the compounds. After 14 days of culture, cells were rinsed with PBS and fixed with cold methanol for 10 min. Subsequently, the cells were stained with a 0.1% crystal violet solution for 10 min. After washing, the plates were allowed to dry at room temperature [34]. Numbers of colonies were quantified using ImageJ software (version 1.5.4; National Institutes of Health, Bethesda, MD, USA). The effect of the compounds on the colony-forming capacity was calculated as a percentage of colonies relative to the untreated control group.

### 2.6. Apoptosis Assay

The pro-apoptotic effects of the tested compounds were assessed using the FITC Annexin V Apoptosis Detection Kit II in conjunction with a BD FACSCanto II flow cytometer (BD Biosciences, San Jose, CA, USA). The experimental procedure adhered to the manufacturer’s instructions and previously established protocols [35].

Initially, HT-29 cells were seeded in 6-well plates at a density of 300,000 cells per well in 2 mL of culture medium and incubated overnight to allow adherence. Subsequently, the cells were treated with Les-6547 (5 µM) and Les-6557 (10 µM) for 24 h. After exposure to the compounds, the cells were rinsed twice with cold PBS and suspended in the binding buffer provided in the kit. FITC Annexin V and propidium iodide (PI) were added to the 100 µL cell suspension, followed by 15-min incubation at room temperature, shielded from light.

After the incubation, 300 µL of binding buffer was added to each sample, and analysis was performed using the flow cytometer, with 10,000 events recorded per sample. Data were processed using FACSDiva software (version 6.1.3, BD Biosciences, San Jose, CA, USA). Calibration of the instrument was ensured using a BD Cytometer Setup and Tracking Beads (BD Biosciences, San Diego, CA, USA) [35].

### 2.7. Mitochondrial Membrane Potential Assay

The disruption of mitochondrial membrane potential (MMP) was assessed using the JC-1 MitoScreen kit and a BD FACSCanto II flow cytometer, following the instructions provided and previously described protocols [35].

HT-29 cells were exposed to Les-6547 (5 µM) and Les-6557 (10 µM) for 24 h. After the treatment, the cells were washed and resuspended in 0.5 mL of buffer containing 10 μg/mL JC-1, followed by incubation for 15 min at room temperature, shielded from light.

Then, the cells were washed twice with buffer, resuspended in 300 μL of PBS, and analyzed for mitochondrial membrane potential disruption. The percentage of cells exhibiting disrupted MMP was determined using FACSDiva 6.1.3 software (BD Biosciences, San Jose, CA, USA) [35].

### 2.8. Caspase 3/7, 8, 9, and 10 Activity Assays

Caspase activity for initiator caspases (8, 9, and 10) and executioner caspases (3/7) was evaluated using a FAM-FLICA^®^ Caspase Assay kit (ImmunoChemistry Technologies, Bloomington, MN, USA) according to the manufacturer’s instructions, as described in previous studies [35]. HT-29 cells were exposed to Les-6547 (5 µM) and Les-6557 (10 µM) for 24 h. Following this, the cells were harvested, washed twice with cold PBS, and resuspended in Apoptosis Wash Buffer (ImmunoChemistry Technologies, Bloomington, MN, USA) at a final concentration of 5 × 10^5^ cells/mL.

Subsequently, 290 µL of the cell suspension was transferred into individual tubes, to which 10 µL of freshly prepared FLICA solution (1:5 dilution in PBS) was added. The mixture was gently pipetted and incubated for 1 h at 37 °C in the dark. After the incubation, the cells were washed twice with 2 mL of Apoptosis Wash Buffer, centrifuged, and resuspended in 300 µL of the same buffer. The prepared samples were immediately analyzed using a BD FACSCanto II flow cytometer (10,000 events). Data were processed using FACSDiva 6.1.3 software (BD Biosciences Systems, San Jose, CA, USA). Flow cytometer calibration was conducted using a BD Cytometer Setup and Tracking Beads (BD Biosciences, San Diego, CA, USA) [35].

### 2.9. Cell Cycle Analysis

Cell cycle analysis of HT-29 cells treated with Les-6547 (5 µM) and Les-6557 (10 µM) for 24 h was conducted utilizing a FACSCanto II flow cytometer. Following the incubation period, the cells were detached using trypsin, collected, and fixed in cold ethanol (70%) before being stored at −20 °C for up to three days. Cells were then processed for flow cytometry analysis as previously described [29]. Fixed cells were washed with cold PBS and centrifuged at 2000 rpm for 10 min, and the supernatant was discarded. The cell pellet was resuspended in PBS with 50 μg/mL DNase-free RNase A Solution and stained with 100 μg/mL PI for 30 min at 37 °C in the dark. After washing, the cells were resuspended in PBS. Cell cycle distribution was analyzed using a BD FACSCanto II flow cytometer (10,000 events) and FACSDiva 6.1.3 software (both BD Biosciences Systems, San Jose, CA, USA), followed by analysis with FCS Express 7 software (De Novo Software, Pasadena, CA, USA). The system was calibrated using a BD Cytometer Setup and Tracking Beads (BD Biosciences, San Diego, CA, USA) [35].

### 2.10. Intracellular Total ROS Activity Assay

Total reactive oxygen species (ROS) activity was assessed using an Intracellular Total ROS Activity Assay Kit. Following 24 h incubation with studied derivatives, HT-29 cells were prepared for analysis with the flow cytometer according to the protocol detailed in the literature [30]. The cells were washed twice with cold PBS, followed by the addition of the assay buffer provided in the kit. The cells, at a concentration of 1 × 10⁶ cells/mL, were then exposed to 10 µL of Total ROS Green reagent and incubated for one hour at 37 °C in a CO_2_ incubator. After the incubation period, the cells were rinsed with the assay buffer. Finally, the cells were resuspended in 500 µL of assay buffer and analyzed using a BD FACSCanto II flow cytometer in conjunction with FACSDiva 6.1.3 software (both BD Biosciences Systems, San Jose, CA, USA). The BD Cytometer Setup and Tracking Beads (BD Biosciences, San Diego, CA, USA) were used for calibration [36].

### 2.11. Fluorescent Microscopy

HT-29 cells were seeded on glass slides in 12-well plates and then treated with Les-6547 (5 µM) and Les-6557 (10 µM) for 24 h. Cells were additionally incubated for 20–30 min with Hoechst-33342 dye (0.5 µg/mL) and DHE (1 μM). Cells were analyzed using a Zeiss fluorescent microscope and an AxioImager A1 camera (400× magnification, Carl Zeiss, Jena, Germany). Image Pro 7 software (Media Cybernetics, Rockville, MD, USA) was used for photographic analyses [37]. Cell fluorescence was measured using ImageJ software 1.44p (National Institutes of Health, Bethesda, MD, USA).

### 2.12. Molecular Docking

Ligand structures were geometrically optimized in Avogadro v1.1.1 using the steepest descent algorithm under the UFF force field with the maximum 10^4^ energy minimization steps and convergence value of 10^–7^ kcal/mol·Å [38]. The structures were saved in CML format and converted to PDBQT format with OpenBabel v3.1.1 and prepare_ligand4.py Python v2.7.3 script from the MGLTools v1.5.7 package [39,40]. Upon conversion to PDBQT, only the polar hydrogen atoms were retained.

SwissTargetPrediction was utilized to identify the most likely targets for each ligand, based on the similarity principle [41]. The input consisted of SMILES strings generated from CML files with OpenBabel v3.1.1 [42]. Targets with a predicted probability greater than 0% that were known to be involved in apoptosis, proliferation, and oncogenic transformation were selected for further analysis. For each selected protein target, identified by its ChEMBL ID, multiple PDB IDs were retrieved from the ChEMBL API [43]. The corresponding human protein structures were obtained from the RCSB PDB database based on their PDB IDs [44]. PDB structures with co-crystallized drug-like or drug ligands were selected for further processing.

The PDB structures were stripped of water molecules, co-crystallized ligands, and cofactors using the PDB parsing tool from the Biopython v1.85 package [45]. This approach was applied uniformly to all PDB structures because of the limited capability of automated methods to reliably distinguish essential cofactors from competing co-crystallized ligands during the screening. The cleaned protein structures were converted from PDB to PDBQT format using the prepare_receptor4.py Python v2.7.3 script from the MGLTools v1.5.7 package [46].

Co-crystallized ligands with molecular weight greater than 200 Da were extracted from PDB structures and saved in PDBQT format to be used in calculating binding site dimensions and affinity comparison. The cutoff value of 200 Da was selected based on the molecular weight distribution in the sample of co-crystallized ligands in order to distinguish drug-like molecules from crystallization additives.

Molecular docking was performed in AutoDock Vina v1.2.3 [47,48] with an exhaustiveness value of 8 (default). The grid box center and dimensions were set to fully encompass the position of the co-crystallized ligand in its binding site with 5 Å extra margins. If the co-crystallized ligand was smaller in any dimension than the ligands of interest, the grid box dimension was set to the maximum length of the ligand of interest, augmented with a 5 Å margin. Both the docking poses and the positions of co-crystallized ligands bound to their sites were evaluated using the Vina scoring function, and the scores of the co-crystallized and docked ligands were compared.

### 2.13. Statistical Analysis

The results were analyzed with GraphPad Prism 8 and 9 (GraphPad Software, USA) and are presented as a mean (M) ± standard deviation (SD) of three independent experiments (*n* = 3) carried out in triplicate. A statistical evaluation was performed using two-way ANOVA analysis. A *p*-value of <0.05 was considered statistically significant. For the statistical analysis of molecular docking scores, one-way ANOVA was used.

## 3. Results

### 3.1. Thiopyrano[2,3-d]thiazoles Les-6547 and Les-6557 Exhibit Favorable ADMET Profiles In Silico

**Figure 1:**
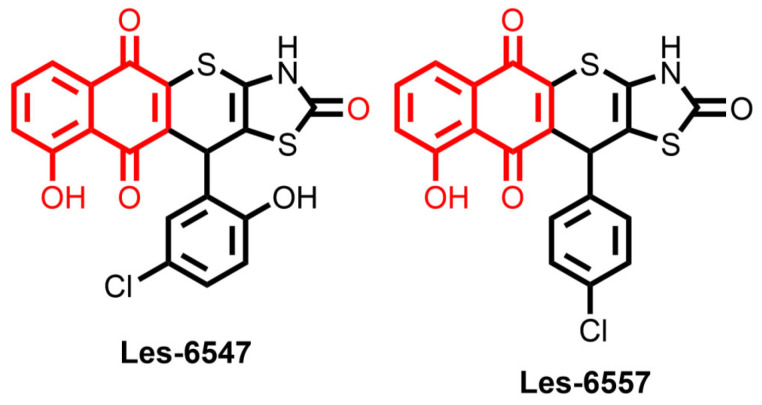
Structures of the studied thiopyrano[2,3-d]thiazoles Les-6547 and Les-6557 with juglone (5-hydroxy-1,4-naphthoquinone) moiety in the molecules colored in red.

Nowadays, the application of in silico simulations plays a key role in studies of small molecules as potential pharmacological agents. In this context, series of drug-likeness and ADMET properties were evaluated using ADMETLab 3.0 [49], for the juglone-bearing thiopyrano[2,3-d]thiazoles Les-6547 and Les-6557 (Figure 1).

The calculated prognostic ADMET parameters are summarized and presented in Table 1, Table 2 and Table 3. Both studied compounds meet requirements from a medicinal chemistry point of view and possess satisfactory parameter values according to the Lipinski and Pfizer rules; however, they break GSK rules with their excessive molecular weight value (limited to 400 by GSK rules) (Table 1).

Prognostically, neither molecule is a substrate to P-glycoprotein and both show a medium level of intestinal absorption. The calculated results suggest that compounds Les-6547 and Les-6557 do not pass the blood–brain barrier. Also, both tested derivatives are likely to have strong plasma protein binding (Table 2).

Additionally, the calculated excretion and medium toxicity parameters (Table 3) for both compounds were excellent. Moreover, both compounds exhibited low plasma clearance and the predicted half-life was short, possibly indicating a need for less frequent dosing. Regarding toxicity, both molecules displayed low risk of hepatotoxicity and medium risk of mutagenicity, making them fairly safe candidates.

Moreover, computed data for Les-6547 and Les-6557 demonstrate poor probability of being agonists of aryl hydrocarbon receptor (AhR) as well as being activators of the antioxidant response element (ARE) signalling pathway, implying that they may not significantly influence mechanisms involved in detoxification, inflammation, or xenobiotic metabolism, which are typically regulated by AhR and ARE. These could suggest that the compounds are less likely to induce adverse effects related to these pathways, making them potentially safer in terms of toxicological concerns related to these receptors.

The excellent probability of being agonists to the androgen (AR) and peroxisome proliferator-activated gamma receptors (PPARγ) as well as the medium probability of being agonists to estrogen receptor (ER) and aromatase inhibitors was calculated for both compounds. This calculated impact on AR and PPARγ indicates that both compounds may influence pathways related to metabolic regulation, lipid metabolism, and potentially androgenic or anti-androgenic effects. The moderate likelihood of molecules Les-6547 and Les-6557 interacting with the ER and acting as aromatase inhibitors further suggests that Les-6547 and Les-6557 could have the potential to influence estrogenic activity. This could be particularly relevant in the context of hormone-dependent cancers, such as breast cancer, where inhibition of estrogen signaling may be a therapeutic strategy. However, additional experimental validation is necessary to confirm these computational predictions and to assess their safety and efficacy in biological systems.

### 3.2. Les-6547 and Les-6557 Exhibit Docking Scores Comparable to Ligands with Experimentally Validated Binding Capabilities

Since both Les-6547 and Les-6557 contain one asymmetric carbon atom, the enantiomers of Les-6547 and Les-6557 were analyzed separately, thus bringing the number of ligand structures to four (Les-6547-R, Les-6547-S, Les-6557-R, and Les-6557-S). SwissTargetPrediction predicted 84 potential protein targets for each enantiomer of Les-6557, while no targets were predicted for Les-6547 (Supplementary Table S1). This can be explained by minor structural differences between the compounds, which can impact predictions based on the similarity principle [41]. However, considering the high similarity between Les-6547 and Les-6557, both compounds were docked to targets predicted for Les-6557, thereby allowing comparison of the ligands. Six of the predicted targets were shortlisted based on their involvement in the regulation of apoptosis, proliferation, and oncogenesis and were included in the molecular docking studies (Table 4, Supplementary Tables S2–S7).

It should be noted that for CDK2, JAK2, and MAPK8, the studied thiopyrano[2,3-d]thiazole derivatives demonstrated more favorable docking scores than the co-crystallized ligands with experimentally confirmed binding properties. This suggests that the predicted interaction of Les-6547 and Les-6557 with these protein targets may be responsible for their cytotoxic and proapoptotic activity. CDK2, JAK2, and MAPK8 may be promising candidates for further in silico investigation and experimental validation in future studies.

**Figure 2:**
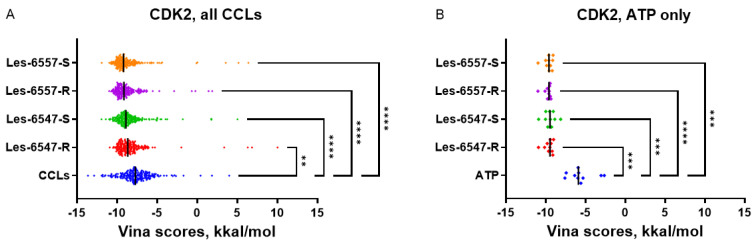
Docking scores for Les-6547 and Les-6557 for multiple CDK2 PDB structures compared with scores for a—all co-crystallized ligands (*n* = 202) and b—only co-crystallized ATP (*n* = 10, PDB IDs: 1B38, 1B39, 1FIN, 1FQ1, 1GY3, 1JST, 1QMZ, 2CCI, 2CJM, 8FP5). ** *p* < 0.01; *** *p* < 0.001; **** *p* < 0.0001 compared with the CCLs (**A**) and ATP (**B**).

The predicted capability of Les-6547 and Les-6557 to bind CDK2 at the same site that interacts with ATP and kinase inhibitors suggests that the studied thiopyrano[2,3-d]thiazoles may themselves act as kinase inhibitors. In the case of CDK2, the mean docking scores of Les-6547 and Les-6557 (Table 4) were more favorable than the Vina scores for co-crystallized ATP (Figure 2) but less favorable than the scores for co-crystallized non-selective kinase inhibitors like staurosporine (maximum Vina score 13.63 kkal/mol, PDB ID 7NVQ, Supplementary Table S2). In contrast to CDK2, the docking scores for CDK4 were comparable to scores for co-crystallized ATP (PDB IDs: 5FWK, 5FWL, 5FWM, 5FWP). This fact alone cannot prove Les-6547 and Les-6557 to be selective kinase inhibitors, but the kinase-inhibiting properties and inhibition selectivity of thiopyrano[2,3-d]thiazole derivatives may be promising topics for further investigation.

### 3.3. Thiopyrano[2,3-d]thiazoles’ Selective Cytotoxicity Toward Colorectal Cancer Cells

Derivatives Les-6547 and Les-6557 were examined for their cytotoxicity against colorectal adenocarcinoma cell lines HT-29 and DLD-1, pseudonormal murine fibroblasts of the Balb/c 3T3 line, pseudonormal human keratinocytes of the HaCaT line, and human lymphocytes isolated from the peripheral blood of a healthy donor after 24, 48, and 72 h of exposure using the MTT assay. Doxorubicin was used as a reference drug.

HT-29 and DLD-1 cell lines were selected as representative models of colorectal cancer due to their distinct molecular characteristics. HT-29 is a microsatellite-stable (MSS) and CpG island methylator phenotype-positive (CIMP+) cell line that harbors the BRAF (p.V600E) mutation. In contrast, DLD-1 is microsatellite-instable (MSI) and CIMP+, carrying the KRAS (p.G13D) mutation. These differences allowed broad assessment of the potential efficacy of the studied compounds in distinct colorectal cancer molecular subtypes [50].

**Figure 3:**
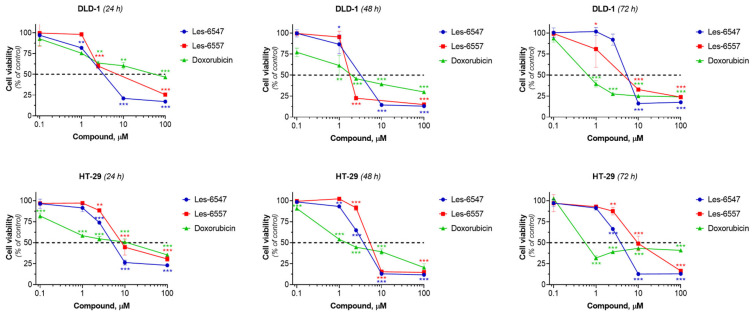
Viability of HT-29 and DLD-1 colorectal adenocarcinoma cells after 24, 48, and 72 h of treatment with Les-6547, Les-6557, and doxorubicin (reference drug). MTT assay data are expressed as a percentage of the control group and presented as mean ± SD of three independent experiments (*n* = 3) conducted in triplicate. * *p* < 0.05; ** *p* < 0.01; *** *p* < 0.001 compared with control cells.

Les-6547 and Les-6557 exhibited significant dose-dependent cytotoxicity towards both colorectal adenocarcinoma cell lines, HT-29 and DLD-1. The IC_50_ of Les-6547 was 4.29–5.72 µM for HT-29 cells and 5.14–6.62 µM for DLD-1 cells (Table 5, Figure 3). The IC_50_ of Les-6557 was 6.54–10.41 µM in HT-29 cells and 1.94–16.85 µM in DLD-1 cells.

The DLD-1 cell line is histologically similar to primary tumors. In contrast, the HT-29 cell line is commonly used to assess multidrug resistance, nutrient absorption, and chemically induced differentiation of enterocytes [51]. Despite these differences, both colorectal adenocarcinoma cell lines exhibited similar sensitivity to the studied compounds. Les-6547 demonstrated higher cytotoxicity towards HT-29 cells at all time points in the experiment, compared with Les-6557. A similar trend was observed after 24 h of incubation of DLD-1 cells with the studied agents. Then at the 48th hour of the study reaction, Les-6557 inhibited the viability of DLD-1 colorectal adenocarcinoma cells 2.5 times more effectively than Les-6547. It was observed that both Les-6547 and Les-6557 exhibited higher cytotoxic potency than the reference drug doxorubicin in both colorectal tumor cell lines after 24 h of exposure (Figure 3, Table 5).

**Figure 4:**
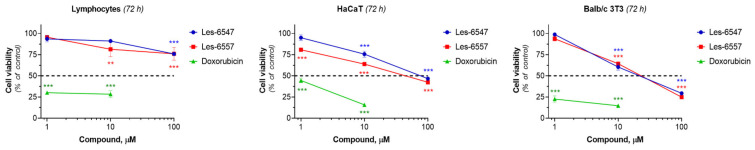
Viability of human lymphocytes isolated from the peripheral blood of healthy donors, HaCaT human keratinocytes, and Balb/c 3T3 murine fibroblasts after treatment for 72 h with Les-6547, Les-6557, and doxorubicin (reference drug). Data are expressed as a percentage of the control group and presented as a mean value ± SD of three independent experiments (*n* = 3) done in triplicate. ** *p* < 0.01; *** *p* < 0.001 compared with the control cells.

The extreme toxicity of many anticancer drugs to normal cells remains a serious concern. The effect of the studied derivatives on the viability of human lymphocytes isolated from the peripheral blood of a healthy donor, pseudonormal HaCaT human keratinocytes, and Balb/c 3T3 murine fibroblasts was investigated. We did not observe significant cytotoxic activity of the tested compounds towards these cells (Figure 4, Table 6). Les-6547 and Les-6557 at a high concentration of 100 µM decreased the viability of lymphocytes from a healthy donor by only 25% compared to control cells. Murine fibroblasts of the Balb/c 3T3 line were more sensitive to the treatment with studied derivatives than human lymphocytes isolated from the peripheral human blood and HaCaT cells, while doxorubicin demonstrated the strongest cytotoxic activity against normal (lymphocytes) and pseudonormal cells (HaCaT, Balb/c 3T3), reducing cell viability by 50% at concentrations lower than 1 µM (Figure 4, Table 6).

Thus, we did not observe the high cytotoxic activity of the tested compounds against normal (human lymphocytes isolated from healthy donors) and pseudonormal cells (HaCaT keratinocytes, Balb/c 3T3 fibroblasts). It is important to note that the cytotoxic activity of Les-6547 and Les-6557 against colorectal cancer cells was observed to be several dozen times higher than against normal and pseudonormal cells. For example, the cytotoxic activity of both agents against the DLD-1 colorectal adenocarcinoma cell line was more than 16 times higher compared with lymphocytes isolated from the peripheral blood of healthy donors (IC_50_ ~6 µM vs. >100 µM, 72 h of exposure), while doxorubicin at doses <1 µM killed 50% of normal lymphocytes, pseudonormal keratinocytes, and fibroblasts. The results indicate the selective action of the newly synthesized juglone-bearing thiopyrano[2,3-d]thiazoles, a crucial characteristic for promising anticancer compounds. This is particularly important because most approved anticancer drugs affect both cancerous and normal cells, which significantly impacts the quality of life of cancer patients.

It is worth noting that the lower IC_50_ value observed in Balb/c 3T3 cells (31.69 µM for Les-6547) raises considerations about potential toxicity. However, since Balb/c 3T3 cells are murine fibroblasts, their response may not fully reflect the selectivity of these compounds in human tissues. Importantly, the IC_50_ values for HaCaT human keratinocytes and human lymphocytes exceed 68 µM and 100 µM, respectively, suggesting a much better selectivity profile in human cells.

While the therapeutic window is relatively narrow compared with some targeted therapies, it is still broader than that of widely used chemotherapeutic agents like doxorubicin, which exhibits significant toxicity to normal cells at submicromolar concentrations.

### 3.4. Thiopyrano[2,3-d]thiazoles Inhibit Proliferation in Colorectal Adenocarcinoma Cells

The anti-proliferative potential of Les-6547 and Les-6557 was analyzed using a clonogenic assay and incorporation of radioactive [^3^H]-thymidine into the DNA of colorectal adenocarcinoma HT-29 and DLD-1 cell lines after 24 h of incubation with various concentrations of the tested agents.

**Figure 5:**
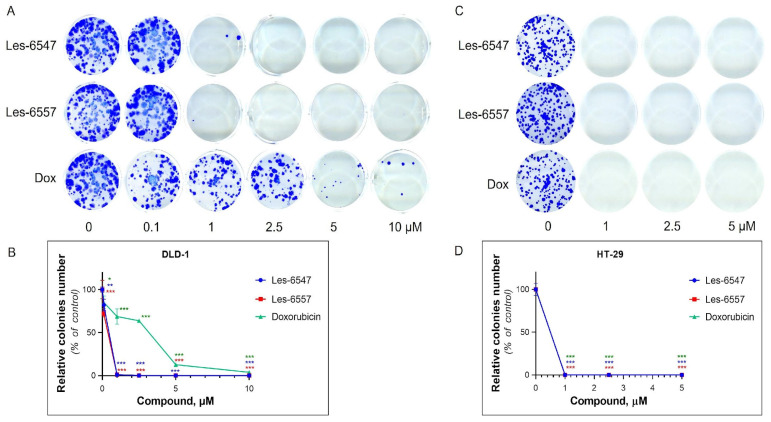
Long-term effect (14 days) of Les-6547, Les-6557, and doxorubicin on the colony-forming ability of DLD-1 and HT-29 colorectal adenocarcinoma cells following 72 h treatment: (**A**,**C**) representative images of formed colonies in cell culture wells; (**B**,**D**) relative number of formed colonies in treated cells (% of control). Data are expressed as a percentage of the control group and presented as the mean value ± SD of three independent experiments (*n* = 3) performed in triplicate. * *p* < 0.05; ** *p* < 0.01; *** *p* < 0.001 compared with control cells.

The colony formation assay evaluated the ability of the cells to survive, proliferate, and form colonies (long-term growth capacity) under the action of the tested agents [52]. Les-6547 and Les-6557 inhibited the formation of colonies in HT-29 and DLD-1 cells (Figure 5, Table 7).

Les-6547 and Les-6557 at 1 µM inhibited the colony formation of HT-29 cells by 100%. In the case of DLD-1 cells, Les-6547 and Les-6557 at 1 µM inhibited colony formation by 99%. Les-6547 and Les-6557 at 2.5 µM completely inhibited colony formation in both studied colorectal cancer cell lines. Doxorubicin demonstrated a similar effect on colony formation in HT-29 cells as Les-6547 and Les-6557. Doxorubicin at 1 µM entirely prevented colony formation of HT-29 cells (100% inhibition). However, it was less effective in inhibiting colony formation in DLD-1 cells, where we observed 3.86% colony growth even after exposure to 10 µM doxorubicin (Figure 5, Table 7).

**Figure 6:**
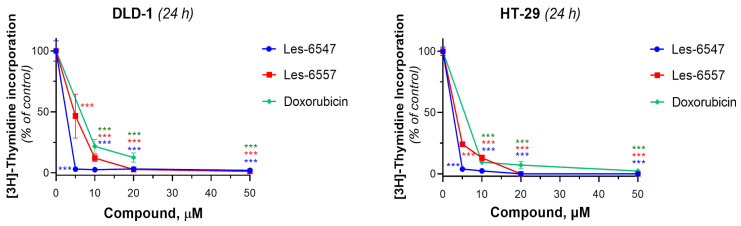
[^3^H]-thymidine incorporation into the DNA of HT-29 colorectal adenocarcinoma cells after 24 h treatment with Les-6547, Les-6557, and doxorubicin (as a reference drug). Results are expressed as a percentage of the control group and presented as the mean ± SD obtained from three independent experiments (*n* = 3) conducted in triplicate. *** *p* < 0.001 compared with the control cells.

Next, we examined the effects of the tested compounds on the proliferative activity of human colorectal adenocarcinoma cells by assessing their ability to influence DNA biosynthesis using the thymidine incorporation assay (Figure 6, Table 8). Les-6547 decreased DNA biosynthesis in DLD-1 cells to 50% at a concentration of 2.19 ± 0.39 µM. The IC_50_ value for Les-6557 in DLD-1 cells was 4.65 ± 0.02 µM. Les-6547 inhibited DNA biosynthesis in HT-29 cells with an IC_50_ of 2.21 ± 0.39 µM, and Les-6557 with an IC_50_ of 2.91 ± 0.38 µM. The IC_50_ for doxorubicin was higher in both the tested colon cancer cell lines: 6.39 ± 0.02 µM in DLD-1 cells and 4.72 ± 0.80 µM in HT-29 cells (Figure 6, Table 8).

Thus, the tested compounds Les-6547 and Les-6557 exhibited pronounced antiproliferative activity against human colorectal carcinoma cells of the HT-29 and DLD-1 lines, as demonstrated by the results of the colony formation assay and the [3H]-thymidine incorporation assay. Both studied derivatives inhibited the survival, proliferative, and colony-forming activity of DLD-1 cells significantly more effectively than the reference drug doxorubicin. Les-6547 and Les-6557 also suppressed the synthesis of newly dividing DNA strands in HT-29 and DLD-1 cells to a greater extent than doxorubicin.

### 3.5. Thiopyrano[2,3-d]thiazoles Induce Apoptosis via Extrinsic and Intrinsic Pathways

**Figure 7:**
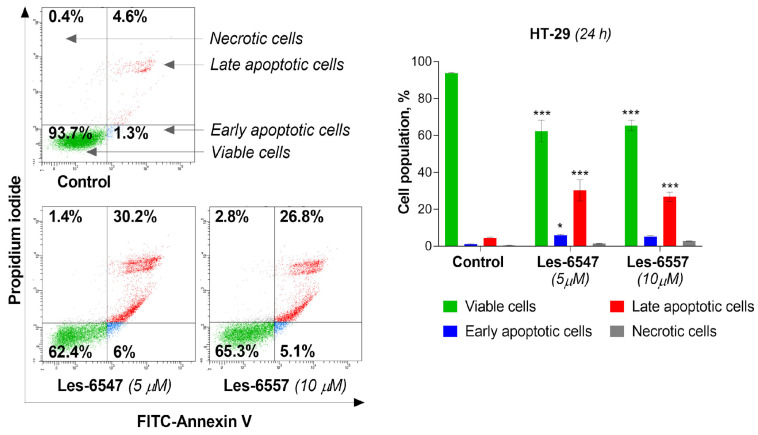
Results of flow cytometric analysis of apoptosis in HT-29 colorectal adenocarcinoma cells using FITC-Annexin V (AV)/propidium iodide (PI) double staining after 24 h of incubation with Les-6547 (5 μM) and Les-6557 (10 μM). The percentage of viable (green), early apoptotic (blue), late apoptotic (red), and necrotic (gray) cells presented as mean ± SD of three separate experiments (*n* = 3) in triplicate. * *p* < 0.05; *** *p* < 0.001 compared with control cells.

Current trends in the design and synthesis of compounds with anticancer activity are mainly focused on creating drugs that can induce the process of apoptosis in cancer cells, leading to cancer cell death [53]. Therefore, we evaluated the proapoptotic activity of the tested compounds: Les-6547 (5 μM) and Les-6557 (10 μM) in HT-29 colon cancer cells after 24 h exposure. Les-6547 proved to be the most active compound (Figure 7), with 36.2% apoptotic cells (sum of early and late apoptotic cells). For the compound Les-6557, 31.9% apoptotic cells were observed. Similar apoptotic cell population values compared with Les-6547 were observed for Les-6557; however, it should be noted that the second compound Les-6557 induced a similar proapoptotic effect at a concentration twice as high (10 μM).

**Figure 8:**
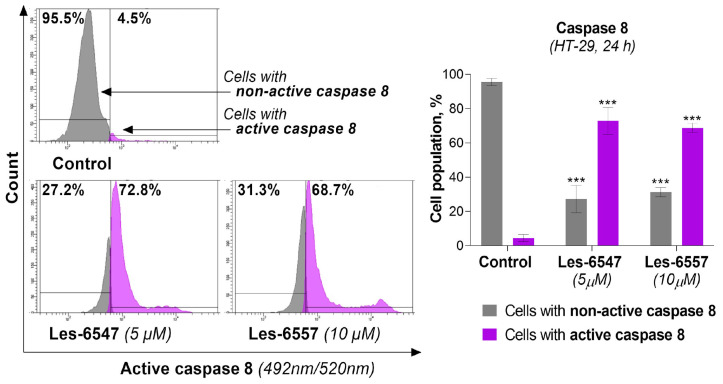
Activity of caspase 8 in HT-29 colorectal adenocarcinoma cells treated for 24 h with Les-6547 (5 μM) and Les-6557 (10 μM), measured via flow cytometry. The percentage of cells with active (violet) and non-active (gray) caspase 8 is presented as mean ± SD of three separate experiments (*n* = 3) conducted in triplicate. *** *p* < 0.001 compared with control cells.

**Figure 9:**
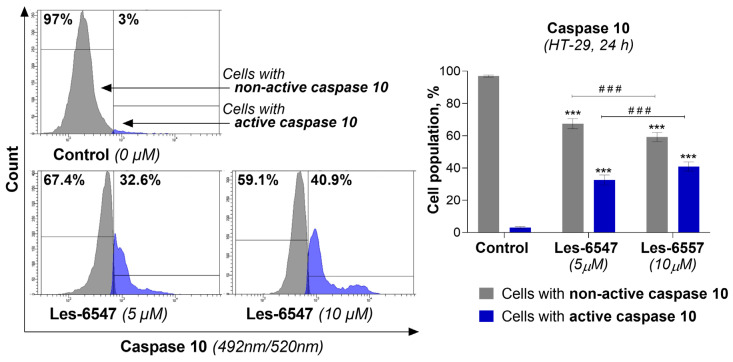
Activity of caspase 10 in HT-29 colorectal adenocarcinoma cells treated for 24 h with Les-6547 (5 μM) and Les-6557 (10 μM) measured via flow cytometry. The percentage of cells with active (blue) and non-active (gray) caspase 10 is presented as a mean ± SD of three separate experiments (*n* = 3) conducted in triplicate. *** *p* < 0.001 compared with control cells, ^###^ *p* < 0.001 difference between compounds.

Because caspases 8 and 10 are key components of the extrinsic apoptosis pathway [54], we examined the effects of the tested compounds, Les-6547 (5 μM) and Les-6557 (10 μM), on their activity in HT-29 colon cancer cells following 24 h exposure. The findings of this research demonstrate that these tested compounds significantly affected the activation of these proteases in the tested cells (Figure 8 and Figure 9). With Les-6547, 72.8% of cells were observed with active caspase 8 and 32.6% of cells with active caspase 10, while with Les-6557, 68.7% and 40.9% were observed, respectively. The overall conclusion emerging from this study is that the obtained data appropriately correlate with the results of the Annexin V and PI double-staining assay (Figure 7), and the tested compounds induced apoptosis mediated by the extrinsic pathway.

**Figure 10:**
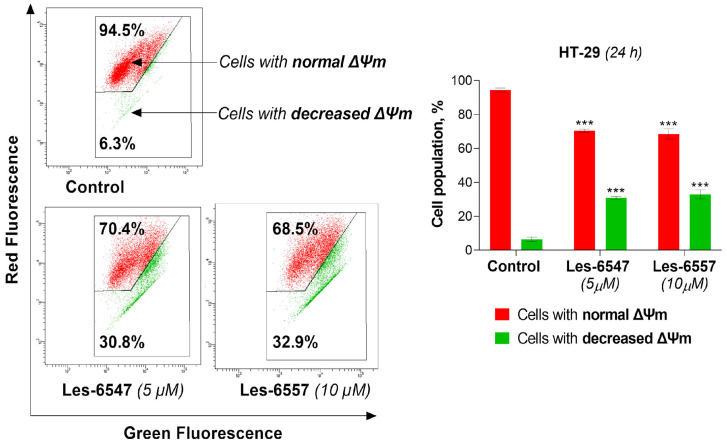
Results of flow cytometry analysis of changes in mitochondrial membrane potential (ΔΨm) in HT-29 colorectal adenocarcinoma cells using staining with JC-1 after 24 h exposure with Les-6547 (5 μM) and Les-6557 (10 μM). Percentages of cells with normal ΔΨm (red fluorescence of JC-1) and decreased ΔΨm (green fluorescence of JC-1) presented as mean ± SD of three separate experiments (*n* = 3) conducted in triplicate. *** *p* < 0.001 compared with the control cells.

In the next step, we aimed to investigate whether the examined derivatives could induce apoptosis via the mitochondrial-dependent (intrinsic) pathway. Therefore, we evaluated the impact of the examined compounds, Les-6547 (5 μM) and Les-6557 (10 μM), on the mitochondrial membrane potential (ΔΨm) in HT-29 colon cancer cells following 24 h exposure. The results of the ΔΨm assay clearly showed that the tested compounds significantly decreased the mitochondrial membrane potential (Figure 10). Les-6547 decreased mitochondrial potential in 30.8% of cells, while Les-6557 reduced it in 32.9% of cells.

Taking into account that caspase 9 is a key protein in intrinsic apoptotic pathway 36, we analyzed its activity in HT-29 colon cancer cells after treatment with Les-6547 (5 μM) and Les-6557 (10 μM) following 24 h exposure.

**Figure 11:**
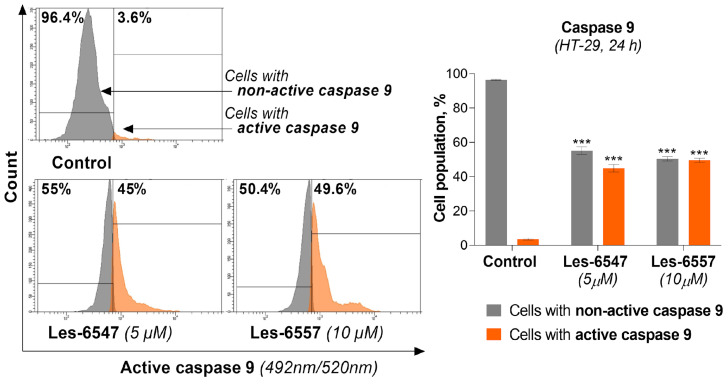
Activity of caspase 9 in HT-29 colorectal adenocarcinoma cells treated for 24 h with Les-6547 (5 μM) and Les-6557 (10 μM) measured with flow cytometry. The percentage of cells with active (orange) and non-active (gray) caspase 9 is presented as mean ± SD of three separate experiments (*n* = 3) conducted in triplicate. *** *p* < 0.001 compared with control cells.

The tested compounds caused an increase in the active form of this protease in the examined cell lines (Figure 11). For compound Les-6547, we observed 45.0% of cells with active caspase 9, while for Les-6557 this was the case for 49.6% of cells. These results indicating decreased mitochondrial membrane potential and an increase in the active form of caspase 9 in HT-29 colon cancer cells confirm the results of previous studies (AV/PI) and are consistent with those findings, suggesting that these compounds may induce apoptosis via the intrinsic pathway.

**Figure 12:**
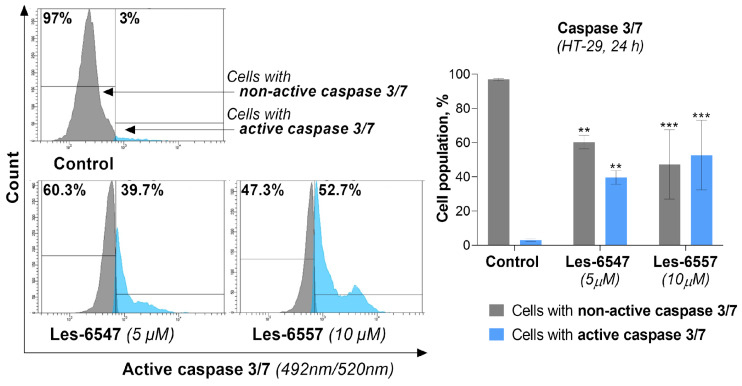
Activity of caspase 3/7 in HT-29 colorectal adenocarcinoma cells treated for 24 h with Les-6547 (5 μM) and Les-6557 (10 μM), measured via flow cytometry. The percentage of cells with active (blue) and non-active (gray) caspase 3/7 is presented as mean ± SD of three separate experiments (*n* = 3) carried out in triplicate. ** *p* < 0.01; *** *p* < 0.001 compared with control cells.

To confirm previous studies, the final measurements performed involving the molecular mechanism of the apoptosis process assessed caspase 3/7 activity in HT-29 colon cancer cells treated with the tested compounds following 24 h exposure. For Les-6547, we observed that 39.7% of cells had active caspase 3/7, while for Les-6557, this was 52.7% of cells (Figure 12).

Comparison with previous studies allows us to see the correlation of the above results with other data obtained from the analysis of apoptosis induction and the proteins involved in it, proving that the studied derivatives trigger the apoptosis process in HT-29 colon cancer cells via two pathways (i.e., extrinsic and intrinsic pathways).

### 3.6. Thiopyrano[2,3-d]thiazoles Stimulate ROS Generation in Colorectal Cancer Cells

Recent scientific reports have demonstrated that reactive oxygen species (ROS) are key factors involved in the induction of cancerous transformation [55]. ROS play a dual role in cancer cell metabolism depending on their levels. Low or moderate ROS levels promote cancer cell proliferation, migration, invasion, and angiogenesis. In contrast, high levels of ROS induce programmed cell death via activation of extrinsic or intrinsic apoptotic pathways [56]. Since cancer cells are known to exhibit higher basal levels of ROS compared with normal cells, they are more susceptible to oxidative stress. Therefore, targeting cancer cells with compounds that further elevate ROS levels to a threshold that triggers cell death represents a promising therapeutic approach for cancer treatment [57].

**Figure 13:**
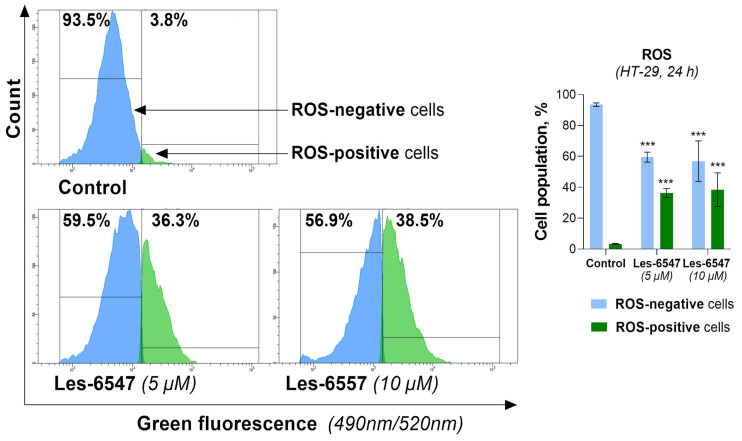
Levels of total intracellular ROS in HT-29 colorectal adenocarcinoma cells after 24 h of exposure to Les-6547 (5 μM) and Les-6557 (10 μM), measured via flow cytometry. Percentages of ROS positive (green) and negative (blue) cells presented as mean ± SD of three independent experiments (*n* = 3) conducted in triplicate. *** *p* < 0.001 compared with control cells.

**Figure 14:**
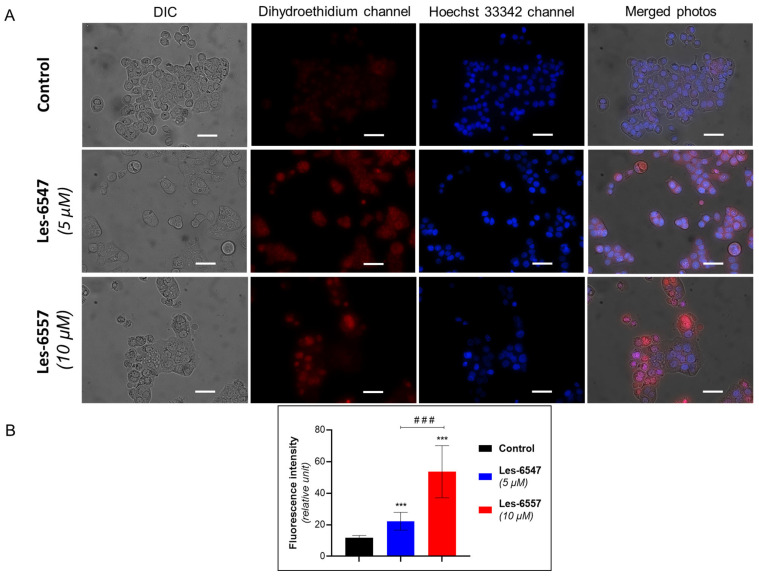
Fluorescent microscopy data of HT-29 cells following 24 h treatment with Les-6547 (5 μM) and Les-6557 (10 μM): (**A**) representative fluorescence images of control and treated cells; (**B**) fluorescence intensity of DHE in control and treated cells (expressed in relative units). Cells were stained with DHE to assess ROS levels and Hoechst 33342 for nuclear visualization. *** *p* < 0.001 compared with control cells, ^###^ *p* < 0.001 difference between compounds. Scale bar = 20 µm.

Based on the dual role of ROS, we evaluated the effect of the newly synthesized agents on oxidative stress in HT-29 cells after 24 h of incubation (Figure 13 and Figure 14). Les-6547 and Les-6557 increased the number of ROS-positive cells in the HT-29 population by approximately 10-fold compared with the control cells (36.3% and 38.5% vs. 3.8%, respectively, Figure 13). The higher fluorescence of dihydroethidium (DHE) confirmed the production of ROS in treated HT-29 cells (Figure 14). More prominent changes in DHE fluorescence were observed under the effect of Les-6557. DHE can easily cross the cell membrane and undergo oxidation by cellular superoxide (O2•−), producing red fluorescent compounds. These products include ethidium, which is formed through general redox reactions, and 2-hydroxyethidium (2-OH-E+), a specific byproduct resulting from direct interaction with superoxide [58]. It can be assumes that the studied derivatives elevated the levels of superoxide and/or affected the nonspecific redox processes in the tested cells.

The obtained results are consistent with the observations of the tested derivatives’ induction of apoptosis in HT-29 cells. Given the well-documented role of ROS in triggering apoptotic pathways, it is plausible that elevated ROS levels such as those observed in our study may contribute to mitochondrial dysfunction, which could further promote apoptosis and enhance the cytotoxic effects in colorectal adenocarcinoma cells. However, additional studies are required to confirm this relationship.

### 3.7. Thiopyrano[2,3-d]thiazoles Induce Cell Cycle Arrest in Colorectal Cancer Cells

The cell cycle is an ordered sequence of events that culminates in cell division, making it a critical process for cellular proliferation. This process is often hyperactivated in cancer cells, contributing to uncontrolled growth, increased migration, invasion, and metastasis [59]. Furthermore, it is known that cell cycle arrest can lead to programmed cell death. So, compounds that promote cell cycle arrest not only inhibit the proliferation of cancer cells but also lead to their death, presenting an effective strategy for cancer treatment [60].

**Figure 15:**
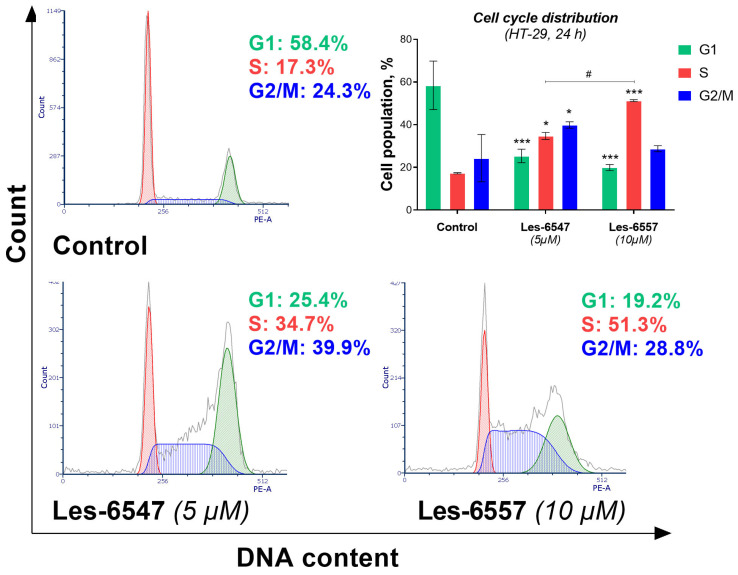
Flow cytometry analysis of cell cycle distribution in HT-29 colorectal adenocarcinoma cells treated for 24 h with Les-6547 (5 μM) and Les-6557 (10 μM). Percentages of cells in the G1 (green), S (red), and G2/M (blue) phases of the cell cycle are presented as mean ± SD obtained from three independent experiments (*n* = 3) conducted in triplicate. * *p* < 0.05; *** *p* < 0.001 compared to the control cells; ^#^ *p* < 0.05 difference between compounds.

To determine whether the anticancer activity of the newly synthesized thiopyrano[2,3-d]thiazoles is linked to cell cycle regulation, we assessed the distribution of phases in HT-29 colorectal cancer cells (Figure 15). The results indicate that Les-6547 influenced the cell cycle by inhibiting progression through the S and G2/M phases. Treatment of HT-29 cells with Les-6547 resulted in an increase in the G2/M phase cell population from 24.3% to 39.9%, compared with the control group. A similar increase was observed in the S phase, where the population increased from 17.3% to 34.7%. Les-6557 induced cell cycle arrest in the S phase, as indicated by an increase in the cell population from 14.3% in the control group to 51.3% in the treated group (Figure 15).

The obtained results were consistent with the findings from the [^3^H]-thymidine incorporation assay, which indicated that the thiopyrano[2,3-d]thiazoles derivatives inhibited proliferation, as well as with the FITC-Annexin V/propidium iodide analysis and caspase activity assays, which demonstrated the pro-apoptotic activity of the tested compounds. This dual effect of inducing apoptosis and halting cell cycle progression may be particularly beneficial in targeting the aggressive and fast-dividing nature of colorectal cancer cells.

The promising results obtained with Les-6547 and Les-6557 underscore their potential as candidates for further development for colorectal cancer therapy. However, additional studies are necessary to thoroughly investigate their in vivo efficacy and safety profile and explore potential synergistic effects with existing chemotherapeutic agents. Moreover, given the complex nature of colorectal cancer and its tendency to develop resistance to conventional treatments, the dual apoptotic activation observed in this study suggests that Les-6547 and Les-6557 could be valuable in overcoming such resistance. Exploring their effects on different molecular subtypes of colorectal cancer could further clarify their therapeutic scope and help identify patient populations that might benefit the most from such treatment.

## 4. Discussion

Colorectal cancer is one of the most common and deadly types of cancer worldwide [61]. Despite significant advancements in its treatment, traditional therapeutic methods such as chemotherapy and radiotherapy often show limited effectiveness, particularly at advanced stages and in cases of drug resistance [62]. This highlights the urgent need for new, more effective, and less toxic treatments that can address these challenges. In this study, we evaluated the anticancer potential of two novel juglone-bearing thiopyrano[2,3-d]thiazoles, Les-6547 and Les-6557, against human colorectal adenocarcinoma cell lines (HT-29 and DLD-1). Our findings suggest that these compounds exhibited potent anti-proliferative and pro-apoptotic activity, positioning them as promising candidates for colorectal cancer therapy.

The in silico ADMET predictions for Les-6547 and Les-6557 indicate that both compounds possess favorable drug-like properties, such as good intestinal absorption, high plasma protein binding, and satisfactory excretion profiles, making them promising candidates for therapeutic development [49]. These properties suggest that these compounds may have the potential for effective oral administration and appropriate bioavailability, which are essential for their further investigation as anticancer agents.

The selective cytotoxicity of anticancer agents is crucial for their potential therapeutic application. Our findings demonstrated that Les-6547 and Les-6557 exhibited significantly higher cytotoxic activity against colorectal cancer cell lines than against normal and pseudonormal cells. Notably, their cytotoxicity towards DLD-1 cells was over 16 times greater than that towards human lymphocytes isolated from healthy donors (IC_50_ ~6 µM vs. >100 µM, 72 h of exposure). In contrast, doxorubicin, a widely used chemotherapeutic agent, induces significant cytotoxicity in normal cells at submicromolar concentrations, highlighting the potential advantage of Les-6547 and Les-6557 in terms of selectivity.

However, the observed IC50 value of 31.69 µM for Les-6547 in Balb/c 3T3 murine fibroblasts raises considerations regarding potential off-target toxicity. It is important to emphasize that Balb/c 3T3 cells are of murine origin, and their response may not fully represent the selectivity of these compounds in human tissues. In contrast, human-derived pseudonormal cells (HaCaT keratinocytes) exhibited significantly higher IC_50_ values (89.64 µM for Les-6547 and 68.37 µM for Les-6557), while human lymphocytes demonstrated an even greater resistance (>100 µM for both compounds). These findings suggest a more favorable selectivity profile of the tested compounds towards human colorectal cancer cells.

Although the therapeutic window appears relatively narrow compared with some targeted therapies, it remains broader than that of conventional chemotherapeutic agents, which often exhibit indiscriminate cytotoxicity. Further in vivo studies are necessary in order to evaluate the pharmacokinetic properties, systemic toxicity, and overall therapeutic index of these compounds in a more physiologically relevant setting.

Colony formation analysis demonstrated that Les-6547 and Les-6557 at a concentration of 1 µM effectively inhibited colony formation in both HT-29 and DLD-1 cells. These results are consistent with the thymidine incorporation assay, which further confirmed the compounds’ ability to block DNA biosynthesis in both cancer cell lines. The data suggest that Les-6547 and Les-6557 can significantly inhibit the proliferative capacity of colorectal cancer cells, a key factor in tumor growth and metastasis [63].

Interestingly, Les-6547 and Les-6557 were more effective than doxorubicin in inhibiting colony formation in DLD-1 cells, highlighting their superior anti-proliferative potential in certain colorectal cancer subtypes. This finding suggests that Les-6547 and Les-6557 are not only effective in inhibiting cancer cell growth but may also exhibit selectivity in targeting aggressive colorectal cancer cells. The higher efficacy of these compounds in inhibiting cell proliferation, combined with their lower IC_50_ values, positions them as promising candidates for therapeutic intervention, particularly for patients who may exhibit resistance to traditional chemotherapeutic agents such as doxorubicin.

Our results also demonstrated that Les-6547 and Les-6557 induced cell cycle arrest, which further supports their anti-proliferative effects [60,64]. Les-6547 caused an increase in the proportion of cells in the G2/M and S phases, suggesting that it inhibited cell cycle progression particularly at the transition between the S and G2/M phases. Les-6557, on the other hand, induced a more prominent arrest in the S phase. Cell cycle arrest is a well-known mechanism of action for anticancer agents, as it prevents cells from progressing through critical phases of division, ultimately leading to apoptosis [65]. These findings are consistent with the results from the colony formation and DNA biosynthesis assays, which indicated that Les-6547 and Les-6557 effectively inhibited cell proliferation.

An important hallmark of cancer therapy is the ability of compounds to induce programmed cell death, or apoptosis [65]. Our study showed that both Les-6547 and Les-6557 acted as potent inducers of apoptosis in HT-29 cells, triggering both extrinsic and intrinsic apoptotic pathways.

In the extrinsic pathway, the activation of caspases 8 and 10 was significantly increased by both compounds. This marked elevation in caspase 8 and caspase 10 activity in treated cells suggests that Les-6547 and Les-6557 effectively activated the death receptor pathway, leading to apoptosis. These findings were supported by the results of the Annexin V/PI staining assay, which revealed a significant proportion of apoptotic cells in response to the treatment. This indicates that the compounds may target cell surface death receptors, initiating the extrinsic apoptosis cascade.

In addition to the extrinsic pathway, the compounds also activated the intrinsic apoptosis pathway, as evidenced by a decrease in mitochondrial membrane potential (ΔΨm) and increased activity of caspase 9 [66,67]. Both Les-6547 and Les-6557 caused notable reductions in ΔΨm, suggesting mitochondrial dysfunction, relating to a critical step in the intrinsic apoptotic pathway. The subsequent activation of caspase 9, a key protease in the intrinsic pathway, further confirms the involvement of this pathway in the induction of apoptosis [68]. Furthermore, the activation of effector caspases 3/7 further supports the pro-apoptotic effects of these compounds, as these caspases are critical for the execution phase of apoptosis [67].

The dual activation of extrinsic and intrinsic apoptotic pathways provides strong evidence for the potency of Les-6547 and Les-6557 in promoting cancer cell death. This dual mechanism of action could be particularly beneficial for overcoming the ability of cancer cells to evade apoptosis, a common feature of many malignancies, including colorectal cancer.

An important aspect of the anticancer properties of Les-6547 and Les-6557 is their ability to induce oxidative stress through the generation of reactive oxygen species (ROS). Cancer cells, due to their higher basal levels of ROS compared with normal cells, are particularly sensitive to further ROS elevation, which can overwhelm cellular antioxidant defenses and lead to cell death [57,69].

Our findings showed that both Les-6547 and Les-6557 significantly increased the number of ROS-positive cells in HT-29 cells, with approximately 10-fold more cells exhibiting ROS positivity compared with the control cells. The elevated ROS levels observed in treated cells were confirmed by increased fluorescence of DHE, suggesting that these compounds promote the generation of superoxide and other reactive species, leading to oxidative damage. ROS-induced oxidative stress may contribute to mitochondrial dysfunction, which, in turn, promotes the activation of apoptotic pathways [70]. The correlation between ROS production and the observed apoptotic effects in HT-29 cells supports the notion that ROS play a crucial role in mediating the cytotoxic effects of Les-6547 and Les-6557.

Since cancer cells are already in a state of elevated oxidative stress, targeting this vulnerability with compounds like Les-6547 and Les-6557 could prove an effective therapeutic strategy [71,72]. By pushing ROS levels beyond the threshold of cellular tolerance, these compounds may induce irreversible damage, leading to cell death. Moreover, ROS generation in combination with apoptosis induction suggests that these compounds could act synergistically to maximize their anticancer effects.

The results suggest that the studied thiopyrano[2,3-d]thiazole derivatives may serve as promising CDK2, JAK2, and MAPK8 inhibitors. The higher docking scores compared with the co-crystallized ligands with experimentally confirmed activity indicate the strong binding affinity of Les-6547 and Les-6557 to these protein targets. This may explain their cytotoxic and proapoptotic activity, as CDK2, JAK2, and MAPK8 are key regulators of cell growth, proliferation, and apoptosis. Inhibition of these kinases can lead to cell cycle arrest, disruption of survival signaling pathways, and activation of programmed cell death. Further deeper studies, such as molecular dynamics simulations and experimental validation of interactions, are necessary to assess the stability and biological activity of these compounds.

In summary, Les-6547 and Les-6557 demonstrate strong anticancer activity through multiple mechanisms, including inhibition of cell proliferation, induction of ROS generation, cell cycle arrest, and activation of both extrinsic and intrinsic apoptotic pathways. These compounds exhibited a significant capacity to target colorectal cancer cells, particularly HT-29 and DLD-1, with greater efficacy compared with conventional chemotherapeutic agents like doxorubicin.

Numerous recent studies have explored the potential of thiazole derivatives as anticancer agents. Notably, our team recently investigated the anticancer activity of a series of newly synthesized thiopyrano[2,3-d]thiazoles. The most active compound (3.10) induced both intrinsic and extrinsic apoptosis, caused G2/M phase arrest, and suppressed DNA and RNA synthesis in tumor cells [27]. Al-Salmi et al. demonstrated that thiazole derivatives exhibited significant cytotoxicity against MCF-7 and HepG2 cells, with one compound showing promising effects by inducing cell cycle arrest and apoptosis in MCF-7 through VEGFR-2 inhibition [73]. A recent study by Shosha et al. (2025) described a compound that exhibited strong anticancer activity against HepG2 and MCF-7 cells, binding with DNA and topoisomerase II enzyme, highlighting its potential through multiple molecular interactions [74].

Our study, along with several recent investigations, confirms the significant anticancer potential of thiazole derivatives, making them promising candidates for further research. A review of Les-6547 and Les-6557’s safety profiles and therapeutic potential in vivo should be undertaken in future research. In addition, organ-on-chip platforms that replicate the 3D microenvironment of colorectal tumors may be used for testing these compounds. Organ-on-chip technology acts as a bridge between in vitro studies and animal models. Utilizing these systems may aid in refining drug formulations and personalized treatment strategies prior to clinical testing.

## 5. Conclusions

The juglone-bearing thiopirano[2,3-d]thiazoles Les-6547 and Les-6557 exhibited pronounced cytotoxic activity against human colorectal cancer HT-29 and DLD-1 cells, showing significantly higher efficacy compared with pseudo-normal cells (Balb/c 3T3 fibroblasts, HaCaT keratinocytes) and lymphocytes from the blood of a healthy donor.

The mechanism of action of the studied heterocyclic derivatives involved both intrinsic and extrinsic apoptotic pathways, characterized by a decrease in mitochondrial membrane potential and increased activity of initiator caspases 9, 8, and 10. These compounds also activated effector caspases 3/7 in HT-29 colorectal adenocarcinoma cells. Additionally, Les-6547 and Les-6557 led to a significant increase in the number of ROS-positive cells and caused cell cycle arrest, elevating the proportion of HT-29 cells in the S and G2/M phases. These results indicate the promising potential of these newly synthesized juglone-bearing thiopyrano[2,3-d]thiazoles, Les-6547 and Les-6557, for use in colorectal cancer treatment. This study reveals the promising potential of Les-6547 and Les-6557 as colorectal cancer treatment candidates and provides a strong foundation for further in-depth investigation of their molecular mechanisms in vitro and therapeutic potential in vivo.

## Figures and Tables

**Table 1:** Predicted ADMET parameters for derivatives Les-6547 and Les-6557 calculated using ADMETlab 3.0 (data presented in the format: predicted value/probability or value).

Compounds/Parameters	Physicochemical Properties						Medicinal Chemistry			
	Molecular Weight, ≤500	nHA ≤ 10	nHD ≤ 5	TPSA ≤ 140	Log S	Log P ≤ 5	Fsp3	Lipinski Rule	Pfizer Rule	GSK Rule
Les-6547	442.97	6	3	107.46	−5.956	3.920	0.05	Accepted	Accepted	Rejected
Les-6557	426.97	5	2	87.23	−6.018	3.893	0.05	Accepted	Accepted	Rejected

nHA—number of hydrogen bond acceptors; nHD—number of hydrogen bond donors; TPSA—topological polar surface area; Log S—water solubility; Log P—lipophilicity parameter; Fsp3—fraction of sp3 atoms.

**Table 2:** Predicted ADMET parameters for derivatives Les-6547 and Les-6557 calculated using ADMETlab 3.0 (data presented in the format: predicted value/probability or value).

Compounds/Parameters	Absorption					Distribution			Metabolism	
	MDCK ^1^ Permeability, cm/s	Caco-2 Permeability ^2^	P-GP ^2^ inhibitor	P-GP ^2^ substrate	HIA ^3^	PPB ^4^	BBB ^5^	Fu ^6^	CYP2D6 ^7^ inh/sub	CYP3A4 ^7^ inh/sub
Les-6547	0/excellent	−5.513/poor	−/medium	−−−/excellent	−/medium	99.5%/poor	−−−/excellent	0.3%/low	++/−−−	+++/+++
Les-6557	0/excellent	−5.341/poor	++/ poor	−−−/excellent	−/medium	99.5%/poor	−−−/excellent	0.2%/low	−−−/−	+++/+++

^1^ Madin−Darby canine kidney cells; ^2^ P-glycoprotein; ^3^ Human intestinal absorption; ^4^ Plasma protein binding; ^5^ Blood–brain barrier; ^6^ Unbound fraction in plasma; ^7^ Cytochrome, inh/sub—inhibitor/substrate.

**Table 3:** Predicted ADMET parameters for derivatives Les-6547 and Les-6557 calculated using ADMETlab 3.0 (data presented in the format: predicted value/probability or value).

Compounds/Parameters	Excretion		Toxicity		Tox21 Pathway					
	CL ^1^, ml/min/kg	T_1/2_^2^	H-HT ^3^	AMES Toxicity ^4^	AR ^5^	AhR ^6^	Aromatase ^7^	ER ^8^	PPARγ ^9^	ARE ^10^
Les-6547	2.301/excellent	0.942 excellent	0.690 medium	0.545 medium	−−−/excellent	+++/poor	+/ medium	+/medium	−−−/excellent	+++/poor
Les-6557	1.532/excellent	0.942 excellent	0.517 medium	0.439 medium	−−−/excellent	++/poor	+/ medium	+/medium	−−−/excellent	+++/poor

^1^ CL—clearance of compound; ^2^ T1/2—half-life of compound; ^3^ H-HT—human hepatotoxicity; ^4^ AMES Toxicity—the most widely used assay for testing the mutagenicity of compounds; ^5^ AR—androgen receptor, the output value is the probability of being agonist to the AR receptor; ^6^ AhR—aryl hydrocarbon receptor, the output value is the probability of being an activator of the AhR signaling pathway; ^7^ aromatase—the output value is the probability of being of aromatase inhibitor; ^8^ ER—estrogen receptor, the output value is the probability of being agonist to the ER receptor; ^9^ PPARγ—peroxisome proliferator-activated receptors gamma, the output value is the probability of being agonist to the PPARγ; ^10^ AER—antioxidant response element signaling pathway, the output value is the probability of being an activator of the ARE signaling pathway.

**Table 4:** Shortlist of protein targets predicted by SwissTargetPrediction and selected for molecular docking studies.

Predicted Target	*n*	Vina Scores, kkal/mol, Mean ± SD				
		CCLs	Les-6547-R	Les-6547-S	Les-6557-R	Les-6557-S
Cyclin-dependent kinase 2 (CDK2)	202	−7.57 ± 2.16	−8.26 ± 2.44	−8.47 ± 1.77	−8.77 ± 2.26	−8.66 ± 6.40
Cyclin-dependent kinase 4 (CDK4)	5	−9.74 ± 0.68	−8.07 ± 0.90	−8.77 ± 1.92	−8.08 ± 0.88	−9.20 ± 0.95
Tyrosine-protein kinase JAK2	117	−8.20 ± 1.64	−9.14 ± 0.59	−9.54 ± 0.74	−9.27 ± 0.58	−9.64 ± 0.71
Mitogen-activated protein kinase 8 (MAPK8)	17	−6.91 ± 1.06	−8.90 ± 0.78	−8.70 ± 0.92	−8.97 ± 0.74	−8.84 ± 0.66
MAP kinase signal-integrating kinase 2 (MKNK2)	10	−7.19 ± 2.20	−9.05 ± 0.13	−8.75 ± 1.00	−9.33 ± 0.60	−8.90 ± 0.89
Matrix metalloproteinase 9 (MMP9)	11	−7.80 ± 2.21	−9.08 ± 0.93	−8.36 ± 1.05	−8.65 ± 0.86	−8.29 ± 1.46

*n*—number of available PDB structures of the target in different conformations bound to co-crystallized drug-like ligands (CCLs), obtained from the RCSB PDB database and included in the current analysis. Les-6547-R, Les-6547-S, Les-6557-R, Les-6557-S—enantiomers of Les-6547 and Les-6557, respectively. Each value in the CCLs column represents the average Vina score of the different CCLs, each bound to a different conformation of predicted target, evaluated by AutoDock Vina in score-only mode without performing docking. The remaining four columns contain average Vina docking scores of Les-6547 and Les-6557 enantiomers docked to the same conformations as the targets that bind CCLs. For each of *n* target conformations, the most favorable docking score was selected, and the scores were averaged. The CCLs were removed from the protein structures as the latter were prepared for docking. The scores for Les-6547 and Les-6557 enantiomers were comparable to the scores for CCLs and surpassed them in cases of CDK2, JAK2, and MAPK8, with a similar tendency observed for the other shortlisted targets, except CDK4.

**Table 5:** IC_50_ values (µM) of studied compounds for human colorectal adenocarcinoma cells based on MTT test data (24, 48, 72 h of incubation, M ± SD, *n* = 3).

Cell line	Time, h	Les-6547	Les-6557	Doxorubicin
HT-29	24	5.72 ± 0.41	10.41 ± 0.23	17.03 ± 0.88
	48	4.29 ± 0.50	6.54 ± 0.07	1.55 ± 0.14
	72	4.44 ± 0.35	9.78 ± 0.03	2.11 ± 0.06
DLD-1	24	5.14 ± 0.81	16.85 ± 3.32	65.34 ± 6.08
	48	5.17 ± 0.58	1.94 ± 0.05	2.01 ± 0.09
	72	6.62 ± 0.07	5.19 ± 1.14	0.76 ± 0.10

**Table 6:** The IC_50_ values (µM) of studied compounds for normal and pseudonormal cells based on MTT test data (72 h of incubation, M ± SD, *n* = 3).

Cells	Les-6547	Les-6557	Doxorubicin
Human blood lymphocytes	>100	>100	0.71 ± 0.11
HaCaT (human keratinocytes)	89.64 ± 0.84	68.37 ± 0.57	0.90 ± 0.10
Balb/c 3T3 (mouse fibroblasts)	31.69 ± 0.41	40.26 ± 0.35	0.56 ± 0.11

**Table 7:** The IC_50_ values (µM) of studied compounds for colorectal adenocarcinoma cells based on clonogenic assay data (14 days of incubation, M ± SD, *n* = 3).

Cell Line	Les-6547	Les-6557	Doxorubicin
DLD-1	0.40 ± 0.06	0.33 ± 0.05	3.23 ± 0.05
HT-29	~0.5	~0.5	~0.5

**Table 8:** The IC_50_ values (µM) of studied compounds for colorectal adenocarcinoma cells based on [^3^H]-thymidine incorporation assay data (24 h of incubation, M ± SD, *n* = 3).

Cell Line	Les-6547	Les-6557	Doxorubicin
DLD-1	2.19 ± 0.39	4.65 ± 0.02	6.39 ± 0.02
HT-29	2.21 ± 0.39	2.91 ± 0.38	4.72 ± 0.80

## Data Availability

Data are contained within the article.

## Supplementary Materials

The following supporting information can be downloaded at: https://www.mdpi.com/article/10.3390/cells14060465/s1, Table S1. The output of SwissTargetPrediction for Les-6557. Explanation for Probability and Known actives (3D/2D) columns can be found on the SwissTargetPrediction website (http://www.swisstargetprediction.ch/faq.php (accessed on 14 March 2025)). Table S2. Individual AutoDockVina scores for the ligands docked to the predicted target protein CDK2. Table S3. Individual AutoDockVina scores for the ligands docked to the predicted target protein CDK4. Table S4. Individual AutoDockVina scores for the ligands docked to the predicted target protein JAK2. Table S5. Individual AutoDockVina scores for the ligands docked to the predicted target protein MAPK8. Table S6. Individual AutoDockVina scores for the ligands docked to the predicted target protein MKNK2. Table S7. Individual AutoDockVina scores for the ligands docked to the predicted target protein MMP9.
