# Supplementary material for: Juglone-Bearing Thiopyrano[2,3-d]thiazoles Induce Apoptosis in Colorectal Adenocarcinoma Cells
Source: Cells. 2025 Mar 20;14(6):465. doi: 10.3390/cells14060465 (PMC11941218; doi:10.3390/cells14060465)
Supplement: Supplementary file 1 [file cells-14-00465-s001.zip › cells-3515381-supplementary.pdf]

cells

Supplementary data

Juglone-bearing thiopyrano[2,3-d]thiazoles induce apoptosis in colorectal adenocarcinoma cells

Yuliia Kozak [1], Nataliya Finiuk [1], Robert Czarnomysy [3], Agnieszka Gornowicz [2], Roman Pinyazhko [4], Andrii Lozynskyi [5], Serhii Holota [5], Olga Klyuchivska [1], Andriy Karkhut [6], Svyatoslav Polovkovych [6], Mykola Klishch [1], Rostyslav Stoika [1], Roman Lesyk [5,7], Krzysztof Bielawski [3], Anna Bielawska [2]

[1] Department of Regulation of Cell Proliferation and Apoptosis, Institute of Cell Biology of National Academy of Sciences of Ukraine, Drahomanov 14/16, 79005 Lviv, Ukraine; nataliyafiniuk@gmail.com (N.F.); zorepad1775@gmail.com (O.K.); mykola.klishch1707@gmail.com (M.K.); stoika.rostyslav@gmail.com (R.S.)
[2] Department of Biotechnology, Faculty of Pharmacy, Medical University of Bialystok, Kilinskiego 1, 15-089 Białystok, Poland; agnieszka.gornowicz@umb.edu.pl (A.G.); anna.bielawska@umb.edu.pl (A.B.)
[3] Department of Synthesis and Technology of Drugs, Faculty of Pharmacy, Medical University of Bialystok, Kilinskiego 1, 15-089 Białystok, Poland; robert.czarnomysy@umb.edu.pl (R.C.); krzysztof.bielawski@umb.edu.pl (K.B.)
[4] Department of Normal Physiology, Organic and Bioorganic Chemistry, Danylo Halytsky Lviv National Medical University, Pekarska 69, 79010 Lviv, Ukraine; pinyazhkoroman@gmail.com (R.P.)
[5] Department of Pharmaceutical, Organic and Bioorganic Chemistry, Danylo Halytsky Lviv National Medical University, Pekarska 69, 79010 Lviv, Ukraine; golota_serg@yahoo.com (S.H.); lozynskyiandrii@gmail.com (A.L.); dr_r_lesyk@org.lviv.net (R.L.)
[6] Department of Technology of Biologically Active Substances, Pharmacy and Biotechnology, Lviv Polytecnic National University, Bandera 12, Lviv, 79013, Ukraine; andrew.karkhut@gmail.com (A.K.); spolovkovych@ukr.net (S.P.)
[7] Department of Biotechnology and Cell Biology, Medical College, University of Information Technology and Management in Rzeszów, Sucharskiego 2, 35-225 Rzeszów, Poland
* Correspondence: juliana.kozzak@gmail.com; Tel.: +380-322612287
† These authors contributed equally to this work.

Academic Editor: Firstname Lastname

Received: date
Revised: date
Accepted: date
Published: date

Citation: To be added by editorial staff during production.

Copyright: © 2025 by the authors. Submitted for possible open access publication under the terms and conditions of the Creative Commons Attribution (CC BY) license (https://creativecommons.org/licenses/by/4.0/).

Table S1. The output of SwissTargetPrediction for Les-6557. Explanation for Probability and Known actives (3D/2D) columns can be found on the SwissTargetPrediction website (http://www.swisstargetprediction.ch/faq.php)

Target	Common name	Uniprot ID	ChEMBL ID	Target Class	Probability	Known actives (3D/2D)
Matrix metalloproteinase 3	MMP3	P08254	CHEMBL283	Protease	0,1157366748	263 / 0
Matrix metalloproteinase 8	MMP8	P22894	CHEMBL4588	Protease	0,1157366748	179 / 0
Bone morphogenetic protein 1	BMP1	P13497	CHEMBL3898	Protease	0,1157366748	114 / 0
Inosine-5'-monophosphate dehydrogenase 1	IMPDH1	P20839	CHEMBL1822	Oxidoreductase	0,1157366748	19 / 0
Inosine-5'-monophosphate dehydrogenase 2	IMPDH2	P12268	CHEMBL2002	Oxidoreductase	0,1157366748	46 / 0
c-Jun N-terminal kinase 1	MAPK8	P45983	CHEMBL2276	Kinase	0,1157366748	45 / 0
Matrix metalloproteinase 13	MMP13	P45452	CHEMBL280	Protease	0,1157366748	282 / 0
Matrix metalloproteinase 9	MMP9	P14780	CHEMBL321	Protease	0,1157366748	442 / 0
Matrix metalloproteinase 1	MMP1	P03956	CHEMBL332	Protease	0,1157366748	491 / 0
Heat shock protein HSP 90-alpha	HSP90AA1	P07900	CHEMBL3880	Other cytosolic protein	0,1157366748	38 / 0
Matrix metalloproteinase 2	MMP2	P08253	CHEMBL333	Protease	0,1157366748	384 / 0
Bile acid receptor FXR	NR1H4	Q96RI1	CHEMBL2047	Nuclear receptor	0,1157366748	12 / 0
Beta-secretase 1	BACE1	P56817	CHEMBL4822	Protease	0,1157366748	399 / 0
Serine/threonine-protein kinase Aurora-B	AURKB	Q96GD4	CHEMBL2185	Kinase	0,1157366748	107 / 0
Serine/threonine-protein kinase Aurora-A	AURKA	O14965	CHEMBL4722	Kinase	0,1157366748	186 / 0
Cyclin-dependent kinase 2/cyclin A	CDK2<br>CCNA1<br>CCNA2	P24941<br>P78396<br>P20248	CHEMBL2094128	Other cytosolic protein	0,1157366748	37 / 0
Tyrosine-protein kinase JAK1	JAK1	P23458	CHEMBL2835	Kinase	0,1157366748	35 / 0
Tyrosine-protein kinase JAK2	JAK2	O60674	CHEMBL2971	Kinase	0,1157366748	24 / 0
MAP kinase signal-integrating kinase 2	MKNK2	Q9HBH9	CHEMBL4204	Kinase	0,1157366748	13 / 0
Phosphodiesterase 5A	PDE5A	O76074	CHEMBL1827	Phosphodiesterase	0,1157366748	48 / 0
Ephrin receptor	EPHB4	P54760	CHEMBL5147	Kinase	0,1157366748	15 / 0
TNF-alpha	TNF	P01375	CHEMBL1825	Secreted protein	0,1157366748	12 / 0
ADAM17	ADAM17	P78536	CHEMBL3706	Protease	0,1157366748	210 / 0
Adenosine A1 receptor (by homology)	ADORA1	P30542	CHEMBL226	Family A G protein-coupled receptor	0,1157366748	45 / 0
Glycogen synthase kinase-3 beta	GSK3B	P49841	CHEMBL262	Kinase	0,1157366748	58 / 0
Glycogen synthase kinase-3 alpha	GSK3A	P49840	CHEMBL2850	Kinase	0,1157366748	5 / 0
Arachidonate 5-lipoxygenase	ALOX5	P09917	CHEMBL215	Oxidoreductase	0,1157366748	86 / 0
Matrix metalloproteinase 7	MMP7	P09237	CHEMBL4073	Protease	0,1157366748	94 / 0
Matrix metalloproteinase 14	MMP14	P50281	CHEMBL3869	Protease	0,1157366748	57 / 0
ADAM10	ADAM10	O14672	CHEMBL5028	Protease	0,1157366748	11 / 0
Cyclin-dependent kinase 2	CDK2	P24941	CHEMBL301	Kinase	0,1157366748	42 / 0
Cyclin-dependent kinase 4	CDK4	P11802	CHEMBL331	Kinase	0,1157366748	23 / 0
MAP kinase ERK2 (by homology)	MAPK1	P28482	CHEMBL4040	Kinase	0,1157366748	127 / 0
Histone deacetylase 6	HDAC6	Q9UBN7	CHEMBL1865	Eraser	0,1157366748	216 / 0
Histone deacetylase 1	HDAC1	Q13547	CHEMBL325	Eraser	0,1157366748	243 / 0
Histone deacetylase 3	HDAC3	O15379	CHEMBL1829	Eraser	0,1157366748	120 / 0
Histone deacetylase 2	HDAC2	Q92769	CHEMBL1937	Eraser	0,1157366748	137 / 0
Histone deacetylase 8	HDAC8	Q9BY41	CHEMBL3192	Eraser	0,1157366748	123 / 0
Matrix metalloproteinase 16	MMP16	P51512	CHEMBL2200	Protease	0,1157366748	14 / 0
Matrix metalloproteinase 15	MMP15	P51511	CHEMBL2963	Protease	0,1157366748	5 / 0
Matrix metalloproteinase 12	MMP12	P39900	CHEMBL4393	Protease	0,1157366748	23 / 0

Matrix metalloproteinase 26	MMP26	Q9NRE1	CHEMBL4707	Protease	0,1157366748	4 / 0
Cyclin-dependent kinase 1/cyclin B1	CDK1<br>CCNB1	P06493<br>P14635	CHEMBL1907602	Other cytosolic protein	0,1157366748	15 / 0
Cyclin-dependent kinase 2/cyclin E1	CCNE1<br>CDK2	P24864<br>P24941	CHEMBL1907605	Kinase	0,1157366748	15 / 0
Estrogen receptor alpha	ESR1	P03372	CHEMBL206	Nuclear receptor	0,1157366748	93 / 0
Serine/threonine-protein kinase PIM1	PIM1	P11309	CHEMBL2147	Kinase	0,1157366748	22 / 0
Mu opioid receptor	OPRM1	P35372	CHEMBL233	Family A G protein-coupled receptor	0,1157366748	46 / 0
Delta opioid receptor	OPRD1	P41143	CHEMBL236	Family A G protein-coupled receptor	0,1157366748	37 / 0
Kappa Opioid receptor	OPRK1	P41145	CHEMBL237	Family A G protein-coupled receptor	0,1157366748	36 / 0
Estrogen receptor beta	ESR2	Q92731	CHEMBL242	Nuclear receptor	0,1157366748	84 / 0
Nerve growth factor receptor Trk-A	NTRK1	P04629	CHEMBL2815	Kinase	0,1157366748	21 / 0
Serotonin 7 (5-HT7) receptor	HTR7	P34969	CHEMBL3155	Family A G protein-coupled receptor	0,1157366748	6 / 0
Serine/threonine-protein kinase PIM2	PIM2	Q9P1W9	CHEMBL4523	Kinase	0,1157366748	10 / 0
Serine/threonine-protein kinase PIM3	PIM3	Q86V86	CHEMBL5407	Kinase	0,1157366748	5 / 0
Cyclooxygenase-2	PTGS2	P35354	CHEMBL230	Oxidoreductase	0,1157366748	51 / 0
Transient receptor potential cation channel subfamily M member 8 (by homology)	TRPM8	Q7Z2W7	CHEMBL1075319	Voltage-gated ion channel	0,1157366748	13 / 0
Matrix metalloproteinase 25	MMP25	Q9NPA2	CHEMBL1795103	Enzyme	0,1157366748	1 / 0
Carbonic anhydrase II	CA2	P00918	CHEMBL205	Lyase	0,1157366748	89 / 0
Cyclooxygenase-1	PTGS1	P23219	CHEMBL221	Oxidoreductase	0,1157366748	15 / 0
Histone deacetylase 4	HDAC4	P56524	CHEMBL3524	Eraser	0,1157366748	25 / 0
Carbonic anhydrase IX	CA9	Q16790	CHEMBL3594	Lyase	0,1157366748	65 / 0
Hepatocyte growth factor receptor	MET	P08581	CHEMBL3717	Kinase	0,1157366748	109 / 0
Sodium/glucose cotransporter 2	SLC5A2	P31639	CHEMBL3884	Electrochemical transporter	0,1157366748	4 / 0
Aminopeptidase N	ANPEP	P15144	CHEMBL1907	Protease	0,1157366748	12 / 0
Cytochrome P450 19A1	CYP19A1	P11511	CHEMBL1978	Cytochrome P450	0,1157366748	26 / 0
Sodium/glucose cotransporter 1	SLC5A1	P13866	CHEMBL4979	Electrochemical transporter	0,1157366748	27 / 0
Aldose reductase (by homology)	AKR1B1	P15121	CHEMBL1900	Enzyme	0,1157366748	65 / 0
Serine/threonine-protein kinase Sgk1	SGK1	O00141	CHEMBL2343	Kinase	0,1157366748	4 / 0
Pyruvate dehydrogenase kinase isoform 1	PDK1	Q15118	CHEMBL4766	Kinase	0,1157366748	47 / 0
Protein-tyrosine phosphatase 1B	PTPN1	P18031	CHEMBL335	Phosphatase	0,1157366748	25 / 0
Phospholipase A2 group IIA	PLA2G2A	P14555	CHEMBL3474	Enzyme	0,1157366748	1 / 0
Insulin-like growth factor I receptor	IGF1R	P08069	CHEMBL1957	Kinase	0,1157366748	10 / 0
Vascular endothelial growth factor receptor 2	KDR	P35968	CHEMBL279	Kinase	0,1157366748	150 / 0
Epidermal growth factor receptor erbB1	EGFR	P00533	CHEMBL203	Kinase	0,1157366748	96 / 0
Neuronal acetylcholine receptor protein alpha-7 subunit	CHRNA7	P36544	CHEMBL2492	Ligand-gated ion channel	0,1157366748	14 / 0
Sphingosine kinase 2	SPHK2	Q9NRA0	CHEMBL3023	Enzyme	0,1157366748	2 / 0
Sphingosine kinase 1	SPHK1	Q9NYA1	CHEMBL4394	Enzyme	0,1157366748	2 / 0
P2X purinoceptor 7	P2RX7	Q99572	CHEMBL4805	Ligand-gated ion channel	0,1157366748	8 / 0
Serine/threonine-protein kinase B-raf	BRAF	P15056	CHEMBL5145	Kinase	0,1157366748	53 / 0
Histone-lysine N-methyltransferase, H3 lysine-9 specific 3	EHMT2	Q96KQ7	CHEMBL6032	Writer	0,1157366748	3 / 0

Trace amine-associated receptor 1 (by homology)	TAAR1	Q96RJ0	CHEMBL5857	Family A G protein-coupled receptor	0,1157366748	12 / 0
ATP-binding cassette sub-family G member 2	ABCG2	Q9UNQ0	CHEMBL5393	Primary active transporter	0,1157366748	25 / 0
Equilibrative nucleoside transporter 1	SLC29A1	Q99808	CHEMBL1997	Electrochemical transporter	0,1157366748	5 / 0
Thrombin	F2	P00734	CHEMBL204	Protease	0,1157366748	97 / 0
Tissue-type plasminogen activator	PLAT	P00750	CHEMBL1873	Protease	0	11 / 0
Monoamine oxidase A	MAOA	P21397	CHEMBL1951	Oxidoreductase	0	36 / 0
Monoamine oxidase B	MAOB	P27338	CHEMBL2039	Oxidoreductase	0	39 / 0
Thrombin and coagulation factor X	F10	P00742	CHEMBL244	Protease	0	76 / 0
Dual specificity protein phosphatase 3	DUSP3	P51452	CHEMBL2635	Phosphatase	0	6 / 0
Induced myeloid leukemia cell differentiation protein Mcl-1	MCL1	Q07820	CHEMBL4361	Other cytosolic protein	0	11 / 0
CDC7/DBF4 (Cell division cycle 7-related protein kinase/Activator of S phase kinase)	CDC7	O00311	CHEMBL5443	Kinase	0	7 / 0
Aldo-keto reductase family 1 member B10	AKR1B10	O60218	CHEMBL5983	Enzyme	0	19 / 0
ADAMTS5	ADAMTS5	Q9UNA0	CHEMBL2285	Protease	0	20 / 0
Cathepsin K	CTSK	P43235	CHEMBL268	Protease	0	6 / 0
Phosphodiesterase 10A	PDE10A	Q9Y233	CHEMBL4409	Phosphodiesterase	0	52 / 0
Adenosine A2a receptor	ADORA2A	P29274	CHEMBL251	Family A G protein-coupled receptor	0	29 / 0
Immunoglobulin epsilon Fc receptor	FCER2	P06734	CHEMBL2940	Membrane receptor	0	18 / 0
Cathepsin L	CTSL	P07711	CHEMBL3837	Protease	0	11 / 0
Plasminogen	PLG	P00747	CHEMBL1801	Protease	0	23 / 0
Taste receptor type 2 member 31	TAS2R31	P59538	CHEMBL2034804	Taste family G protein-coupled receptor	0	3 / 0

Table S2. Individual AutoDockVina scores for the ligands docked to the predicted target protein CDK2. PDB ID—identificator of PDB structure of the target protein with co-crystallized ligand (CCL). CCL ID—combination of PDB ID, residue name, and residue number that identifies the CCL structure in the source PDB file. R, S—enantiomers of Les-6547 and Les-6557, respectively. Values in the "CCL" column represent individual Vina scores for different CCLs bound to a different conformation of the predicted target, evaluated by AutoDock Vina in score-only mode without performing docking. The remaining four columns contain individual Vina docking scores of Les-6547 and Les-6557 enantiomers docked to the same conformations of the target that bind CCLs. For each of n target conformations, the most favorable docking score was selected. The CCLs were removed from the protein structures as the latter were prepared for docking.

Target Protein	PDB ID	CCL ID	CCL Name	Vina scores, kkal/mol				
				CCL	Les-6547		Les-6557	
					R	S	R	S
CDK2	1AQ1	1AQ1-STU-299	STAUROSPORINE	-11.92	-9.22	-8.91	-9.98	-9.62
CDK2	1B38	1B38-ATP-381	ADENOSINE-5'-TRIPHOSPHATE	-7.43	-9.42	-9.05	-9.44	-9.13
CDK2	1B39	1B39-ATP-381	ADENOSINE-5'-TRIPHOSPHATE	-6.36	-9.81	-8.86	-9.80	-9.07
CDK2	1DI8	1DI8-DTQ-500	4-[3-HYDROXYANILINO]-6,7-DIMETHOXYQUINAZOLINE	-6.07	-8.90	-8.59	-8.92	-9.64
CDK2	1E1V	1E1V-CMG-401	6-O-CYCLOHEXYLMETHYL GUANINE	-5.52	-7.32	-3.95	-7.45	-5.72
CDK2	1E1X	1E1X-NW1-401	6-CYCLOHEXYLMETHYLOXY-5-NITROSO-PYRIMIDINE-2,4-DIAMINE	-4.90	-8.21	-7.53	-8.65	-8.81
CDK2	1E9H	1E9H-INR-1298	2',3-DIOXO-1,1',2',3-TETRAHYDRO-2,3'-BIINDOLE-5'-SULFONIC ACID	-9.90	-8.95	-10.30	-10.54	-10.71
CDK2	1FIN	1FIN-ATP-299	ADENOSINE-5'-TRIPHOSPHATE	-3.04	-9.64	-9.41	-9.51	-9.62
CDK2	1FQ1	1FQ1-ATP-381	ADENOSINE-5'-TRIPHOSPHATE	-5.57	-9.19	-9.51	-9.43	-9.58
CDK2	1FVT	1FVT-106-299	4-[(2Z)-2-(5-BROMO-2-OXO-1,2-DIHYDRO-3H-INDOL-3-YLIDENE)HYDRAZINYL]BENZENE-1-SULFONAMIDE	-7.38	-8.37	-9.18	-9.85	-10.50
CDK2	1FVV	1FVV-107-501	4-[(7-OXO-7H-THIAZOLO[5,4-E]INDOL-8-YLMETHYL)-AMINO]-N-PYRIDIN-2-YL-BENZENESULFONAMIDE	-8.77	-8.63	-9.10	-9.55	-9.04
CDK2	1G5S	1G5S-I17-400	2-[TRANS-(4-AMINOCYCLOHEXYL)AMINO]-6-(BENZYL-AMINO)-9-CYCLOPENTYLPURINE	-9.24	-9.62	-9.40	-10.19	-9.20
CDK2	1GIH	1GIH-1PU-501	1-(5-OXO-2,3,5,9B-TETRAHYDRO-1H-PYRROLO[2,1-A]ISOINDOL-9-YL)-3-PYRIDIN-2-YL-UREA	-9.95	-7.78	-8.28	-9.01	-9.68
CDK2	1GII	1GII-1PU-501	1-(5-OXO-2,3,5,9B-TETRAHYDRO-1H-PYRROLO[2,1-A]ISOINDOL-9-YL)-3-PYRIDIN-2-YL-UREA	-8.77	-8.67	-8.99	-9.07	-8.74
CDK2	1GIJ	1GIJ-2PU-501	1-(5-OXO-2,3,5,9B-TETRAHYDRO-1H-PYRROLO[2,1-A]ISOINDOL-9-YL)-3-(5-PYRROLIDIN-2-YL-1H-PYRAZOL-3-YL)-UREA	-9.94	-7.77	-8.53	-8.93	-8.09
CDK2	1GY3	1GY3-ATP-1297	ADENOSINE-5'-TRIPHOSPHATE	-2.64	-9.48	-9.26	-9.62	-9.40
CDK2	1H08	1H08-BWP-300	(2S)-1-{4-[(4-ANILINO-5-BROMOPYRIMIDIN-2-YL)AMINO]PHENOXY}-3-(DIMETHYLAMINO)PROPAN-2-OL	-6.49	-8.50	-7.98	-9.28	-8.48
CDK2	1H1P	1H1P-CMG-1298	6-O-CYCLOHEXYLMETHYL GUANINE	-6.97	-6.05	-7.71	-7.91	-8.35
CDK2	1H1Q	1H1Q-2A6-1298	2-ANILINO-6-CYCLOHEXYLMETHOXYPURINE	-7.50	-9.04	-9.46	-9.51	-9.28
CDK2	1H1R	1H1R-6CP-1298	6-CYCLOHEXYLMETHOXY-2-(3'-CHLOROANILINO) PURINE	-7.61	-9.32	-8.10	-8.83	-9.55
CDK2	1H1S	1H1S-4SP-1298	O6-CYCLOHEXYLMETHOXY-2-(4'-SULPHAMOYLANILINO) PURINE	-6.84	-8.32	-8.43	-8.47	-9.83
CDK2	1JST	1JST-ATP-300	ADENOSINE-5'-TRIPHOSPHATE	-5.92	-10.91	-10.90	-10.97	-10.96
CDK2	1JSV	1JSV-U55-400	4-[(6-AMINO-4-PYRIMIDINYL)AMINO]BENZENESULFONAMIDE	-4.15	-8.05	-7.73	-7.98	-8.25
CDK2	1KE5	1KE5-LS1-299	N-METHYL-4-{[(2-OXO-1,2-DIHYDRO-3H-INDOL-3-YLIDENE)METHYL]AMINO}BENZENESULFONAMIDE	-8.80	-7.83	-7.43	-7.96	-7.85
CDK2	1KE6	1KE6-LS2-299	N-METHYL-{4-[2-(7-OXO-6,7-DIHYDRO-8H-[1,3]THIAZOLO[5,4-E]INDOL-8-YLIDENE)HYDRAZINO]PHENYL}METHANESULFONAMIDE	-8.34	-7.24	-8.34	-9.08	-9.60
CDK2	1KE7	1KE7-LS3-299	3-{[(2,2-DIOXIDO-1,3-DIHYDRO-2-BENZOTHIEN-5-YL)AMINO]METHYLENE}-5-(1,3-OXAZOL-5-YL)-1,3-DIHYDRO-2H-INDOL-2-ONE	-8.08	-7.47	-7.29	-8.24	-10.06
CDK2	1KE8	1KE8-LS4-299	4-{[(2-OXO-1,2-DIHYDRO-3H-INDOL-3-YLIDENE)METHYL]AMINO}-N-(1,3-THIAZOL-2-YL)BENZENESULFONAMIDE	-8.44	-7.87	-7.63	-8.23	-7.70
CDK2	1KE9	1KE9-LS5-299	3-{[4-([AMINO(IMINO)METHYL]AMINOSULFONYL)ANILINO]METHYLENE}-2-OXO-2,3-DIHYDRO-1H-INDOLE	-7.95	-7.83	-8.40	-8.57	-9.19
CDK2	1OIQ	1OIQ-HDU-1299	N-[4-(2-METHYLIMIDAZO[1,2-A]PYRIDIN-3-YL)-2-PYRIMIDINYL]ACETAMIDE	-6.17	-8.31	-7.88	-8.36	-8.22

CDK2	1OIR	1OIR-HDY-1298	1-(DIMETHYLAMINO)-3-(4-{{4-(2-METHYLIMIDAZO[1,2-A]PYRIDIN-3-YL)PYRIMIDIN-2-YL]AMINO}PHENOXY)PROPAN-2-OL	-7.58	-9.58	-8.63	-10.20	-9.12
CDK2	1OIT	1OIT-HDT-1299	4-[(4-IMIDAZO[1,2-A]PYRIDIN-3-YLPYRIMIDIN-2-YL)AMINO]BENZENESULFONAMIDE	-7.91	-9.35	-9.23	-8.98	-9.32
CDK2	1P2A	1P2A-5BN-301	5-[(2-AMINOETHYL)AMINO]-6-FLUORO-3-(1H-PYRROL-2-YL)BENZO[CD]INDOL-2(1H)-ONE	-8.67	-7.95	-8.39	-9.47	-8.05
CDK2	1P5E	1P5E-TBS-301	4,5,6,7-TETRABROMOBENZOTRIAZOLE	-4.58	-8.62	-9.39	-9.74	-9.48
CDK2	1PF8	1PF8-SU9-500	(3Z)-3-(1H-IMIDAZOL-5-YLMETHYLENE)-5-METHOXY-1H-INDOL-2(3H)-ONE	-7.96	-6.25	-4.98	-8.63	-7.14
CDK2	1PKD	1PKD-UCN-410	7-HYDROXYSTAUROSPORINE	-11.54	-9.53	-9.94	-9.78	-10.05
CDK2	1PYE	1PYE-PM1-700	[2-AMINO-6-(2,6-DIFLUORO-BENZOYL)-IMIDAZO[1,2-A]PYRIDIN-3-YL]-PHENYL-METHANONE	-7.53	-9.72	-10.09	-9.33	-10.03
CDK2	1QMZ	1QMZ-ATP-381	ADENOSINE-5'-TRIPHOSPHATE	-5.86	-10.15	-10.02	-10.10	-9.62
CDK2	1VYW	1VYW-292-2300	N-(3-CYCLOPROPYL-1H-PYRAZOL-5-YL)-2-(2-NAPHTHYL)ACETAMIDE	-8.03	-10.40	-9.44	-9.39	-9.69
CDK2	1VYZ	1VYZ-N5B-300	N-(5-CYCLOPROPYL-1H-PYRAZOL-3-YL)BENZAMIDE	-7.18	-8.44	-8.33	-9.60	-7.82
CDK2	1W0X	1W0X-OLO-1299	OLOMOUCINE	-6.30	-9.15	-9.31	-8.90	-9.28
CDK2	1Y8Y	1Y8Y-CT7-401	(5-CHLOROPYRAZOLO[1,5-A]PYRIMIDIN-7-YL)-(4-METHANESULFONYLPHENYL)AMINE	-6.97	-8.33	-9.65	-8.23	-9.32
CDK2	1Y91	1Y91-CT9-401	4-[5-(TRANS-4-AMINOCYCLOHEXYLAMINO)-3-ISOPROPYLPYRAZOLO[1,5-A]PYRIMIDIN-7-YLAMINO]-N,N-DIMETHYLBENZENESULFONAMIDE	-8.99	-9.28	-9.09	-10.72	-9.21
CDK2	1YKR	1YKR-628-299	4-{[6-(2,6-DICHLOROBENZOYL)IMIDAZO[1,2-A]PYRIDIN-2-YL]AMINO}BENZENESULFONAMIDE	-7.37	-9.60	-9.23	-10.29	-9.92
CDK2	2A0C	2A0C-CK9-500	2-{[(2-{[(1R)-1-(HYDROXYMETHYL)PROPYL]AMINO}-9-ISOPROPYL-9H-PURIN-6-YL)AMINO]METHYL}PHENOL	-6.91	-9.91	-9.05	-10.36	-9.18
CDK2	2A4L	2A4L-RRC-300	R-ROSCOVITINE	-6.95	-9.80	-8.53	-10.18	-8.92
CDK2	2B52	2B52-D42-299	1-(3-(2,4-DIMETHYLTHIAZOL-5-YL)-4-OXO-2,4-DIHYDROINDENO[1,2-C]PYRAZOL-5-YL)-3-(4-METHYLPIPERAZIN-1-YL)UREA	-9.14	-8.91	-9.85	-9.80	-9.46
CDK2	2B53	2B53-D23-299	6-(3-AMINOPHENYL)-N-(TERT-BUTYL)-2-(TRIFLUOROMETHYL)QUINAZOLIN-4-AMINE	-7.78	-9.75	-9.96	-10.05	-9.75
CDK2	2B54	2B54-D05-300	6-(3,4-DIHYDROXYBENZYL)-3-ETHYL-1-(2,4,6-TRICHLOROPHENYL)-1H-PYRAZOLO[3,4-D]PYRIMIDIN-4(5H)-ONE	-9.14	-7.97	-8.33	-8.03	-8.20
CDK2	2B55	2B55-D31-299	2-(4-(AMINOMETHYL)PIPERIDIN-1-YL)-N-(3_CYCLOHEXYL-4-OXO-2,4-DIHYDROINDENO[1,2-C]PYRAZOL-5-YL)ACETAMIDE	-10.19	-9.39	-10.49	-10.42	-10.65
CDK2	2BHE	2BHE-BRY-1299	(2Z)-5'-BROMO-2,3'-BIINDOLE-2',3(1H,1'H)-DIONE AMMONIATE	-8.45	-7.59	-7.28	-8.88	-7.78
CDK2	2BHH	2BHH-RYU-1299	(2E,3S)-3-HYDROXY-5'-[(4-HYDROXYPIPERIDIN-1-YL)SULFONYL]-3-METHYL-1,3-DIHYDRO-2,3'-BIINDOL-2'(1'H)-ONE	-9.45	-8.43	-8.53	-9.20	-9.40
CDK2	2BKZ	2BKZ-SBC-1298	1-[4-(AMINOSULFONYL)PHENYL]-1,6-DIHYDROPYRAZOLO[3,4-E]INDAZOLE-3-CARBOXAMIDE	-7.88	-9.64	-10.47	-9.99	-9.77
CDK2	2BPM	2BPM-529-1299	(2S)-N-[(3Z)-5-CYCLOPROPYL-3H-PYRAZOL-3-YLIDENE]-2-[4-(2-OXOIMIDAZOLIDIN-1-YL)PHENYL]PROPANAMIDE	-7.62	-9.80	-9.44	-9.28	-9.66
CDK2	2BTR	2BTR-U73-300	N-(5-ISOPROPYL-THIAZOL-2-YL)-2-PYRIDIN-3-YL-ACETAMIDE	-6.28	-9.16	-7.74	-9.89	-8.51
CDK2	2BTS	2BTS-U32-1299	4-[(5-ISOPROPYL-1,3-THIAZOL-2-YL)AMINO]BENZENESULFONAMIDE	-7.03	-9.82	-9.46	-9.68	-10.03
CDK2	2C4G	2C4G-514-306	(3Z)-5-ACETYL-3-(BENZOYLIMINO)-3,6-DIHYDROPYRROLO[3,4-C]PYRAZOL-5-IUM	-8.03	-9.45	-9.79	-9.68	-9.38
CDK2	2C5N	2C5N-CK8-1297	N-[4-(2,4-DIMETHYL-THIAZOL-5-YL)-PYRIMIDIN-2-YL]-N',N'-DIMETHYL-BENZENE-1,4-DIAMINE	-5.61	-9.34	-9.16	-9.67	-10.84
CDK2	2C5X	2C5X-MTW-1297	HYDROXY(OXO)(3-{[(2Z)-4-[3-(1H-1,2,4-TRIAZOL-1-YLMETHYL)PHENYL]PYRIMIDIN-2(5H)-YLIDENE]AMINO}PHENYL)AMMONIUM	-5.35	-10.50	-10.11	-10.16	-9.74

CDK2	2C68	2C68-CT6-1297	(5Z)-5-(3-BROMOCYCLOHEXA-2,5-DIEN-1-YLIDENE)-N-(PYRIDIN-4-YLMETHYL)-1,5-DIHYDROPYRAZOLO[1,5-A]PYRIMIDIN-7-AMINE	-7.71	-5.27	-4.60	-6.54	-4.38
CDK2	2C69	2C69-CT8-1299	(5Z)-5-(3-BROMOCYCLOHEXA-2,5-DIEN-1-YLIDENE)-N-(PYRIDIN-4-YLMETHYL)-1,5-DIHYDRO[1,2,4]TRIAZOLO[1,5-A]PYRIMIDIN-7-AMINE	-7.06	-7.59	-5.42	-7.16	-5.78
CDK2	2C6I	2C6I-DT1-1299	4-{[5-(CYCLOHEXYLMETHOXY)[1,2,4]TRIAZOLO[1,5-A]PYRIMIDIN-7-YL]AMINO}BENZENESULFONAMIDE	-7.96	-8.56	-8.93	-8.97	-8.68
CDK2	2C6K	2C6K-DT2-1299	4-{[5-(CYCLOHEXYLAMINO)[1,2,4]TRIAZOLO[1,5-A]PYRIMIDIN-7-YL]AMINO}BENZENESULFONAMIDE	-7.21	-9.48	-9.06	-8.81	-9.01
CDK2	2C6L	2C6L-DT4-1299	4-({5-[(4-AMINOCYCLOHEXYL)AMINO][1,2,4]TRIAZOLO[1,5-A]PYRIMIDIN-7-YL}AMINO)BENZENESULFONAMIDE	-7.87	-8.84	-9.44	-9.42	-9.29
CDK2	2C6M	2C6M-DT5-1297	4-{[5-(CYCLOHEXYLOXY)[1,2,4]TRIAZOLO[1,5-A]PYRIMIDIN-7-YL]AMINO}BENZENESULFONAMIDE	-7.80	-8.23	-8.20	-8.24	-8.36
CDK2	2C6O	2C6O-4SP-1297	O6-CYCLOHEXYLMETHOXY-2-(4'-SULPHAMOYLANILINO) PURINE	-7.66	-8.72	-9.28	-8.77	-9.01
CDK2	2C6T	2C6T-DT5-1297	4-{[5-(CYCLOHEXYLOXY)[1,2,4]TRIAZOLO[1,5-A]PYRIMIDIN-7-YL]AMINO}BENZENESULFONAMIDE	-8.30	-8.44	-9.56	-8.90	-8.60
CDK2	2CCI	2CCI-ATP-1297	ADENOSINE-5'-TRIPHOSPHATE	-5.96	-9.01	-9.98	-9.34	-10.01
CDK2	2CJM	2CJM-ATP-1294	ADENOSINE-5'-TRIPHOSPHATE	-5.32	-9.32	-9.74	-9.77	-9.88
CDK2	2DS1	2DS1-1CD-501	(13R,15S)-13-METHYL-16-OXA-8,9,12,22,24-PENTAAZAHEXACYCLO[15.6.2.16,9.1,12,15.0,2,7.0,21,25]HEPTACOSA-1(24),2,4,6,17(25),18,20-HEPTAENE-23,26-DIONE	-12.86	-9.77	-9.89	-10.18	-10.15
CDK2	2DUV	2DUV-371-501	2-(3,4-DIHYDROXYPHENYL)-8-(1,1-DIOXIDOISOTHIAZOLIDIN-2-YL)-3-HYDROXY-6-METHYL-4H-CHROMEN-4-ONE	-8.27	-9.09	-8.98	-9.22	-9.88
CDK2	2EXM	2EXM-ZIP-400	N-(3-METHYLBUT-2-EN-1-YL)-9H-PURIN-6-AMINE	-4.77	-8.72	-8.82	-8.72	-8.54
CDK2	2FVD	2FVD-LIA-299	(4-AMINO-2-{[1-(METHYLSULFONYL)PIPERIDIN-4-YL]AMINO}PYRIMIDIN-5-YL)(2,3-DIFLUORO-6-METHOXYPHENYL)METHANONE	-8.70	-9.77	-9.20	-9.51	-9.84
CDK2	2G9X	2G9X-NU5-299	3-({2-[(4-{[6-(CYCLOHEXYLMETHOXY)-9H-PURIN-2-YL]AMINO}PHENYL)SULFONYL]ETHYL}AMINO)PROPAN-1-OL	-6.31	-7.75	-7.76	-8.35	-7.94
CDK2	2IW6	2IW6-QQ2-1296	[(2-CHLORO-5-METHYLPHENYL){6-[(4-{[(2R)-3-(DIMETHYLAMINO)-2-HYDROXYPROPYL]OXY}PHENYL)AMINO]PYRIMIDIN-4-YL}AMINO]ACETONITRILE	-7.97	-9.03	-8.55	-9.09	-8.61
CDK2	2R64	2R64-740-500	N-[5-(1,1-DIOXIDOISOTHIAZOLIDIN-2-YL)-1H-INDAZOL-3-YL]-2-(4-PIPERIDIN-1-YLPHENYL)ACETAMIDE	-4.95	-10.00	-8.67	-10.03	-8.88
CDK2	2UUE	2UUE-GVC-1433	1-(3,5-DICHLOROPHENYL)-5-METHYL-1H-1,2,4-TRIAZOLE-3-CARBOXYLIC ACID	4.00	-7.26	-7.39	-7.35	-6.91
CDK2	2UZD	2UZD-C85-1297	4-{5-[(Z)-(2-IMINO-4-OXO-1,3-THIAZOLIDIN-5-YLIDENE)METHYL]FURAN-2-YL}BENZENESULFONAMIDE	-6.45	-9.15	-9.01	-9.90	-9.42
CDK2	2UZE	2UZE-C95-1297	4-{5-[(Z)-(2-IMINO-4-OXO-1,3-THIAZOLIDIN-5-YLIDENE)METHYL]FURAN-2-YL}BENZOIC ACID	-7.92	-8.55	-8.91	-8.85	-10.04
CDK2	2UZL	2UZL-C94-1297	4-{5-[(Z)-(2-IMINO-4-OXO-1,3-THIAZOLIDIN-5-YLIDENE)METHYL]FURAN-2-YL}-2-(TRIFLUOROMETHYL)BENZENESULFONAMIDE	-7.87	-8.07	-8.73	-8.11	-9.77
CDK2	2UZN	2UZN-C96-1299	4-{5-[(1Z)-1-(2-IMINO-4-OXO-1,3-THIAZOLIDIN-5-YLIDENE)ETHYL]-2-FURYL}BENZENESULFONAMIDE	-6.27	-8.21	-8.66	-9.66	-9.41
CDK2	2UZO	2UZO-C62-1297	4-{5-[(Z)-(2,4-DIOXO-1,3-THIAZOLIDIN-5-YLIDENE)METHYL]FURAN-2-YL}BENZENESULFONAMIDE	-6.73	-8.37	-8.65	-8.52	-8.70
CDK2	2V0D	2V0D-C53-1299	2-IMINO-5-(1-PYRIDIN-2-YL-METH-(E)-YLIDENE)-1,3-THIAZOLIDIN-4-ONE	-3.81	-7.74	-6.90	-7.34	-8.40
CDK2	2V22	2V22-C35-1433	N~2~-{[1-(4-CHLOROPHENYL)-5-METHYL-1H-1,2,4-TRIAZOL-3-YL]CARBONYL}-N~5~-(DIAMINOMETHYLIDENE)-L-ORNITHYL-L-LEUCYL-L-ISOLEUCYL-4-FLUORO-L-PHENYLALANINAMIDE	-6.01	-7.27	-7.16	-7.10	-7.27
CDK2	2VTH	2VTH-LZ2-1300	5-HYDROXYNAPHTHALENE-1-SULFONAMIDE	-6.30	-8.99	-8.99	-9.74	-8.76
CDK2	2VTI	2VTI-LZ3-1299	N-(4-SULFAMOYLPHENYL)-1H-INDAZOLE-3-CARBOXAMIDE	-8.15	-9.02	-8.81	-9.45	-9.94

CDK2	2VTJ	2VTJ-LZ4-1300	4-[(6-CHLOROPYRAZIN-2-YL)AMINO]BENZENESULFONAMIDE	-5.35	-8.81	-8.30	-9.74	-8.83
CDK2	2VTN	2VTN-LZ7-1299	4-(ACETYLAMINO)-N-(4-FLUOROPHENYL)-1H-PYRAZOLE-3-CARBOXAMIDE	-5.29	-6.92	-7.99	-8.52	-8.88
CDK2	2VTQ	2VTQ-LZA-1299	{[(2,6-DIFLUOROPHENYL)CARBONYL]AMINO}-N-PIPERIDIN-4-YL-1H-PYRAZOLE-3-CARBOXAMIDE	-8.50	-9.62	-10.28	-10.23	-10.70
CDK2	2VTS	2VTS-LZC-1299	5-[(4-AMINOCYCLOHEXYL)AMINO]-7-(PROPAN-2-YLAMINO)PYRAZOLO[1,5-A]PYRIMIDINE-3-CARBONITRILE	-7.12	-7.94	-9.01	-8.05	-8.50
CDK2	2VTT	2VTT-LZD-1299	4-{[(2,6-DIFLUOROPHENYL)CARBONYL]AMINO}-N-[(3S)-PIPERIDIN-3-YL]-1H-PYRAZOLE-3-CARBOXAMIDE	-8.01	-10.09	-9.69	-10.45	-9.99
CDK2	2W05	2W05-FRT-1299	N-(2-METHOXYETHYL)-4-({4-[2-METHYL-1-(1-METHYLETHYL)-1H-IMIDAZOL-5-YL]PYRIMIDIN-2-YL}AMINO)BENZENESULFONAMIDE	-7.52	-9.62	-9.06	-9.93	-9.19
CDK2	2W06	2W06-FRV-1300	4-{[4-(1-CYCLOPROPYL-2-METHYL-1H-IMIDAZOL-5-YL)PYRIMIDIN-2-YL]AMINO}-N-METHYLBENZENESULFONAMIDE	-8.74	-9.70	-8.85	-9.82	-9.25
CDK2	2W1H	2W1H-L0F-1299	N-[3-(1H-BENZIMIDAZOL-2-YL)-1H-PYRAZOL-4-YL]BENZAMIDE	-8.29	-7.87	-7.97	-9.27	-8.56
CDK2	2WIH	2WIH-P48-1299	N,1,4,4-TETRAMETHYL-8-{[4-(4-METHYLPIPERAZIN-1-YL)PHENYL]AMINO}-4,5-DIHYDRO-1H-PYRAZOLO[4,3-H]QUINAZOLINE-3-CARBOXAMIDE	-10.81	-9.46	-9.05	-10.45	-9.91
CDK2	2WIP	2WIP-P49-1297	1-METHYL-8-(PHENYLAMINO)-4,5-DIHYDRO-1H-PYRAZOLO[4,3-H]QUINAZOLINE-3-CARBOXYLIC ACID	-9.59	-9.97	-9.06	-9.99	-8.95
CDK2	2WPA	2WPA-889-1301	N-{6,6-DIMETHYL-5-[(1-METHYLPIPERIDIN-4-YL)CARBONYL]-1,4,5,6-TETRAHYDROPYRROLO[3,4-C]PYRAZOL-3-YL}-3-METHYLBUTANAMIDE	-8.33	-9.52	-10.26	-10.20	-10.01
CDK2	2WXV	2WXV-WXV-1299	N,1-DIMETHYL-8-{[1-(METHYLSULFONYL)PIPERIDIN-4-YL]AMINO}-1H-PYRAZOLO[4,3-H]QUINAZOLINE-3-CARBOXAMIDE	-9.52	-9.88	-9.78	-9.77	-9.28
CDK2	3EID	3EID-PO5-299	(2S)-1-(DIMETHYLAMINO)-3-(4-{[4-(6-MORPHOLIN-4-YLPYRAZOLO[1,5-B]PYRIDAZIN-3-YL)PYRIMIDIN-2-YL]AMINO}PHENOXY)PROPAN-2-OL	-8.14	-10.04	-10.04	-9.95	-10.28
CDK2	3EJ1	3EJ1-5BP-299	N-CYCLOPROPYL-4-PYRAZOLO[1,5-B]PYRIDAZIN-3-YLPYRIMIDIN-2-AMINE	-6.69	-10.11	-9.92	-9.65	-10.18
CDK2	3EZR	3EZR-EZR-300	3-METHOXY-4-{3-[4-(4-METHYLPIPERAZIN-1-YL)-1H-BENZIMIDAZOL-2-YL]-1H-INDAZOL-6-YL}ANILINE	-8.81	-9.29	-9.75	-9.72	-9.77
CDK2	3EZV	3EZV-EZV-300	4-{3-[7-(4-METHYLPIPERAZIN-1-YL)-1H-BENZIMIDAZOL-2-YL]-1H-INDAZOL-6-YL}ANILINE	-9.30	-8.23	-9.17	-9.72	-8.76
CDK2	3F5X	3F5X-EZV-300	4-{3-[7-(4-METHYLPIPERAZIN-1-YL)-1H-BENZIMIDAZOL-2-YL]-1H-INDAZOL-6-YL}ANILINE	-10.40	-9.84	-9.89	-9.82	-10.25
CDK2	3FZ1	3FZ1-B98-299	(3R)-3-(AMINOMETHYL)-9-METHOXY-1,2,3,4-TETRAHYDRO-5H-[1]BENZOTHIENO[3,2-E][1,4]DIAZEPIN-5-ONE	-5.87	-9.55	-9.42	-10.05	-10.14
CDK2	3IG7	3IG7-EFP-999	N-{1-[CIS-3-(ACETYLAMINO)CYCLOBUTYL]-1H-IMIDAZOL-4-YL}-2-(4-METHOXYPHENYL)ACETAMIDE	-5.80	-8.38	-8.06	-7.29	-9.54
CDK2	3IGG	3IGG-EFQ-999	N-[1-(CIS-3-HYDROXYCYCLOBUTYL)-1H-IMIDAZOL-4-YL]-2-(4-METHOXYPHENYL)ACETAMIDE	-6.71	-8.56	-9.35	-9.45	-10.54
CDK2	3LE6	3LE6-2BZ-299	5-(2-CHLOROPHENYL)-3-METHYL-7-NITROPYRAZOLO[3,4-B][1,4]BENZODIAZEPINE	-9.50	-9.67	-8.86	-9.66	-9.15
CDK2	3LFN	3LFN-A27-299	N-[6-(4-HYDROXYPHENYL)-5-PHENYL-1H-INDAZOL-3-YL]BUTANAMIDE	-10.17	-7.86	-7.92	-9.97	-8.33
CDK2	3LFQ	3LFQ-A28-299	N-(6,7-DIFLUORO-5-PHENYL-1H-INDAZOL-3-YL)BUTANAMIDE	-8.56	-8.96	-8.91	-10.10	-10.03
CDK2	3LFS	3LFS-A07-299	N-(6-CHLORO-5-PHENYL-1H-INDAZOL-3-YL)BUTANAMIDE	-8.02	-8.52	-8.90	-8.94	-9.70
CDK2	3NS9	3NS9-NS9-0	(2S,3S)-3-{[7-(BENZYLAMINO)-3-(1-METHYLETHYL)PYRAZOLO[1,5-A]PYRIMIDIN-5-YL]AMINO}BUTANE-1,2,4-TRIOL	-7.76	-9.05	-8.75	-9.39	-8.97
CDK2	3PXF	3PXF-2AN-304	8-ANILINO-1-NAPHTHALENE SULFONATE	-6.05	-8.29	-8.51	-8.50	-7.29
CDK2	3PXQ	3PXQ-2AN-300	8-ANILINO-1-NAPHTHALENE SULFONATE	-8.79	-6.11	-5.13	-4.72	-4.47
CDK2	3PXZ	3PXZ-JWS-301	2-(4,6-DIAMINO-1,3,5-TRIAZIN-2-YL)-4-METHOXYPHENOL	-7.53	-6.16	-4.45	-6.92	-5.44
CDK2	3QHR	3QHR-ADP-297	ADENOSINE-5'-DIPHOSPHATE	-7.52	-9.97	-11.94	-10.45	-11.91

CDK2	3QQF	3QQF-X07-543	5-NITRO-2-[(PYRIDIN-3-YLMETHYL)AMINO]BENZAMIDE	-6.93	-7.95	-9.11	-8.61	-9.75
CDK2	3QQH	3QQH-X0A-303	2-[4-AMINO-6-(PHENYLAMINO)-1,3,5-TRIAZIN-2-YL]-4-CHLOROPHENOL	-9.44	-5.57	-5.97	-6.33	-5.95
CDK2	3QQJ	3QQJ-X11-300	2-(4,6-DIAMINO-1,3,5-TRIAZIN-2-YL)PHENOL	-7.41	-7.47	-10.98	-7.25	-7.93
CDK2	3QTS	3QTS-X46-299	[4-AMINO-2-(PHENYLAMINO)-1,3-THIAZOL-5-YL](3-METHOXYPHENYL)METHANONE	-6.26	-8.95	-7.76	-8.54	-7.82
CDK2	3QTX	3QTX-X43-299	4-{[4-AMINO-5-(3-NITROBENZOYL)-1,3-THIAZOL-2-YL]AMINO}BENZENESULFONAMIDE	-6.41	-7.85	-8.14	-8.15	-7.97
CDK2	3QXO	3QXO-X65-668	5-NITRO-2-[(4-SULFAMOYLBENZYL)AMINO]BENZAMIDE	-7.26	-8.59	-9.34	-9.15	-9.52
CDK2	3QZF	3QZF-X66-299	2-(4,6-DIAMINO-1,3,5-TRIAZIN-2-YL)BENZENE-1,4-DIOL	-8.01	-7.00	-6.02	-6.60	-7.01
CDK2	3QZH	3QZH-X69-839	4-METHOXY-5-NITRO-2-[(PYRIDIN-3-YLMETHYL)AMINO]BENZAMIDE	-6.46	-10.22	-9.46	-9.87	-9.62
CDK2	3R7Y	3R7Y-Z04-484	2-{[(2-AMINOPYRIMIDIN-5-YL)METHYL]AMINO}-4-(MORPHOLIN-4-YL)-5-NITROBENZAMIDE	-7.88	-9.45	-9.68	-9.71	-9.10
CDK2	3R8L	3R8L-Z30-423	(5R)-5-TERT-BUTYL-4,5,6,7-TETRAHYDRO-1H-INDAZOLE-3-CARBOHYDRAZIDE	-6.39	-8.84	-8.37	-9.90	-8.07
CDK2	3R8Z	3R8Z-Z63-440	[4-AMINO-2-(PROP-2-EN-1-YLAMINO)-1,3-THIAZOL-5-YL](PYRIDIN-4-YL)METHANONE	-6.05	-7.82	-7.50	-7.44	-7.74
CDK2	3R9O	3R9O-Z71-401	4-AMINO-N-(3,5-DIFLUOROPHENYL)-2-[(4-SULFAMOYLPHENYL)AMINO]-1,3-THIAZOLE-5-CARBOXAMIDE	-7.47	-7.35	-11.39	-8.84	-10.08
CDK2	3RAL	3RAL-04Z-499	4-{[4-AMINO-5-(3-METHOXYBENZOYL)-1,3-THIAZOL-2-YL]AMINO}BENZENESULFONAMIDE	-8.34	-8.24	-9.76	-8.50	-9.75
CDK2	3RK5	3RK5-07Z-436	4-{[4-AMINO-5-(PYRIDIN-3-YLCARBONYL)-1,3-THIAZOL-2-YL]AMINO}BENZOIC ACID	-6.70	-7.71	-7.83	-8.64	-9.08
CDK2	3RK7	3RK7-08Z-467	4-{[4-AMINO-5-(PYRIDIN-3-YLCARBONYL)-1,3-THIAZOL-2-YL]AMINO}BENZAMIDE	-6.89	-7.54	-8.12	-8.94	-8.78
CDK2	3RMF	3RMF-20Z-496	4-{[4-AMINO-5-(NAPHTHALEN-2-YLCARBONYL)-1,3-THIAZOL-2-YL]AMINO}BENZENESULFONAMIDE	-10.17	-8.34	-10.81	-8.50	-9.63
CDK2	3RNI	3RNI-21Z-424	3-[(4-AMINO-5-BENZOYL-1,3-THIAZOL-2-YL)AMINO]BENZENESULFONAMIDE	-7.69	-9.73	-9.62	-10.53	-9.90
CDK2	3S0O	3S0O-50Z-446	[4-AMINO-2-(PROP-2-EN-1-YLAMINO)-1,3-THIAZOL-5-YL](PYRIDIN-2-YL)METHANONE	-6.01	-7.34	-8.60	-8.91	-8.79
CDK2	3S1H	3S1H-56Z-479	4-{[4-AMINO-5-(4-METHOXYBENZOYL)-1,3-THIAZOL-2-YL]AMINO}BENZENESULFONAMIDE	-8.04	-8.63	-10.48	-8.22	-10.03
CDK2	3S2P	3S2P-PMU-500	(3S,4S)-1-{3-[2-AMINO-6-(PROPAN-2-YL)PYRIMIDIN-4-YL]-4-HYDROXYPHENYL}PYRROLIDINE-3,4-DIOL	-5.45	-8.92	-10.54	-9.92	-10.02
CDK2	3TI1	3TI1-B49-299	N-[2-(DIETHYLAMINO)ETHYL]-5-[(Z)-(5-FLUORO-2-OXO-1,2-DIHYDRO-3H-INDOL-3-YLIDENE)METHYL]-2,4-DIMETHYL-1H-PYRROLE-3-CARBOXAMIDE	-6.90	-7.67	-6.82	-7.38	-7.65
CDK2	3TIZ	3TIZ-3TI-299	1-{(E)-[(4-HYDROXYPHENYL)IMINO]METHYL}NAPHTHALEN-2-OL	-6.58	-6.99	-6.89	-6.65	-6.23
CDK2	3UNJ	3UNJ-0BX-299	4-{[4-(PHENYLAMINO)PYRIMIDIN-2-YL]AMINO}BENZOIC ACID	-9.04	-6.42	-6.56	-6.72	-5.64
CDK2	3UNK	3UNK-0BY-299	4-({4-[(2-CHLOROPHENYL)AMINO]PYRIMIDIN-2-YL}AMINO)BENZOIC ACID	-7.90	-6.72	-6.84	-9.88	-8.41
CDK2	3WBL	3WBL-PDY-302	N~7~-(4-ETHOXYPHENYL)-6-METHYL-N~5~-[(3S)-PIPERIDIN-3-YL]PYRAZOLO[1,5-A]PYRIMIDINE-5,7-DIAMINE	-7.31	-8.88	-8.75	-8.21	-8.90
CDK2	4BCK	4BCK-T3E-1295	3-[[5-CYANO-4-[4-METHYL-2-(METHYLAMINO)-1,3-THIAZOL-5-YL]PYRIMIDIN-2-YL]AMINO]BENZENESULFONAMIDE	-5.98	-8.97	-9.00	-8.84	-9.36
CDK2	4BCM	4BCM-T7Z-1295	4-(4-METHYL-2-METHYLIMINO-3H-1,3-THIAZOL-5-YL)-2-[(4-METHYL-3-MORPHOLIN-4-YLSULFONYL-PHENYL)AMINO]PYRIMIDINE-5-CARBONITRILE	-7.55	-9.48	-9.32	-9.45	-9.11
CDK2	4BGH	4BGH-3I6-1001	4-((5-BROMO-4-(PROP-2-YN-1-YLAMINO)PYRIMIDIN-2-YL)AMINO)BENZENESULFONAMIDE	-6.89	-7.97	-7.65	-9.17	-7.79
CDK2	4CFM	4CFM-4QE-1297	6-(CYCLOHEXYLMETHOXY)-8-(2-METHYLPHENYL)-9H-PURIN-2-AMINE	-7.68	-9.35	-9.44	-9.95	-9.98
CDK2	4D1X	4D1X-ESJ-1299	(4S)-2-(6-HYDROXY-1,3-BENZOTHIAZOL-2-YL)-4,5-DIHYDRO-1,3-THIAZOLE-4-CARBOXYLIC ACID	-6.52	-6.53	-4.98	-7.06	-4.75

CDK2	4D1Z	4D1Z-WG8-1296	(4S)-2-(8-HYDROXYQUINOLIN-2-YL)-4,5-DIHYDRO-1,3-THIAZOLE-4-CARBOXYLIC ACID	-8.63	-7.83	-8.66	-8.46	-7.49
CDK2	4EOI	4EOI-1RO-301	(5E)-5-(QUINOLIN-6-YLMETHYLIDENE)-2-[(THIOPHEN-2-YLMETHYL)AMINO]-1,3-THIAZOL-4(5H)-ONE	-7.91	-8.89	-8.92	-8.58	-9.34
CDK2	4EOK	4EOK-4SP-301	O6-CYCLOHEXYLMETHOXY-2-(4'-SULPHAMOYLANILINO) PURINE	-8.34	-7.96	-8.20	-9.03	-10.08
CDK2	4EOP	4EOP-1RO-301	(5E)-5-(QUINOLIN-6-YLMETHYLIDENE)-2-[(THIOPHEN-2-YLMETHYL)AMINO]-1,3-THIAZOL-4(5H)-ONE	-7.99	-6.71	-6.35	-7.37	-7.62
CDK2	4ERW	4ERW-STU-301	STAUROSPORINE	-11.45	-9.04	-8.65	-9.45	-9.32
CDK2	4EZ3	4EZ3-0S0-301	4-[(E)-(6-HYDROXY-2-OXO-1,2-DIHYDROPYRIDIN-3-YL)DIAZENYL]BENZENESULFONAMIDE	-6.20	-8.39	-9.13	-8.16	-9.25
CDK2	4EZ7	4EZ7-2AN-302	8-ANILINO-1-NAPHTHALENE SULFONATE	-8.98	-8.64	-9.69	-7.60	-9.53
CDK2	4FKS	4FKS-46K-301	N-[(4-{[(Z)-(7-OXO-6,7-DIHYDRO-8H-[1,3]THIAZOLO[5,4-E]INDOL-8-YLIDENE)METHYL]AMINO}PHENYL)SULFONYL]ACETAMIDE	-10.67	-8.51	-8.88	-9.79	-8.73
CDK2	4FKT	4FKT-48K-301	N-[2-(DIMETHYLAMINO)ETHYL]-4-{[(Z)-(2-OXO-1,2-DIHYDRO-3H-INDOL-3-YLIDENE)METHYL]AMINO}BENZENESULFONAMIDE	-7.88	-8.41	-9.03	-10.64	-8.89
CDK2	4FKU	4FKU-60K-301	(3Z)-2-OXO-3-[2-(4-SULFAMOYLPHENYL)HYDRAZINYLIDENE]-2,3-DIHYDRO-1H-INDOLE-5-CARBOXYLIC ACID	-2.83	-6.26	-5.04	-5.54	-4.95
CDK2	4FX3	4FX3-60K-301	(3Z)-2-OXO-3-[2-(4-SULFAMOYLPHENYL)HYDRAZINYLIDENE]-2,3-DIHYDRO-1H-INDOLE-5-CARBOXYLIC ACID	-9.55	-8.11	-8.42	-8.63	-9.63
CDK2	4I3Z	4I3Z-ADP-301	ADENOSINE-5'-DIPHOSPHATE	-6.52	-9.98	-10.56	-9.89	-10.40
CDK2	4II5	4II5-ADP-301	ADENOSINE-5'-DIPHOSPHATE	-6.55	-8.96	-10.51	-9.49	-10.52
CDK2	4KD1	4KD1-1QK-302	3-[({3-ETHYL-5-[(2S)-2-(2-HYDROXYETHYL)PIPERIDIN-1-YL]PYRAZOLO[1,5-A]PYRIMIDIN-7-YL}AMINO)METHYL]-1-HYDROXYPYRIDINIUM	-8.26	-6.72	-7.74	-7.04	-6.53
CDK2	4LYN	4LYN-1YG-301	(2S)-N-(5-{[(5-TERT-BUTYL-1,3-OXAZOL-2-YL)METHYL]SULFANYL}-1,3-THIAZOL-2-YL)-2-PHENYLPROPANAMIDE	-7.12	-9.45	-8.93	-9.52	-8.72
CDK2	4RJ3	4RJ3-3QS-302	1-CYCLOPENTYL-N-[2-(4-METHOXYPIPERIDIN-1-YL)PYRIMIDIN-4-YL]-1H-PYRROLO[3,2-C]PYRIDIN-6-AMINE	-8.95	-9.87	-10.17	-10.21	-10.11
CDK2	5A14	5A14-LQ5-1297	1-[4-(2-AZANYLPYRIMIDIN-4-YL)OXYPHENYL]-3-[4-[(4-METHYLPIPERAZIN-1-YL)METHYL]-3-(TRIFLUOROMETHYL)PHENYL]UREA	-11.78	-6.60	-5.63	-6.26	-7.38
CDK2	5ANG	5ANG-WY3-1299	7-HYDROXY-4-(MORPHOLINOMETHYL)CHROMEN-2-ONE	-7.14	-9.55	-9.16	-10.25	-9.12
CDK2	5ANJ	5ANJ-ZXC-1299	N-(9H-PURIN-6-YL)THIOPHENE-2-CARBOXAMIDE	-5.16	-8.70	-7.82	-8.81	-7.81
CDK2	5ANK	5ANK-RJI-1299	2,4,6-TRIOXO-1-PHENYL-HEXAHYDROPYRIMIDINE-5-CARBOXAMIDE	-7.71	-6.45	-8.18	-7.22	-7.98
CDK2	5CYI	5CYI-55S-301	6-(CYCLOHEXYLMETHOXY)-N-[4-(ETHYLSULFONYL)PHENYL]-9H-PURIN-2-AMINE	-3.19	-8.53	-8.82	-8.83	-10.18
CDK2	5D1J	5D1J-56H-4000	N-(5-{[(5-TERT-BUTYL-1,3-OXAZOL-2-YL)METHYL]SULFANYL}-1,3-THIAZOL-2-YL)PIPERIDINE-4-CARBOXAMIDE	-7.47	-9.50	-9.12	-9.14	-9.53
CDK2	5FP6	5FP6-MFZ-1295	3-(4,7-DICHLORO-1H-INDOL-3-YL)PROP-2-YN-1-OL	-6.98	-6.81	-7.25	-8.95	-8.37
CDK2	5JQ5	5JQ5-I74-302	(3R,4R)-4-[[[7-[(PHENYLMETHYL)AMINO]-3-PROPAN-2-YL-PYRAZOLO[1,5-A]PYRIMIDIN-5-YL]AMINO]METHYL]PIPERIDIN-3-OL	-8.63	-9.51	-9.49	-9.95	-9.93
CDK2	5JQ8	5JQ8-I73-301	(3S,4S)-4-[[[7-[(PHENYLMETHYL)AMINO]-3-PROPAN-2-YL-PYRAZOLO[1,5-A]PYRIMIDIN-5-YL]AMINO]METHYL]PIPERIDIN-3-OL	-7.12	-8.65	-7.89	-9.79	-8.10
CDK2	5L2W	5L2W-1QK-900	3-[({3-ETHYL-5-[(2S)-2-(2-HYDROXYETHYL)PIPERIDIN-1-YL]PYRAZOLO[1,5-A]PYRIMIDIN-7-YL}AMINO)METHYL]-1-HYDROXYPYRIDINIUM	-8.51	-6.91	-8.70	-7.06	-9.44
CDK2	5NEV	5NEV-72L-301	4-[[6-(3-PHENYLPHENYL)-7~{H}-PURIN-2-YL]AMINO]BENZENESULFONAMIDE	-8.04	-9.44	-9.47	-9.21	-9.70
CDK2	5OO0	5OO0-9YZ-301	METHYL 4-PROPANOYL-2,3-DIHYDROQUINOXALINE-1-CARBOXYLATE	1.84	-6.57	-7.06	-6.35	-6.80
CDK2	5OO1	5OO1-9Z2-301	~{N}-(4-PYRIMIDIN-2-YLPHENYL)PROPANAMIDE	0.42	-7.88	-8.76	-8.71	-9.58

CDK2	5OO3	5OO3-9ZB-301	1-(4-ETHYL-2,3-DIHYDROQUINOXALIN-1-YL)PROPAN-1-ONE	-1.72	-6.43	-8.64	-6.91	-8.26
CDK2	6GUB	6GUB-F9Z-301	2-(2-CHLOROPHENYL)-8-[(3~{R},4~{R})-1-METHYL-3-OXIDANYL-PIPERIDIN-4-YL]-5,7-BIS(OXIDANYL)CHROMEN-4-ONE	-9.39	-8.34	-8.60	-9.39	-10.19
CDK2	6GUC	6GUC-SU9-301	(3Z)-3-(1H-IMIDAZOL-5-YLMETHYLENE)-5-METHOXY-1H-INDOL-2(3H)-ONE	-8.35	-7.92	-8.33	-8.58	-9.04
CDK2	6GUE	6GUE-FB8-301	4-(2-METHYL-3-PROPAN-2-YL-IMIDAZOL-4-YL)-~{N}-(4-METHYLSULFONYLPHENYL)PYRIMIDIN-2-AMINE	-8.68	-8.94	-9.30	-8.62	-9.18
CDK2	6GUF	6GUF-23D-301	N2-[(1R,2S)-2-AMINOCYCLOHEXYL]-N6-(3-CHLOROPHENYL)-9-ETHYL-9H-PURINE-2,6-DIAMINE	-8.37	-8.92	-9.81	-8.99	-10.33
CDK2	6GUK	6GUK-FC8-301	~{N}2-[(1~{R},2~{S})-2-AZANYLCYCLOHEXYL]-~{N}6-(3-CHLOROPHENYL)-9-ETHYL-PURINE-2,6-DIAMINE	-8.79	-9.94	-9.31	-9.78	-9.55
CDK2	6GVA	6GVA-FCQ-301	5-(2-AZANYLETHYLSULFANYL)-3-PROPAN-2-YL-~{N}-[(4-PYRIDIN-2-YLPHENYL)METHYL]-2~{H}-PYRAZOLO[4,3-D]PYRIMIDIN-7-AMINE	-8.38	-8.96	-8.40	-9.07	-9.55
CDK2	6INL	6INL-AJR-900	2,2'-{[6-{[(4-METHOXYPHENYL)METHYL]AMINO}-9-(PROPAN-2-YL)-9H-PURIN-2-YL]AZANEDIYL}DI(ETHAN-1-OL)	-7.29	-8.41	-9.27	-8.61	-9.42
CDK2	6JGM	6JGM-AQG-900	4-{[6-(CYCLOHEXYLMETHOXY)-7H-PURIN-2-YL]AMINO}-N,N-DIETHYLBENZAMIDE	-7.78	-8.31	-9.34	-8.68	-9.42
CDK2	6OQI	6OQI-N14-301	5-FLUORO-N-[5-(4-METHYLPIPERAZIN-1-YL)PYRIDIN-2-YL]-4-[(4S)-4-METHYL-5,6,7,8-TETRAHYDRO-4H-PYRAZOLO[1,5-A]AZEPIN-3-YL]PYRIMIDIN-2-AMINE	-8.80	-9.29	-8.25	-10.00	-9.19
CDK2	7KJS	7KJS-WG1-301	6-(DIFLUOROMETHYL)-8-[(1R,2R)-2-HYDROXY-2-METHYLCYCLOPENTYL]-2-{[1-(METHYLSULFONYL)PIPERIDIN-4-YL]AMINO}PYRIDO[2,3-D]PYRIMIDIN-7(8H)-ONE	-10.09	-9.59	-9.05	-9.68	-9.63
CDK2	7MKX	7MKX-ZGY-301	2-[(5-BROMO-2-{4-[(CYANOMETHYL)SULFAMOYL]ANILINO}PYRIMIDIN-4-YL)AMINO]-6-FLUOROBENZAMIDE	-6.98	-8.20	-9.53	-9.02	-9.25
CDK2	7NVQ	7NVQ-STU-301	STAUROSPORINE	-13.63	-9.53	-8.89	-9.86	-9.27
CDK2	7RWE	7RWE-7TH-301	5-NITRO-2-[(3-PHENYLPROPYL)AMINO]BENZOIC ACID	-6.88	-7.55	-7.67	-7.90	-7.46
CDK2	7RWF	7RWF-7TW-302	2-{[2-(1H-INDOL-3-YL)ETHYL]AMINO}-5-NITROBENZOIC ACID	-10.68	3.37	1.78	-0.23	0.02
CDK2	7S4T	7S4T-88O-302	2-{[2-(6-CHLORO-1H-INDOL-3-YL)ETHYL]AMINO}-5-NITROBENZOIC ACID	-10.84	6.84	-3.74	-0.68	3.55
CDK2	7S84	7S84-8IL-301	2-{[2-(1H-INDOL-3-YL)ETHYL]AMINO}-5-(TRIFLUOROMETHYL)BENZOIC ACID	-11.10	6.31	05.03	1.88	5.16
CDK2	7S85	7S85-8IQ-303	2-{[2-(1H-INDOL-3-YL)ETHYL]AMINO}-5-(TRIFLUOROMETHOXY)BENZOIC ACID	-10.69	-1.97	-1.79	-2.88	-0.08
CDK2	7SA0	7SA0-8KQ-305	2-{[(3R)-2,3,4,9-TETRAHYDRO-1H-CARBAZOL-3-YL]AMINO}-5-(TRIFLUOROMETHYL)BENZOIC ACID	-9.59	-8.05	-7.56	-7.65	-7.85
CDK2	7UG1	7UG1-N5R-302	8-(4-CHLOROANILINO)NAPHTHALENE-1-SULFONIC ACID	-5.68	-8.02	-8.50	-8.00	-7.61
CDK2	8B54	8B54-P2V-301	~{N}-[(2-PHENYLPHENYL)METHYL]-5-PIPERIDIN-4-YLSULFANYL-3-PROPAN-2-YL-2~{H}-PYRAZOLO[4,3-D]PYRIMIDIN-7-AMINE	-9.10	-9.18	-9.11	-9.12	-9.19
CDK2	8ERD	8ERD-WQ6-301	(2-{[1-(METHANESULFONYL)PIPERIDIN-4-YL]AMINO}QUINAZOLIN-7-YL)[(2S)-2-METHYLPYRROLIDIN-1-YL]METHANONE	-10.19	-9.94	-10.22	-9.45	-9.30
CDK2	8ERN	8ERN-WQK-402	N,3-DIMETHYL-4-({7-[(2S)-2-METHYLPYRROLIDINE-1-CARBONYL]QUINAZOLIN-2-YL}AMINO)BENZENE-1-SULFONAMIDE	-10.61	-9.57	-9.86	-9.42	-9.24
CDK2	8FP0	8FP0-7TW-304	2-{[2-(1H-INDOL-3-YL)ETHYL]AMINO}-5-NITROBENZOIC ACID	-7.65	10.03	1.76	1.42	6.40
CDK2	8FP5	8FP5-ATP-302	ADENOSINE-5'-TRIPHOSPHATE	-7.59	-9.11	-8.13	-9.56	-9.17
CDK2	8H6P	8H6P-WZU-301	(7S,10R)-11-OXA-2,4,5,13,17,23-HEXAAZATETRACYCLO[17.3.1.1~3,6~.1~7,10~]PENTACOSA-1(23),3(25),5,19,21-PENTAENE-12,18-DIONE	-11.79	-9.02	-8.63	-9.76	-8.66
CDK2	8H6T	8H6T-WZZ-301	(1R,3S)-3-{3-[(PYRIDIN-2-YL)AMINO]-1H-PYRAZOL-5-YL}CYCLOPENTYL PROPAN-2-YLCARBAMATE	-8.18	-8.79	-9.55	-9.36	-9.91

CDK2	8OY2	8OY2-W5W-301	(1S,2S,11AS)-1-METHOXY-1,4,7,10-TETRAMETHYL-2,9-BIS(OXIDANYL)-2,11A-DIHYDROBENZO[B][1,4]BENZODIOXEPINE-3,6-DIONE	-8.32	-9.48	-9.77	-9.15	-9.80
CDK2	8UV0	8UV0-XKU-301	1-{(4M)-4-[2-{[1-(CYCLOPROPANESULFONYL)PIPERIDIN-4-YL]AMINO}-5-(TRIFLUOROMETHYL)PYRIMIDIN-4-YL]-1H-PYRAZOL-1-YL}-2-METHYLPROPAN-2-OL	-9.31	-7.96	-8.54	-8.99	-8.75

Table S3. Individual AutoDockVina scores for the ligands docked to the predicted target protein CDK4. PDB ID—identificator of PDB structure of the target protein with co-crystallized ligand (CCL). CCL ID—combination of PDB ID, residue name, and residue number that identifies CCL structure in the source PDB file. R, S—enantiomers of Les-6547 and Les-6557, respectively. Values in the "CCL" column represent individual Vina scores for different CCL bound to a different conformation of the predicted target, evaluated by AutoDock Vina in score-only mode without performing docking. The remaining four columns contain individual Vina docking scores of Les-6547 and Les-6557 enantiomers docked to the same conformations of the target that bind CCLs. For each of n

Target Protein	PDB ID	CCL ID	CCL Name	Vina scores, kkal/mol				
				CCL	Les-6547		Les-6557	
					R	S	R	S
CDK4	5FWK	5FWK-ATP-725	ADENOSINE-5'-TRIPHOSPHATE	-9.05	-9.28	-10.60	-8.79	-9.97
CDK4	5FWL	5FWL-ATP-1692	ADENOSINE-5'-TRIPHOSPHATE	-9.04	-8.63	-10.72	-7.63	-9.57
CDK4	5FWM	5FWM-ATP-1691	ADENOSINE-5'-TRIPHOSPHATE	-10.35	-7.48	-6.95	-7.51	-8.28
CDK4	5FWP	5FWP-ATP-1691	ADENOSINE-5'-TRIPHOSPHATE	-10.45	-7.01	-6.70	-7.23	-8.09
CDK4	7SJ3	7SJ3-6ZV-401	N-{5-[(4-ETHYLPIPERAZIN-1-YL)METHYL]PYRIDIN-2-YL}-5-FLUORO-4-[4-FLUORO-2-METHYL-1-(PROPAN-2-YL)-1H-BENZIMIDAZOL-6-YL]PYRIMIDIN-2-AMINE	-9.80	-7.93	-8.88	-9.25	-10.10

target conformations, the most favorable docking score was selected. The CCLs were removed from the protein structures as the latter were prepared for docking.

Table S4. Individual AutoDockVina scores for the ligands docked to the predicted target protein JAK2. PDB ID—identificator of PDB structure of the target protein with co-crystallized ligand (CCL). CCL ID—combination of PDB ID, residue name, and residue number that identifies CCL structure in the source PDB file. R, S—enantiomers of Les-6547 and Les-6557, respectively. Values in the "CCL" column represent individual Vina scores for different CCLs bound to a different conformation of the predicted target, evaluated by AutoDock Vina in score-only mode without performing docking. The remaining four columns contain individual Vina docking scores of Les-6547 and Les-6557 enantiomers docked to the same conformations of the target that bind CCLs. For each of n target conformations, the most favorable docking score was selected. The CCLs were removed from the protein structures as the latter were prepared for docking.

Target Protein	PDB ID	CCL ID	CCL Name	Vina scores, kkal/mol				
				CCL	Les-6547		Les-6557	
					R	S	R	S
JAK2	2B7A	2B7A-IZA-2002	2-TERT-BUTYL-9-FLUORO-3,6-DIHYDRO-7H-BENZ[H]-IMIDAZ[4,5-F]ISOQUINOLINE-7-ONE	-10.98	-8.51	-9.59	-9.40	-9.69
JAK2	2W1I	2W1I-L0I-2133	4-[(2-{4-[(CYCLOPROPYLCARBAMOYL)AMINO]-1H-PYRAZOL-3-YL}-1H-BENZIMIDAZOL-6-YL)METHYL]MORPHOLIN-4-IUM	-7.05	-8.33	-8.40	-9.08	-8.08
JAK2	2XA4	2XA4-AZ5-2131	5-CHLORO-N2-[(1S)-1-(5-FLUOROPYRIMIDIN-2-YL)ETHYL]-N4-(5-METHYL-1H-PYRAZOL-3-YL)PYRIMIDINE-2,4-DIAMINE	-7.73	-8.88	-8.70	-9.09	-8.58
JAK2	3FUP	3FUP-MI1-1	3-{(3R,4R)-4-METHYL-3-[METHYL(7H-PYRROLO[2,3-D]PYRIMIDIN-4-YL)AMINO]PIPERIDIN-1-YL}-3-OXOPROPANENITRILE	-6.53	-9.46	-8.53	-8.67	-9.27
JAK2	3IO7	3IO7-1P5-1	(3S)-1-[6-(2-AMINOPYRAZOLO[1,5-A]PYRIMIDIN-3-YL)PYRIMIDIN-4-YL]-N,N-DIETHYLPIPERIDINE-3-CARBOXAMIDE	-7.85	-8.22	-9.19	-8.71	-9.60
JAK2	3JY9	3JY9-JZH-1	(3S)-3-(4-HYDROXYPHENYL)-1,5-DIHYDRO-1,5,12-TRIAZABENZO[4,5]CYCLOOCTA[1,2,3-CD]INDEN-4(3H)-ONE	-11.16	-8.98	-8.91	-8.97	-8.94
JAK2	3KCK	3KCK-3KC-1	3-CHLORO-4-(4H-3,4,7-TRIAZADIBENZO[CD,F]AZULEN-6-YL)PHENOL	-9.13	-9.21	-9.40	-9.25	-9.58
JAK2	3LPB	3LPB-NVB-1133	N-METHYL-4-[3-(3,4,5-TRIMETHOXYPHENYL)QUINOXALIN-5-YL]BENZENESULFONAMIDE	-9.33	-10.17	-10.76	-9.77	-10.69
JAK2	3Q32	3Q32-J2I-1	2-(2,6-DIFLUORO-4-METHOXYPHENYL)-1-(4-{4-[(3-METHYL-1H-PYRAZOL-5-YL)AMINO]PYRROLO[2,1-F][1,2,4]TRIAZIN-2-YL}PIPERAZIN-1-YL)ETHANONE	-9.48	-8.84	-10.95	-8.99	-10.14
JAK2	3RVG	3RVG-17P-2000	1-(CYCLOHEXYLAMINO)-7-(1-METHYL-1H-PYRAZOL-4-YL)-5H-PYRIDO[4,3-B]INDOLE-4-CARBOXAMIDE	-7.04	-8.30	-9.08	-9.56	-9.80
JAK2	3TJC	3TJC-0TP-1	4-AMINO-N-METHYL-2-[4-(MORPHOLIN-4-YL)PHENYL]THIENO[3,2-C]PYRIDINE-7-CARBOXAMIDE	-7.00	-9.39	-9.65	-8.92	-9.57
JAK2	3TJD	3TJD-6TP-1	4-AMINO-2-[4-(TERT-BUTYLSULFAMOYL)PHENYL]-N-METHYLTHIENO[3,2-C]PYRIDINE-7-CARBOXAMIDE	-6.81	-9.63	-9.67	-9.46	-9.62
JAK2	4BBE	4BBE-3O4-2229	N-[4-[2-[(4-MORPHOLIN-4-YLPHENYL)AMINO]PYRIMIDIN-4-YL]PHENYL]ETHANAMIDE	-8.98	-9.63	-9.91	-9.65	-9.95
JAK2	4BBF	4BBF-O19-2229	(2R)-N-[4-[2-[(4-MORPHOLIN-4-YLPHENYL)AMINO]PYRIMIDIN-4-YL]PHENYL]PYRROLIDINE-2-CARBOXAMIDE	-9.49	-9.09	-8.96	-8.98	-8.76
JAK2	4C61	4C61-LMM-2133	N2-[(1S)-1-(5-FLUOROPYRIMIDIN-2-YL)ETHYL]-7-METHYL-N4-(1-METHYLIMIDAZOL-4-YL)THIENO[3,2-D]PYRIMIDINE-2,4-DIAMINE	-6.94	-8.91	-9.96	-8.97	-9.87
JAK2	4C62	4C62-XWW-2133	N2-[(1S)-1-(5-FLUOROPYRIMIDIN-2-YL)ETHYL]-N4-(1-METHYLIMIDAZOL-4-YL)-6-MORPHOLINO-1,3,5-TRIAZINE-2,4-DIAMINE	-6.74	-8.92	-9.72	-9.03	-9.40
JAK2	4E4M	4E4M-0NH-1201	1-[4-METHYL-1-(METHYLSULFONYL)PIPERIDIN-4-YL]-1,6-DIHYDROIMIDAZO[4,5-D]PYRROLO[2,3-B]PYRIDINE	-7.95	-7.52	-8.86	-10.56	-8.44
JAK2	4E6Q	4E6Q-0NV-1201	1-(1-BENZYLPIPERIDIN-4-YL)-1,6-DIHYDROIMIDAZO[4,5-D]PYRROLO[2,3-B]PYRIDINE	-8.80	-9.18	-11.46	-8.80	-11.17
JAK2	4F08	4F08-1RS-1201	1-(PIPERIDIN-4-YL)-1,6-DIHYDROIMIDAZO[4,5-D]PYRROLO[2,3-B]PYRIDINE	-7.56	-9.02	-9.86	-9.13	-9.75

JAK2	4F09	4F09-JAK-1201	2-METHYL-1-(PIPERIDIN-4-YL)-1,6-DIHYDROIMIDAZO[4,5-D]PYRROLO[2,3-B]PYRIDINE	-7.72	-8.84	-9.60	-8.55	-9.35
JAK2	4FVQ	4FVQ-ATP-901	ADENOSINE-5'-TRIPHOSPHATE	-7.51	-9.13	-10.11	-9.15	-10.52
JAK2	4FVR	4FVR-ATP-901	ADENOSINE-5'-TRIPHOSPHATE	-7.75	-8.95	-9.94	-9.85	-10.44
JAK2	4GMY	4GMY-0X5-1201	2,6-DICHLORO-N-{2-[(CYCLOPROPYLCARBONYL)AMINO]PYRIDIN-4-YL}BENZAMIDE	-6.78	-9.61	-9.39	-9.24	-9.52
JAK2	4JI9	4JI9-1M3-1201	N-TERT-BUTYL-3-[(5-METHYL-2-{[4-(4-METHYLPIPERAZIN-1-YL)PHENYL]AMINO}PYRIMIDIN-4-YL)AMINO]BENZENESULFONAMIDE	-8.68	-10.13	-10.71	-10.17	-10.52
JAK2	4P7E	4P7E-2HB-1201	N-(5-{4-[(1,1-DIOXIDOTHIOMORPHOLIN-4-YL)METHYL]PHENYL}[1,2,4]TRIAZOLO[1,5-A]PYRIDIN-2-YL)CYCLOPROPANECARBOXAMIDE	-8.77	-9.04	-10.40	-9.71	-10.15
JAK2	4YTC	4YTC-4HW-4000	N~3~-PHENYL-1-[6-(PHENYLAMINO)PYRIMIDIN-4-YL]-1H-1,2,4-TRIAZOLE-3,5-DIAMINE	-8.40	-8.50	-8.66	-9.15	-8.71
JAK2	4YTF	4YTF-4HZ-4000	N~2~-[2-(5-CHLORO-1H-PYRROLO[2,3-B]PYRIDIN-3-YL)-5-FLUOROPYRIMIDIN-4-YL]-N-(2,2,2-TRIFLUOROETHYL)-L-ALANINAMIDE	-8.08	-8.82	-10.12	-9.30	-10.15
JAK2	4YTH	4YTH-467-4000	N~2~-[2-(5-CHLORO-1H-PYRROLO[2,3-B]PYRIDIN-3-YL)-5-FLUOROPYRIMIDIN-4-YL]-2-METHYL-N-(2,2,2-TRIFLUOROETHYL)-D-ALANINAMIDE	-8.20	-9.22	-9.17	-9.20	-9.47
JAK2	4YTI	4YTI-VJK-4000	(2R)-2-METHYL-2-[[2-(1H-PYRROLO[2,3-B]PYRIDIN-3-YL)PYRIMIDIN-4-YL]AMINO]-N-[2,2,2-TRIS(FLUORANYL)ETHYL]BUTANAMIDE	-8.16	-10.20	-10.75	-9.73	-10.32
JAK2	5CF4	5CF4-50Y-4000	N,N-DICYCLOPROPYL-6-ETHYL-4-[(3-METHOXYPROPYL)AMINO]-1-METHYL-1,6-DIHYDROIMIDAZO[4,5-D]PYRROLO[2,3-B]PYRIDINE-7-CARBOXAMIDE	-6.68	-9.28	-10.57	-9.60	-10.49
JAK2	5CF5	5CF5-50W-4000	N,N-DICYCLOPROPYL-4-[(4,5-DIMETHYL-1,3-THIAZOL-2-YL)AMINO]-6-ETHYL-1-METHYL-1,6-DIHYDROIMIDAZO[4,5-D]PYRROLO[2,3-B]PYRIDINE-7-CARBOXAMIDE	-8.89	-9.17	-10.33	-9.24	-10.33
JAK2	5CF6	5CF6-50O-4000	N,N-DICYCLOPROPYL-6-[(2S)-2,3-DIHYDROXYPROPYL]-1-METHYL-4-(METHYLAMINO)-1,6-DIHYDROIMIDAZO[4,5-D]PYRROLO[2,3-B]PYRIDINE-7-CARBOXAMIDE	-6.76	-9.59	-10.25	-9.32	-10.56
JAK2	5CF8	5CF8-50V-4000	N,N-DICYCLOPROPYL-4-[(1,5-DIMETHYL-1H-PYRAZOL-3-YL)AMINO]-6-ETHYL-1-METHYL-1,6-DIHYDROIMIDAZO[4,5-D]PYRROLO[2,3-B]PYRIDINE-7-CARBOXAMIDE	-8.39	-9.38	-10.24	-9.18	-10.18
JAK2	5HEZ	5HEZ-1M3-1201	N-TERT-BUTYL-3-[(5-METHYL-2-{[4-(4-METHYLPIPERAZIN-1-YL)PHENYL]AMINO}PYRIMIDIN-4-YL)AMINO]BENZENESULFONAMIDE	-8.41	-10.10	-10.89	-10.18	-10.65
JAK2	5I4N	5I4N-ATP-1000	ADENOSINE-5'-TRIPHOSPHATE	-8.17	-9.47	-9.48	-9.81	-10.28
JAK2	5L3A	5L3A-6DP-1201	~{N}-(1~{H}-INDAZOL-4-YL)METHANESULFONAMIDE	-5.57	-9.13	-10.33	-9.46	-10.22
JAK2	5TQ4	5TQ4-7GY-4000	6-(2-ETHYL-4-HYDROXYPHENYL)-1H-INDAZOLE-3-CARBOXAMIDE	-6.99	-8.99	-8.90	-8.51	-8.99
JAK2	5TQ5	5TQ5-7GX-4000	6-(2-ETHYL-4-HYDROXYPHENYL)-N-(6-METHYLPYRIDIN-3-YL)-1H-INDAZOLE-3-CARBOXAMIDE	-9.99	-8.84	-9.66	-9.31	-9.66
JAK2	5TQ6	5TQ6-7GV-4000	{(3R,4R)-4-METHYL-3-[METHYL(7H-PYRROLO[2,3-D]PYRIMIDIN-4-YL)AMINO]PIPERIDIN-1-YL}(PYRROLIDIN-1-YL)METHANONE	-8.48	-8.88	-9.58	-9.01	-9.23
JAK2	5TQ7	5TQ7-7GT-4000	{(3R,4R)-4-METHYL-3-[METHYL(7H-PYRROLO[2,3-D]PYRIMIDIN-	-8.95	-9.09	-10.18	-8.83	-10.00

			4-YL)AMINO]PIPERIDIN-1-YL}[(3R)-3-(PHENYLSULFONYL)PYRROLIDIN-1-YL]METHANONE					
JAK2	5TQ8	5TQ8-7GS-4000	{2-[6-(2-ETHYL-5-FLUORO-4-HYDROXYPHENYL)-2H-INDAZOL-3-YL]-3,4,6,7-TETRAHYDRO-5H-IMIDAZO[4,5-C]PYRIDIN-5-YL}[5-(PIPERIDIN-1-YL)PYRAZIN-2-YL]METHANONE	-12.42	-9.51	-9.47	-8.94	-9.43
JAK2	5USY	5USY-SKE-1201	4-({5-AMINO-1-[(2,6-DIFLUOROPHENYL)CARBONYL]-1H-1,2,4-TRIAZOL-3-YL}AMINO)BENZENESULFONAMIDE	-7.59	-8.57	-9.38	-9.86	-9.28
JAK2	5UT0	5UT0-35R-903	1-CYCLOPROPYL-3-{3-[5-(MORPHOLIN-4-YLMETHYL)-1H-BENZIMIDAZOL-2-YL]-1H-PYRAZOL-4-YL}UREA	-7.26	-8.75	-7.82	-9.36	-8.04
JAK2	5UT1	5UT1-7DZ-905	(7S)-2-[(3,5-DIFLUORO-4-HYDROXYPHENYL)AMINO]-5,7-DIMETHYL-8-(3-METHYLBUTYL)-7,8-DIHYDROPTERIDIN-6(5H)-ONE	-6.42	-9.09	-8.23	-9.11	-8.85
JAK2	5UT2	5UT2-3YT-903	2-{[(1R,2S)-2-AMINOCYCLOHEXYL]AMINO}-4-{[3-(2H-1,2,3-TRIAZOL-2-YL)PHENYL]AMINO}PYRIMIDINE-5-CARBOXAMIDE	-7.75	-9.74	-9.85	-9.76	-9.81
JAK2	5UT3	5UT3-IK1-901	5-PHENYL-2-UREIDOTHIOPHENE-3-CARBOXAMIDE	-6.13	-9.92	-8.73	-9.80	-9.82
JAK2	5UT4	5UT4-DQX-901	8-[3,5-DIFLUORO-4-(MORPHOLIN-4-YLMETHYL)PHENYL]-2-(1-PIPERIDIN-4-YL-1H-PYRAZOL-4-YL)QUINOXALINE	-9.53	-9.45	-9.71	-10.06	-9.44
JAK2	5WEV	5WEV-9ZS-1201	N-[2-(2,6-DICHLOROPHENYL)-1H-IMIDAZO[4,5-C]PYRIDIN-4-YL]CYCLOPROPANECARBOXAMIDE	-8.00	-9.56	-9.62	-9.85	-9.93
JAK2	5WIJ	5WIJ-AQG-901	4-{[6-(CYCLOHEXYLMETHOXY)-7H-PURIN-2-YL]AMINO}-N,N-DIETHYLBENZAMIDE	-5.71	-9.21	-9.07	-8.93	-8.81
JAK2	5WIK	5WIK-584-901	(7R)-2-[(3,5-DIFLUORO-4-HYDROXYPHENYL)AMINO]-5,7-DIMETHYL-8-(3-METHYLBUTYL)-7,8-DIHYDROPTERIDIN-6(5H)-ONE	-7.29	-9.18	-9.48	-9.72	-8.10
JAK2	5WIL	5WIL-YDJ-901	5-(3-FLUOROPHENYL)-N-[(3S)-3-PIPERIDYL]-3-UREIDO-THIOPHENE-2-CARBOXAMIDE	-6.87	-8.99	-9.39	-9.45	-10.38
JAK2	5WIM	5WIM-35R-901	1-CYCLOPROPYL-3-{3-[5-(MORPHOLIN-4-YLMETHYL)-1H-BENZIMIDAZOL-2-YL]-1H-PYRAZOL-4-YL}UREA	-7.22	-8.04	-8.58	-9.57	-8.20
JAK2	5WIN	5WIN-SKE-901	4-({5-AMINO-1-[(2,6-DIFLUOROPHENYL)CARBONYL]-1H-1,2,4-TRIAZOL-3-YL}AMINO)BENZENESULFONAMIDE	-7.43	-9.32	-8.17	-9.15	-8.57
JAK2	6AAJ	6AAJ-9T6-1201	4-[[(1S,3R)-5-OXIDANYL-2-ADAMANTYL]AMINO]-1H-PYRROLO[2,3-B]PYRIDINE-5-CARBOXAMIDE	-8.24	-8.73	-9.30	-9.08	-9.51
JAK2	6BBV	6BBV-D7D-1201	N-{CIS-3-[METHYL(7H-PYRROLO[2,3-D]PYRIMIDIN-4-YL)AMINO]CYCLOBUTYL}PROPANE-1-SULFONAMIDE	-7.20	-7.35	-8.53	-7.95	-8.54
JAK2	6BRW	6BRW-5BS-901	4-[(5,10-DIMETHYL-6-OXO-6,10-DIHYDRO-5H-PYRIMIDO[5,4-B]THIENO[3,2-E][1,4]DIAZEPIN-2-YL)AMINO]BENZENESULFONAMIDE	-6.84	-8.88	-9.10	-8.61	-8.52
JAK2	6BS0	6BS0-E4V-901	4-(5-AMINOPYRAZIN-2-YL)-1H-PYRROLO[2,3-B]PYRIDIN-6-AMINE	-5.54	-8.70	-8.87	-8.15	-9.50
JAK2	6BSS	6BSS-4SP-902	O6-CYCLOHEXYLMETHOXY-2-(4'-SULPHAMOYLANILINO) PURINE	-6.57	-9.44	-9.60	-9.29	-10.08
JAK2	6D2I	6D2I-35R-901	1-CYCLOPROPYL-3-{3-[5-(MORPHOLIN-4-YLMETHYL)-1H-BENZIMIDAZOL-2-YL]-1H-PYRAZOL-4-YL}UREA	-7.61	-7.90	-8.61	-8.39	-8.63
JAK2	6G3C	6G3C-EKT-901	2-[[3,5-BIS(FLUORANYL)-4-OXIDANYL-PHENYL]AMINO]-5,7,7-TRIMETHYL-8-(3-METHYLBUTYL)PTERIDIN-6-ONE	-9.10	-9.51	-9.43	-9.17	-9.52
JAK2	6M9H	6M9H-J9D-901	4-({4-AMINO-6-[4-(2-HYDROXYETHYL)-1H-IMIDAZOL-1-YL]PYRIMIDIN-2-YL}AMINO)BENZONITRILE	-6.16	-8.53	-8.67	-8.15	-9.85
JAK2	6OAV	6OAV-M3A-901	5-AMINO-3-[(4-CYANOPHENYL)AMINO]-N-PHENYL-1H-1,2,4-TRIAZOLE-1-CARBOXAMIDE	-8.37	-9.01	-9.25	-9.87	-9.99

JAK2	6OBB	6OBB-M3Y-901	5-AMINO-N-PHENYL-3-[(4-SULFAMOYLPHENYL)AMINO]-1H-1,2,4-TRIAZOLE-1-CARBOXAMIDE	-7.90	-9.25	-9.37	-10.16	-10.30
JAK2	6OBF	6OBF-M4G-902	[4-({5-AMINO-3-[(4-SULFAMOYLPHENYL)AMINO]-1H-1,2,4-TRIAZOLE-1-CARBONYL}AMINO)PHENOXY]ACETIC ACID	-8.63	-9.26	-9.16	-10.00	-10.37
JAK2	6OBL	6OBL-M4P-901	[4-({5-AMINO-3-[(4-CYANOPHENYL)AMINO]-1H-1,2,4-TRIAZOLE-1-CARBONYL}AMINO)PHENOXY]ACETIC ACID	-8.76	-9.51	-10.66	-9.86	-10.73
JAK2	6OCC	6OCC-M57-1002	2-[4-({5-AMINO-3-[(4-SULFAMOYLPHENYL)AMINO]-1H-1,2,4-TRIAZOLE-1-CARBONYL}AMINO)PHENYL]-1,3-OXAZOLE-4-CARBOXYLIC ACID	-9.74	-8.85	-8.79	-9.62	-9.89
JAK2	6TPD	6TPD-QZ8-1201	3-METHYL-4-PHENYL-2,7-DIHYDROPYRAZOLO[3,4-B]PYRIDIN-6-ONE	-7.65	-8.79	-8.54	-9.17	-9.13
JAK2	6VGL	6VGL-RXT-1201	(3R)-3-CYCLOPENTYL-3-[4-(7H-PYRROLO[2,3-D]PYRIMIDIN-4-YL)-1H-PYRAZOL-1-YL]PROPANENITRILE	-8.11	-9.38	-9.77	-8.92	-9.81
JAK2	6VN8	6VN8-3JW-1201	BARICITINIB	-8.33	-9.46	-9.02	-8.83	-9.58
JAK2	6VNB	6VNB-R6P-1201	(3S)-3-CYCLOPENTYL-3-[4-(2-{[4-(PIPERIDIN-4-YL)PHENYL]AMINO}-7H-PYRROLO[2,3-D]PYRIMIDIN-4-YL)-1H-PYRAZOL-1-YL]PROPANENITRILE	-9.85	-8.93	-9.72	-8.92	-9.67
JAK2	6VNC	6VNC-R6V-1201	(3R)-3-CYCLOPENTYL-3-[4-(2-{[4-(PIPERIDIN-4-YL)PHENYL]AMINO}-7H-PYRROLO[2,3-D]PYRIMIDIN-4-YL)-1H-PYRAZOL-1-YL]PROPANENITRILE	-10.00	-9.34	-9.89	-9.07	-9.60
JAK2	6VNE	6VNE-2TA-1201	N-TERT-BUTYL-3-{[5-METHYL-2-({4-[2-(PYRROLIDIN-1-YL)ETHOXY]PHENYL}AMINO)PYRIMIDIN-4-YL]AMINO}BENZENESULFONAMIDE	-7.41	-9.67	-10.13	-9.22	-10.13
JAK2	6VNF	6VNF-R6S-1201	N~4~-[1-(TERT-BUTYLSULFONYL)-2,3-DIHYDRO-1H-INDOL-6-YL]-N~2~-[3-FLUORO-4-(1-METHYLPIPERIDIN-4-YL)PHENYL]-5-METHYLPYRIMIDINE-2,4-DIAMINE	-8.45	-9.33	-9.71	-9.14	-9.54
JAK2	6VNG	6VNG-R6M-1201	N-{2-FLUORO-5-[(2-{[3-FLUORO-4-(1-METHYLPIPERIDIN-4-YL)PHENYL]AMINO}-5-METHYLPYRIMIDIN-4-YL)AMINO]PHENYL}-2-METHYLPROPANE-2-SULFONAMIDE	-7.95	-9.60	-9.87	-8.96	-9.90
JAK2	6VNH	6VNH-XZS-1201	N-{5-[(2-{[3,5-DIFLUORO-4-(1-METHYLPIPERIDIN-4-YL)PHENYL]AMINO}-5-METHYLPYRIMIDIN-4-YL)AMINO]-2-FLUOROPHENYL}-2-METHYLPROPANE-2-SULFONAMIDE	-9.04	-9.40	-9.14	-8.92	-9.15
JAK2	6VNI	6VNI-R61-1201	2-{5-[(2-{[3,5-DIFLUORO-4-(1-METHYLPIPERIDIN-4-YL)PHENYL]AMINO}-5-METHYLPYRIMIDIN-4-YL)AMINO]-2-FLUOROPHENYL}-1LAMBDA~6~,2-THIAZOLIDINE-1,1-DIONE	-8.19	-9.22	-9.88	-8.79	-10.29
JAK2	6VNJ	6VNJ-R5S-1201	3-[4-(2-{[4-(PIPERIDIN-4-YL)PHENYL]AMINO}-6,7-DIHYDRO-5H-PYRROLO[2,3-D]PYRIMIDIN-4-YL)-1H-PYRAZOL-1-YL]PROPANENITRILE	-8.71	-9.17	-8.89	-8.86	-9.58
JAK2	6VNK	6VNK-RXT-1201	(3R)-3-CYCLOPENTYL-3-[4-(7H-PYRROLO[2,3-D]PYRIMIDIN-4-YL)-1H-PYRAZOL-1-YL]PROPANENITRILE	-8.13	-9.53	-10.26	-9.41	-9.71
JAK2	6VNL	6VNL-5W2-1201	4-[[4-[[3-(~{TERT}-BUTYLSULFONYLAMINO)-4-CHLORANYL-PHENYL]AMINO]-5-METHYL-PYRIMIDIN-2-YL]AMINO]-2-FLUORANYL-~{N}-(1-METHYLPIPERIDIN-4-YL)BENZAMIDE	-8.74	-9.46	-9.94	-8.76	-9.82
JAK2	6VNM	6VNM-R5Y-1201	4-[1-(BUT-3-EN-1-YL)-1H-PYRAZOL-4-YL]-N-[4-(PIPERIDIN-4-YL)PHENYL]-7H-PYRROLO[2,3-D]PYRIMIDIN-2-AMINE	-7.72	-9.22	-9.49	-9.06	-9.29
JAK2	6VS3	6VS3-R6V-1201	(3R)-3-CYCLOPENTYL-3-[4-(2-{[4-(PIPERIDIN-4-YL)PHENYL]AMINO}-7H-PYRROLO[2,3-D]PYRIMIDIN-4-YL)-1H-PYRAZOL-1-YL]PROPANENITRILE	-10.25	-9.22	-10.05	-8.86	-10.06
JAK2	6VSN	6VSN-RG4-1201	(3S)-3-CYCLOPENTYL-3-[4-(7H-PYRROLO[2,3-D]PYRIMIDIN-4-YL)-1H-PYRAZOL-1-YL]PROPANENITRILE	-7.77	-9.38	-9.62	-8.40	-9.17

JAK2	6WTN	6WTN-RXT-1204	(3R)-3-CYCLOPENTYL-3-[4-(7H-PYRROLO[2,3-D]PYRIMIDIN-4-YL)-1H-PYRAZOL-1-YL]PROPANENITRILE	-8.29	-9.59	-9.18	-9.14	-9.91
JAK2	6X8E	6X8E-UWP-4000	[3-{4-[6-(1-METHYL-1H-PYRAZOL-4-YL)PYRAZOLO[1,5-A]PYRAZIN-4-YL]-1H-PYRAZOL-1-YL}-1-(2,2,2-TRIFLUOROETHYL)AZETIDIN-3-YL]ACETONITRILE	-9.11	-8.70	-10.18	-9.44	-9.89
JAK2	6XJK	6XJK-V4D-901	4-({4-AMINO-6-[(1H-INDOL-5-YL)OXY]-1,3,5-TRIAZIN-2-YL}AMINO)BENZENE-1-SULFONAMIDE	-7.65	-9.37	-9.59	-9.32	-10.33
JAK2	7F7W	7F7W-36H-1000	2-((1-(2-FLUORO-4-((4-(1-ISOPROPYL-1H-PYRAZOL-4-YL)-5-METHYLPYRIMIDIN-2-YL)AMINO)PHENYL)PIPERIDIN-4-YL)(METHYL)AMINO)ETHAN-1-OL	-7.57	-7.51	-8.83	-7.80	-8.63
JAK2	7JYO	7JYO-VPS-901	3-({4-AMINO-6-[(4-CYANOPHENYL)AMINO]-1,3,5-TRIAZIN-2-YL}OXY)BENZOIC ACID	-7.15	-9.33	-9.27	-9.09	-9.23
JAK2	7JYQ	7JYQ-VPJ-902	N~2~-(4-FLUOROPHENYL)-6-{[(5-{[(OXOLAN-2-YL)METHYL]AMINO}-1,3,4-THIADIAZOL-2-YL)SULFANYL]METHYL}-1,3,5-TRIAZINE-2,4-DIAMINE	-3.63	-9.38	-9.63	-9.50	-8.93
JAK2	7LL5	7LL5-Y5G-1202	{1-(ETHYLSULFONYL)-3-[4-(2-{[4-(1-METHYLPIPERIDIN-4-YL)PHENYL]AMINO}-7H-PYRROLO[2,3-D]PYRIMIDIN-4-YL)-1H-PYRAZOL-1-YL]AZETIDIN-3-YL}ACETONITRILE	-10.63	-9.35	-9.70	-8.79	-9.75
JAK2	7REE	7REE-4LY-1201	[3-(4-{2-[3,5-DIFLUORO-4-(1-METHYL-1,2,3,6-TETRAHYDROPYRIDIN-4-YL)ANILINO]-7H-PYRROLO[2,3-D]PYRIMIDIN-4-YL}-1H-PYRAZOL-1-YL)-1-(ETHANESULFONYL)AZETIDIN-3-YL]ACETONITRILE	-10.48	-9.29	-9.89	-8.87	-9.57
JAK2	7T0P	7T0P-E3W-901	4'-{[5-AMINO-3-(4-SULFAMOYLANILINO)-1H-1,2,4-TRIAZOLE-1-CARBONYL]AMINO}-4-(BENZYLOXY)[1,1'-BIPHENYL]-3-CARBOXYLIC ACID	-9.90	-8.29	-8.79	-9.22	-9.41
JAK2	7TEU	7TEU-I6C-1201	3-{(4S)-2-[(CYCLOPROPANECARBONYL)AMINO]IMIDAZO[1,2-B]PYRIDAZIN-6-YL}-N-{3-[(4-ETHYLPIPERAZIN-1-YL)METHYL]-5-(TRIFLUOROMETHYL)PHENYL}-4-METHYLBENZAMIDE	-12.85	-7.39	-8.67	-6.94	-8.82
JAK2	7UYW	7UYW-OV0-1201	2-(2,6-DIFLUOROPHENYL)-4-[4-(PYRROLIDINE-1-CARBONYL)ANILINO]-5H-PYRROLO[3,4-B]PYRIDIN-5-ONE	-10.02	-8.19	-8.55	-9.28	-9.12
JAK2	8B8N	8B8N-AQG-904	4-{[6-(CYCLOHEXYLMETHOXY)-7H-PURIN-2-YL]AMINO}-N,N-DIETHYLBENZAMIDE	-6.05	-9.31	-9.91	-9.69	-9.46
JAK2	8B8U	8B8U-T7I-902	3,5-DIPHENYL-2-(TRIFLUOROMETHYL)-1~{H}-PYRAZOLO[1,5-A]PYRIMIDIN-7-ONE	-9.50	-9.73	-9.39	-9.50	-9.74
JAK2	8B99	8B99-SKE-1003	4-({5-AMINO-1-[(2,6-DIFLUOROPHENYL)CARBONYL]-1H-1,2,4-TRIAZOL-3-YL}AMINO)BENZENESULFONAMIDE	-8.49	-10.15	-9.75	-10.38	-9.62
JAK2	8B9E	8B9E-Q7F-1003	6-[[METHYL-[(3-METHYLTHIOPHEN-2-YL)METHYL]AMINO]METHYL]-~{N}4-PHENYL-1,3,5-TRIAZINE-2,4-DIAMINE	-7.19	-9.87	-9.76	-9.97	-10.25
JAK2	8B9H	8B9H-Q7F-901	6-[[METHYL-[(3-METHYLTHIOPHEN-2-YL)METHYL]AMINO]METHYL]-~{N}4-PHENYL-1,3,5-TRIAZINE-2,4-DIAMINE	-7.10	-9.44	-8.90	-9.62	-9.66
JAK2	8BA2	8BA2-Q9X-907	6-[[(5-BROMANYLTHIOPHEN-2-YL)METHYL-METHYL-AMINO]METHYL]-~{N}4-(4-METHYLPHENYL)-1,3,5-TRIAZINE-2,4-DIAMINE	-6.88	-9.39	-9.65	-9.50	-10.03
JAK2	8BA3	8BA3-Q8U-901	1-(3,4-DIAZATRICYCLO[9.4.0.0^{2,7}]PENTADECA-1(11),2(7),3,5,12,14-HEXAEN-5-YL)-~{N}3-[(7~{S})-7-PYRROLIDIN-1-YL-6,7,8,9-TETRAHYDRO-5~{H}-BENZO[7]ANNULEN-3-YL]-1,2,4-TRIAZOLE-3,5-DIAMINE	-12.27	-7.99	-8.20	-8.56	-8.29
JAK2	8BA4	8BA4-Q8U-901	1-(3,4-DIAZATRICYCLO[9.4.0.0^{2,7}]PENTADECA-	-10.44	-7.81	-8.25	-8.47	-9.77

			1(11),2(7),3,5,12,14-HEXAEN-5-YL)-~{N}3-[(7~{S})-7-PYRROLIDIN-1-YL-6,7,8,9-TETRAHYDRO-5~{H}-BENZO[7]ANNULEN-3-YL]-1,2,4-TRIAZOLE-3,5-DIAMINE					
JAK2	8BAB	8BAB-Q8N-908	6-[(1-METHYLIMIDAZOL-2-YL)SULFANYLMETHYL]-~{N}4-(3-METHYLPHENYL)-1,3,5-TRIAZINE-2,4-DIAMINE	-2.37	-9.77	-9.53	-9.79	-9.78
JAK2	8BAK	8BAK-AD5-906	N~6~-CYCLOHEXYL-N~2~-(4-MORPHOLIN-4-YLPHENYL)-9H-PURINE-2,6-DIAMINE	-7.10	-10.22	-10.26	-10.60	-11.30
JAK2	8BM2	8BM2-QQC-1201	3-[(4-CHLORANYL-2-FLUORANYL-PHENYL)METHYL]-2-METHYL-~{N}-(5-METHYL-1~{H}-PYRAZOL-3-YL)-8-(MORPHOLIN-4-YLMETHYL)IMIDAZO[1,2-B]PYRIDAZIN-6-AMINE	-10.02	-8.61	-10.31	-8.62	-10.28
JAK2	8BPV	8BPV-6T3-1201	11-(2-PYRROLIDIN-1-YL-ETHOXY)-14,19-DIOXA-5,7,26-TRIAZA-TETRACYCLO[19.3.1.1(2,6).1(8,12)]HEPTACOSA-1(25),2(26),3,5,8,10,12(27),16,21,23-DECAENE	-10.76	-9.47	-9.01	-10.30	-9.40
JAK2	8BPW	8BPW-2V9-1201	LESTAURTINIB	-12.99	-8.81	-10.43	-10.13	-9.72
JAK2	8BX6	8BX6-S5I-1201	CERDULATINIB	-6.61	-8.84	-8.35	-10.37	-8.07
JAK2	8BX9	8BX9-S59-1201	ILGINATINIB	-8.34	-9.87	-10.46	-9.25	-10.50
JAK2	8BXC	8BXC-S4R-1201	ITACITINIB	-11.32	-9.48	-10.27	-9.54	-9.94
JAK2	8BXH	8BXH-C87-1201	MOMELOTINIB	-8.67	-10.19	-11.06	-10.13	-11.43
JAK2	8C08	8C08-ATP-901	ADENOSINE-5'-TRIPHOSPHATE	-7.04	-9.64	-10.36	-10.13	-10.56
JAK2	8C0A	8C0A-T7I-902	3,5-DIPHENYL-2-(TRIFLUOROMETHYL)-1~{H}-PYRAZOLO[1,5-A]PYRIMIDIN-7-ONE	-9.86	-9.52	-8.71	-9.33	-8.70
JAK2	8EX0	8EX0-AQG-901	4-{[6-(CYCLOHEXYLMETHOXY)-7H-PURIN-2-YL]AMINO}-N,N-DIETHYLBENZAMIDE	-6.82	-9.24	-10.19	-9.75	-9.91
JAK2	8EX1	8EX1-AD5-901	N~6~-CYCLOHEXYL-N~2~-(4-MORPHOLIN-4-YLPHENYL)-9H-PURINE-2,6-DIAMINE	-7.30	-9.42	-9.43	-9.09	-9.02
JAK2	8EX2	8EX2-Q2Q-901	3,5-DIPHENYL-2-(TRIFLUOROMETHYL)-6~{H}-PYRAZOLO[1,5-A]PYRIMIDIN-7-ONE	-9.52	-9.38	-8.57	-9.10	-8.52
JAK2	8G8O	8G8O-YT0-1201	[1-{5-METHYL-2-[(3-METHYL-1,2-THIAZOL-5-YL)AMINO]PYRIMIDIN-4-YL}-3-(4-METHYLPIPERAZIN-1-YL)AZETIDIN-3-YL]ACETONITRILE	-7.48	-8.90	-11.15	-9.55	-10.69
JAK2	8G8X	8G8X-YT8-1201	3-CYCLOPROPYL-1-{5-METHYL-2-[(3-METHYL-1,2-THIAZOL-5-YL)AMINO]PYRIMIDIN-4-YL}AZETIDIN-3-OL	-7.04	-9.49	-10.73	-9.74	-10.50

Table S5. Individual AutoDockVina scores for the ligands docked to the predicted target protein MAPK8. PDB ID—identificator of PDB structure of the target protein with co-crystallized ligand (CCL). CCL ID—combination of PDB ID, residue name, and residue number that identifies CCL structure in the source PDB file. R, S—enantiomers of Les-6547 and Les-6557, respectively. Values in the "CCL" column represent individual Vina scores for different CCLs bound to a different conformation of the predicted target, evaluated by AutoDock Vina in score-only mode without performing docking. The remaining four columns contain individual Vina docking scores of Les-6547 and Les-6557 enantiomers docked to the same conformations of the target that bind CCLs. For each of n

Target Protein	PDB ID	CCL ID	CCL Name	Vina scores, kkal/mol				
				CCL	Les-6547		Les-6557	
					R	S	R	S
MAPK8	1UKI	1UKI-537-0	2,6-DIHYDROANTHRA/1,9-CD/PYRAZOL-6-ONE	-7.30	-9.18	-10.09	-9.45	-9.47
MAPK8	2G01	2G01-73Q-1001	6-CHLORO-9-HYDROXY-1,3-DIMETHYL-1,9-DIHYDRO-4H-PYRAZOLO[3,4-B]QUINOLIN-4-ONE	-6.06	-9.40	-9.50	-9.77	-9.64
MAPK8	2GMX	2GMX-877-901	N-(4-AMINO-5-CYANO-6-ETHOXYPYRIDIN-2-YL)-2-(4-BROMO-2,5-DIMETHOXYPHENYL)ACETAMIDE	-6.03	-10.18	-9.20	-9.83	-9.77
MAPK8	2H96	2H96-893-901	5-CYANO-N-(2,5-DIMETHOXYBENZYL)-6-ETHOXYPYRIDINE-2-CARBOXAMIDE	-6.48	-9.05	-7.58	-9.04	-8.69
MAPK8	2NO3	2NO3-859-901	2-({2-[(3-HYDROXYPHENYL)AMINO]PYRIMIDIN-4-YL}AMINO)BENZAMIDE	-5.24	-8.86	-7.53	-9.20	-8.12
MAPK8	2XS0	2XS0-ANP-1367	PHOSPHOAMINOPHOSPHONIC ACID-ADENYLATE ESTER	-7.13	-8.33	-7.46	-8.34	-9.32
MAPK8	3O2M	3O2M-46A-701	N-BUTYL-4,6-DIMETHYL-N-{[2'-(2H-TETRAZOL-5-YL)BIPHENYL-4-YL]METHYL}PYRIMIDIN-2-AMINE	-7.35	-10.69	-9.47	-9.98	-9.51
MAPK8	3PZE	3PZE-CFK-1	3-(CARBAMOYLAMINO)-5-PHENYLTHIOPHENE-2-CARBOXAMIDE	-7.07	-8.12	-9.13	-9.02	-7.94
MAPK8	3V3V	3V3V-MYU-401	3,5,6,7-TETRAHYDROXY-2-(3,4-DIHYDROXYPHENYL)-4H-CHROMEN-4-ONE	-8.10	-8.66	-7.36	-8.41	-9.09
MAPK8	4E73	4E73-0NR-401	METHYL 3-(4-{[(1R,2S,3S,5S,7S)-5-AMINOTRICYCLO[3.3.1.1~3,7~]DEC-2-YL]CARBAMOYL}BENZYL)-4-OXO-1-PHENYL-1,4-DIHYDRO-1,8-NAPHTHYRIDINE-2-CARBOXYLATE	-8.79	-9.09	-8.58	-9.36	-8.62
MAPK8	4G1W	4G1W-G1W-401	METHYL 7-FLUORO-3-{4-[(2-HYDROXYETHYL)SULFONYL]BENZYL}-4-OXO-1-PHENYL-1,4-DIHYDROQUINOLINE-2-CARBOXYLATE	-8.02	-9.14	-9.12	-9.06	-8.68
MAPK8	4HYS	4HYS-1BJ-401	TRANS-4-{[4-(1H-INDAZOL-1-YL)PYRIMIDIN-2-YL]AMINO}CYCLOHEXANOL	-6.35	-8.23	-8.63	-8.31	-8.59
MAPK8	4HYU	4HYU-1BK-401	TRANS-4-[(4-{4-[3-(METHYLSULFONYL)PROPOXY]-1H-INDAZOL-1-YL}PYRIMIDIN-2-YL)AMINO]CYCLOHEXANOL	-7.22	-8.57	-8.69	-8.55	-8.85
MAPK8	4IZY	4IZY-1J2-401	TRANS-4-[(4-{4-[4-(METHYLSULFONYL)PIPERIDIN-1-YL]-1H-INDOL-1-YL}PYRIMIDIN-2-YL)AMINO]CYCLOHEXANOL	-8.51	-8.54	-8.64	-8.57	-9.02
MAPK8	4UX9	4UX9-ANP-1000	PHOSPHOAMINOPHOSPHONIC ACID-ADENYLATE ESTER	-5.70	-7.91	-7.42	-7.86	-7.25
MAPK8	5LW1	5LW1-ADN-401	ADENOSINE	-5.33	-9.54	-9.71	-10.12	-9.27
MAPK8	6ZR5	6ZR5-ANP-402	PHOSPHOAMINOPHOSPHONIC ACID-ADENYLATE ESTER	-6.81	-7.77	-9.82	-7.58	-8.44

target conformations, the most favorable docking score was selected. The CCLs were removed from the protein structures as the latter were prepared for docking.

Table S6. Individual AutoDockVina scores for the ligands docked to the predicted target protein MKNK2. PDB ID—identificator of PDB structure of the target protein with co-crystallized ligand (CCL). CCL ID—combination of PDB ID, residue name, and residue number that identifies CCL structure in the source PDB file. R, S—enantiomers of Les-6547 and Les-6557, respectively. Values in the "CCL" column represent individual Vina scores for different CCLs bound to a different conformation of predicted target, evaluated by AutoDock Vina in score-only mode without performing docking. The remaining four columns contain individual Vina docking scores of Les-6547 and Les-6557 enantiomers docked to the same conformations of the target that bind CCLs. For each of n target

Target Protein	PDB ID	CCL ID	CCL Name	Vina scores, kkal/mol				
				CCL	Les-6547		Les-6557	
					R	S	R	S
MKNK2	2HW7	2HW7-STU-31	STAUROSPORINE	-11.14	-9.01	-9.77	-8.86	-9.52
MKNK2	6CJ5	6CJ5-F4G-402	3-(PYRIDIN-3-YL)IMIDAZO[1,2-A]PYRIDINE-8-CARBOXAMIDE	-5.44	-9.31	-8.68	-8.91	-9.45
MKNK2	6CJE	6CJE-F4A-402	4-[(9H-PURIN-6-YL)AMINO]BENZAMIDE	-5.82	-9.01	-9.45	-9.73	-8.82
MKNK2	6CJH	6CJH-ET8-402	3-PHENYL-5-(PYRIDIN-4-YL)-1H-INDAZOLE	-4.71	-9.11	-8.50	-10.05	-9.85
MKNK2	6CJY	6CJY-F4J-402	5-[(7H-PURIN-6-YL)AMINO]-1H-ISOINDOL-1-ONE	-5.61	-9.01	-10.19	-10.34	-10.08
MKNK2	6CK3	6CK3-F67-402	(3R)-3-METHYL-5-[(PYRIMIDIN-4-YL)AMINO]-2,3-DIHYDRO-1H-ISOINDOL-1-ONE	-5.92	-9.12	-8.04	-9.50	-9.10
MKNK2	6CK6	6CK6-N45-402	6'-[(6-AMINOPYRIMIDIN-4-YL)AMINO]-8'-METHYL-2'H-SPIRO[CYCLOHEXANE-1,3'-IMIDAZO[1,5-A]PYRIDINE]-1',5'-DIONE	-7.24	-9.16	-7.61	-9.24	-7.32
MKNK2	6CKI	6CKI-FZJ-402	3,3-DIMETHYL-6-[(PYRIMIDIN-4-YL)AMINO]-2,3-DIHYDROIMIDAZO[1,5-A]PYRIDINE-1,5-DIONE	-7.67	-8.90	-6.98	-8.63	-7.73
MKNK2	6JLR	6JLR-BV9-401	4-[5-(1-METHYLPYRAZOL-4-YL)PYRIDIN-3-YL]BENZAMIDE	-7.67	-8.86	-9.46	-8.53	-8.68
MKNK2	8P9B	8P9B-X8K-402	4-[6-(4-MORPHOLIN-4-YLCARBONYLPHENYL)IMIDAZO[1,2-A]PYRIDIN-3-YL]BENZENECARBONITRILE	-10.69	-9.00	-8.81	-9.49	-8.44

conformations, the most favorable docking score was selected. The CCLs were removed from the protein structures as the latter were prepared for docking.

Table S7. Individual AutoDockVina scores for the ligands docked to the predicted target protein MMP9. PDB ID—identificator of PDB structure of the target protein with co-crystallized ligand (CCL). CCL ID—combination of PDB ID, residue name, and residue number that identifies CCL structure in the source PDB file. R, S—enantiomers of Les-6547 and Les-6557, respectively. Values in the "CCL" column represent individual Vina scores for different CCLs bound to a different conformation of predicted target, evaluated by AutoDock Vina in score-only mode without performing docking. The remaining four columns contain individual Vina docking scores of Les-6547 and Les-6557 enantiomers docked to the same conformations of the target that bind CCLs. For each of n target

Target Protein	PDB ID	CCL ID	CCL Name	Vina scores, kkal/mol				
				CCL	Les-6547		Les-6557	
					R	S	R	S
MMP9	1GKC	1GKC-NFH-1448	N~2~-[(2R)-2-{[FORMYL(HYDROXY)AMINO]METHYL}-4-METHYLPENTANOYL]-N,3-DIMETHYL-L-VALINAMIDE	-5.13	-8.80	-7.77	-7.67	-7.85
MMP9	2OVX	2OVX-4MR-501	5-(4-PHENOXYPHENYL)-5-(4-PYRIMIDIN-2-YLPIPERAZIN-1-YL)PYRIMIDINE-2,4,6(2H,3H)-TRIONE	-10.55	-10.44	-9.98	-9.96	-9.99
MMP9	2OVZ	2OVZ-5MR-501	NALPHA-{(2S)-3-[(S)-HYDROXY(PHENYL)PHOSPHORYL]-2-[(3-PHENYLISOXAZOL-5-YL)METHYL]PROPANOYL}-L-TRYPTOPHANAMIDE	-9.00	-10.09	-8.12	-8.65	-8.62
MMP9	2OW0	2OW0-6MR-501	N-[(4'-IODOBIPHENYL-4-YL)SULFONYL]-D-TRYPTOPHAN	-8.54	-10.20	-9.20	-9.91	-8.99
MMP9	2OW1	2OW1-7MR-501	(2R)-2-AMINO-3,3,3-TRIFLUORO-N-HYDROXY-2-{[(4-PHENOXYPHENYL)SULFONYL]METHYL}PROPANAMIDE	-8.55	-9.74	-8.06	-9.18	-7.95
MMP9	2OW2	2OW2-8MR-501	(3R)-4,4-DIFLUORO-3-[(4-METHOXYPHENYL)SULFONYL]BUTANOIC ACID	-5.78	-8.97	-9.59	-9.29	-9.51
MMP9	5UE4	5UE4-5XQ-307	~{N}-[5-[2-[(2-METHOXYPHENYL)AMINO]-1,3-THIAZOL-4-YL]-4-METHYL-1,3-THIAZOL-2-YL]ETHANAMIDE	-6.67	-8.44	-7.40	-8.09	-7.30
MMP9	8K5V	8K5V-VOC-306	6,7-DIHYDRO-4H-[1,3]OXAZOLO[4,5-C]PYRIDIN-5-YL-(7-ETHYL-2H-INDAZOL-3-YL)METHANONE	-7.68	-7.70	-8.28	-7.38	-8.18
MMP9	8K5W	8K5W-VOO-306	2-[[5-FLUORANYL-7-(METHYLAMINO)-1H-INDOL-2-YL]CARBONYL]-N-(2-PYRROL-1-YLETHYL)-3,4-DIHYDRO-1H-ISOQUINOLINE-7-CARBOXAMIDE	-7.83	-9.07	-8.09	-8.58	-8.66
MMP9	8K5X	8K5X-VOZ-306	(6-CYCLOPROPYL-1~{H}-INDOL-2-YL)-(5,7,8,9-TETRAHYDROPYRIDO[4,3-C]AZEPIN-6-YL)METHANONE	-4.38	-7.98	-6.31	-7.92	-4.63

conformations, the most favorable docking score was selected. The CCLs were removed from the protein structures as the latter were prepared for docking.
